# Single-cell multiomic comparison of mouse and rat spermatogenesis reveals gene regulatory networks conserved for over 20 million years

**DOI:** 10.1016/j.stemcr.2025.102449

**Published:** 2025-03-13

**Authors:** Eoin C. Whelan, John J. Swain, Jonathan H. Sussman, David Smith, Fan Yang, Antonia Rotolo, Mary R. Avarbock, Clara Malekshahi, Enrico Radaelli, Daniel P. Beiting, Ralph L. Brinster

**Affiliations:** 1 Department of Biomedical Sciences, University of Pennsylvania, School of Veterinary Medicine, Philadelphia, PA, USA; 2 Department of Pathobiology, University of Pennsylvania, School of Veterinary Medicine, Philadelphia, PA, USA; 3 Children’s Hospital of Philadelphia, Philadelphia, PA, USA; 4 Medical Scientist Training Program, Perelman School of Medicine, University of Pennsylvania, Philadelphia, PA, USA; 5 Graduate Group in Genomics and Computational Biology, Perelman School of Medicine, University of Pennsylvania, Philadelphia, PA, USA

**Keywords:** spermatogenesis, germ cells, multiomic, single-cell, RNA sequencing, ATAC, differentiation, mouse, rat

## Abstract

Spermatogenesis is driven by dramatic changes in chromatin regulation, gene transcription, and protein expression. To assess the mechanistic bases for these developmental changes, we utilized multiomic single-cell/nucleus RNA sequencing (sc/snRNA-seq) and single-nucleus assay for transposase-accessible chromatin with sequencing (snATAC-seq) to identify chromatin changes associated with transcription in adult mouse and rat testes. We characterized the relationships between the transcriptomes and chromatin of both species, including the divergent expression of *Id4* in spermatogonial stem cells between species. Promoter accessibility and gene expression showed the greatest association during meiosis in both species. We mapped the cross-species conservation of putative regulatory regions for key spermatogenic genes, including *Cd9* and *Spam1*, and investigated correlations and disconnects in chromatin accessibility, gene expression, and protein expression via antibody-derived tags. Using a gene regulatory network (GRN) model, we identified 40 core regulons conserved between mouse and rat germ cells, highlighting the relevance of chromatin-related factors in regulating the transcription of canonical genes across spermatogenesis.

## Highlights

• Single-cell/nucleus multiomic assays shed light on mouse and rat spermatogenesis
• Conserved gene and lncRNA expression is tightly regulated in both species
• Dynamic changes in chromatin accessibility accompany germ cell differentiation
• 40 conserved transcription factors regulate a network of genes across spermatogenesis

## Graphical abstract

**Figure:**
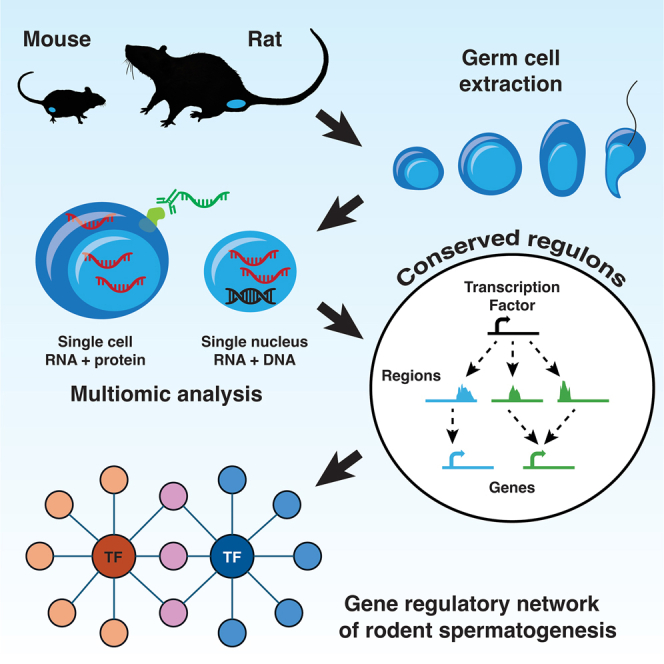

## Teaser

Whelan and colleagues employed single-cell multiomic assays to characterize RNA expression and chromatin accessibility along with select proteins in mouse and rat germ cells. They mapped the cross-species conservation of putative regulatory regions and uncovered a gene regulatory network of 40 key transcription factors conserved between the two species that control the expression of genes across spermatogenesis.

## Introduction

During spermatogenesis, undifferentiated spermatogonial stem cells (SSCs) differentiate to produce all male germ cells in the adult testis. Proliferating mitotic spermatogonia differentiate into spermatocytes that undergo meiosis to become spermatids that mature into spermatozoa (Russell et al., 1993). The complex cellular changes accompanying spermatogenesis are driven by complex changes in gene expression, as evidenced by single-cell mRNA sequencing (scRNA-seq) throughout spermatogenesis in mouse (Green et al., 2018; Hermann et al., 2018; La et al., 2018; Lukassen et al., 2018; Ernst et al., 2019; Grive et al., 2019; Jung et al., 2019), human (Guo et al., 2018; Hermann et al., 2018; Wang et al., 2018; Sohni et al., 2019; Salehi and Totonchi 2023; Li et al., 2024; Wu et al., 2024), and other mammalian species including macaques (Lau et al., 2020; Shami et al., 2020), sheep (Yang et al., 2021; Wu et al., 2022a), pigs (Zhang et al., 2021, 2022a), dairy goats (Yu et al., 2021), buffalo (Huang et al., 2023), yak (Mipam et al., 2023; Wang et al., 2023), and giant pandas (Zheng et al., 2022). However, despite its ability to resolve differentiation trajectories with high resolution, scRNA-seq is unable to identify gene regulatory mechanisms or post-transcriptional impacts on protein abundance that could influence spermatogenesis. Single-cell epigenetic and proteomic studies of mammalian spermatogenesis (Wu et al., 2022b; Zhang et al., 2022b) have shed light on the regulatory and post-transcriptional mechanisms of spermatogenesis, but, to date, an integrated and comprehensive cross-species analysis of changes in chromatin structure, transcription, and protein expression has not yet been undertaken. Both mouse and rat are long-standing models of mammalian spermatogenesis, and the developmental process from stem cells to sperm has been firmly established (Russell et al., 1993), so these are vital species to dissect the molecular events driving differentiation.

In this work, we have established a multiomic analysis of mouse and rat spermatogenesis, comparing and contrasting multi-modal fingerprints of cell states between the two species. Specifically, we used single-cell/nucleus RNA sequencing (sc/snRNA-seq) and single-nucleus assay for transposase-accessible chromatin with sequencing (snATAC-seq) jointly recorded in the same cells, which provided an unprecedented comparison of the gene regulatory mechanisms driving transcriptomic shifts during spermatogenesis. We found regulatory regions that correlated with gene expression of known germ cell-related genes. In addition, we applied cellular indexing of transcriptomes and epitopes by sequencing (CITE-seq) in order to jointly capture global cellular transcription with selected protein expression. These joint profiling techniques are particularly important for assessing the mechanistic basis of spermiogenesis, as a variety of post-transcriptional controls can delay translation of spermiogenic genes, thus de-coupling transcript and protein levels. This study elucidates a core set of conserved regulons that are responsible for regulating multiple steps of the spermatogenic trajectory.

## Results

**Figure 1:**
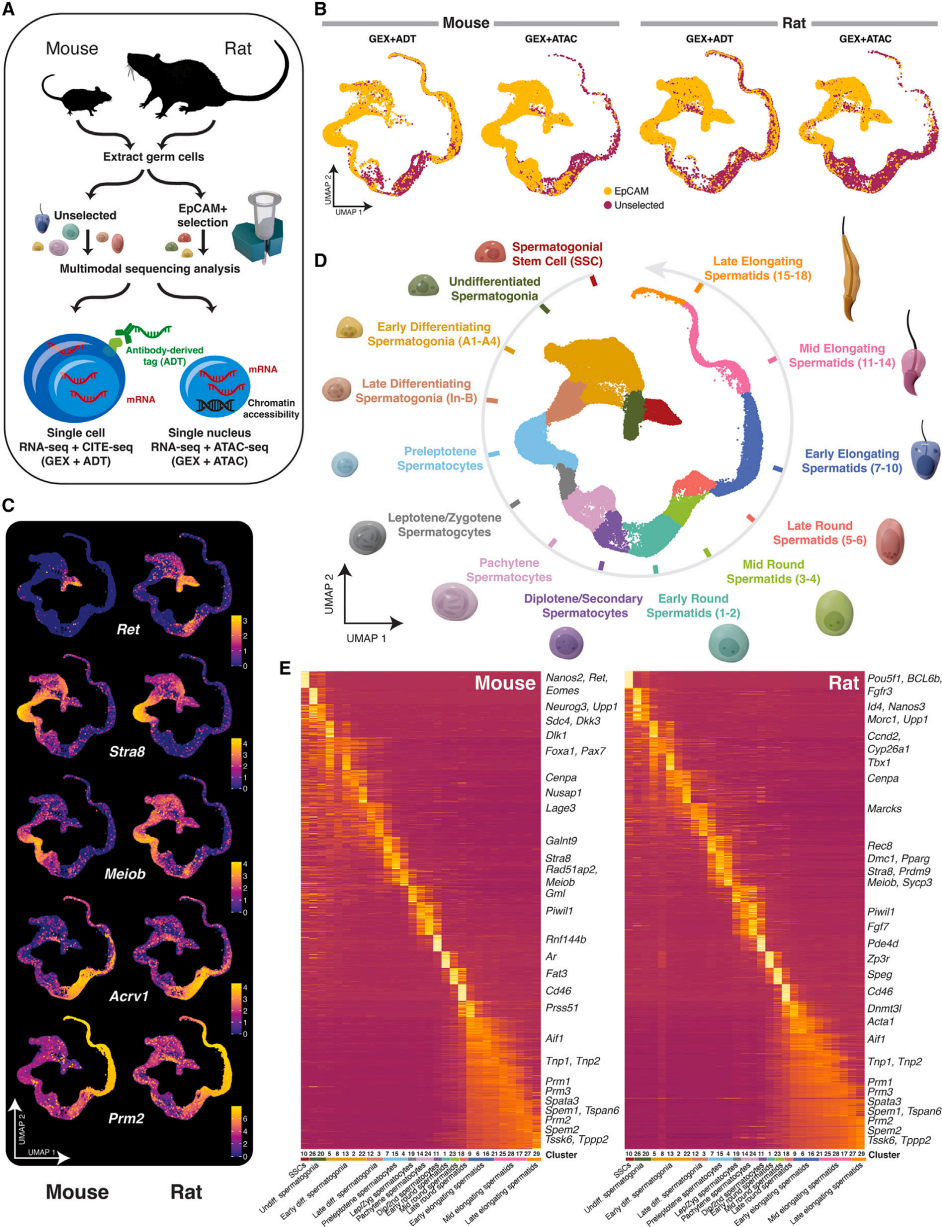
Overview of rodent spermatogenesis (A) Experimental design. Unselected and EpCAM-enriched mouse and rat testicular cells were encapsulated as either nuclei or whole cells for snRNA/ATAC-seq multiomic profiling (*n* = 6 mice and 4 rats) and CITE-seq (*n* = 2 mice and 9 rats), respectively. (B) UMAP projection of unselected and EpCAM+ germ cells from each assay for each species. (C) Normalized RNA expression of key genes involved in spermatogenesis. (D) Cell types assigned after unbiased clustering of integrated mouse and rat germ cells. (E) Heatmap showing gene expression for top 30 genes for each unbiased cluster. Cell type assignments are shown below cluster numbers.

To mechanistically assess the molecular events accompanying spermatogenesis, testis cells from adult *Rattus norvegicus* as well as adult *Mus musculus* were isolated and analyzed along one of two single-cell workflows: (1) as nuclei jointly assayed for gene expression via snRNA+ATAC-seq and (2) as whole cells jointly assayed for gene and protein expression using CITE-seq (Figure 1A). Taken together, these assays enabled us to directly connect epigenetic changes, gene transcription, and selected protein expression within single cells.

### Unbiased scRNA-seq clustering recapitulates spermatogenic processes

To determine the populations of germ cells captured by the single-cell multiomic assays in an unbiased fashion, the shared sc/snRNA-seq assay was used to computationally integrate samples, and cells were clustered and manually annotated based on their gene expression patterns. Somatic cells were removed from the analysis (Figure S1). We captured 23,000 and 17,544 cells by snRNA+ATAC-seq and 17,489 and 54,120 cells by CITE-seq for mouse and rat, respectively (Figure 1B). To adequately resolve the molecular profiles of rare pre-meiotic cells, especially SSCs that comprise only 0.03% of mouse testis cells (Tegelenbosch and de Rooij 1993), we applied these workflows to EpCAM-selected cells known to be enriched for germ cell progenitors (Whelan et al., 2022) as well as unselected mouse and rat testis cells in which post-meiotic cells make up the majority of germ states (Figure 1B). Reproducibility between and within individuals was high across assays (Figures S2A–S2K). Integrated datasets were then clustered in an unsupervised manner by species (Figures S2L and S2M). To improve the resolution of each cell differentiation stage and capture the entire process of spermatogenesis with no discontinuations, we integrated our multimodal datasets for both species based on homologous genes and characterized the transcriptome of each differentiation state based on both the nuclear and whole-cell assays. We used an analysis of unspliced RNA as a surrogate for nascent RNA to ensure that the integrated analysis based on nuclear and whole-cell mRNA was faithful to the cell types designated (Figure S3).

Populations displayed known transcribed markers of germ cell differentiation, showing high degree of conservation between mouse and rat in stem cell markers such as *Nanos2* and *Ret*, as well as genes involved in spermatogonial differentiation (*Kit* and *Stra8*), meiosis (*Sycp3* and *Meiob*), and spermiogenesis (*Acrv1*, *Tnp1*, and *Prm2*) (Figure 1C). Certain genes showed repeated stage-specific expression such as *Stra8*, which was detected in both differentiating spermatogonia and preleptotene spermatocytes in mouse and rat. We manually labeled the cell clusters to delineate discrete stages of germ cell maturation based on gene expression, yielding a single unbroken progression along the differentiation pathway of both rat and mouse germ cells (Figures 1D and S4A). All cell types were captured in both assays, with the exception that the terminal stages of germ cell differentiation (e.g., elongating spermatids) were sparse in the single-nucleus data, which we speculate may be due to loss of nuclear integrity of late-stage cells during detergent-based lysis. We observed strong concordance between mouse and rat clusters, indicating that corresponding cell types shared similar gene expression profiles in the two species (Figure 1E).

### Transcriptomic similarities between mouse and rat germ cells

**Figure 2:**
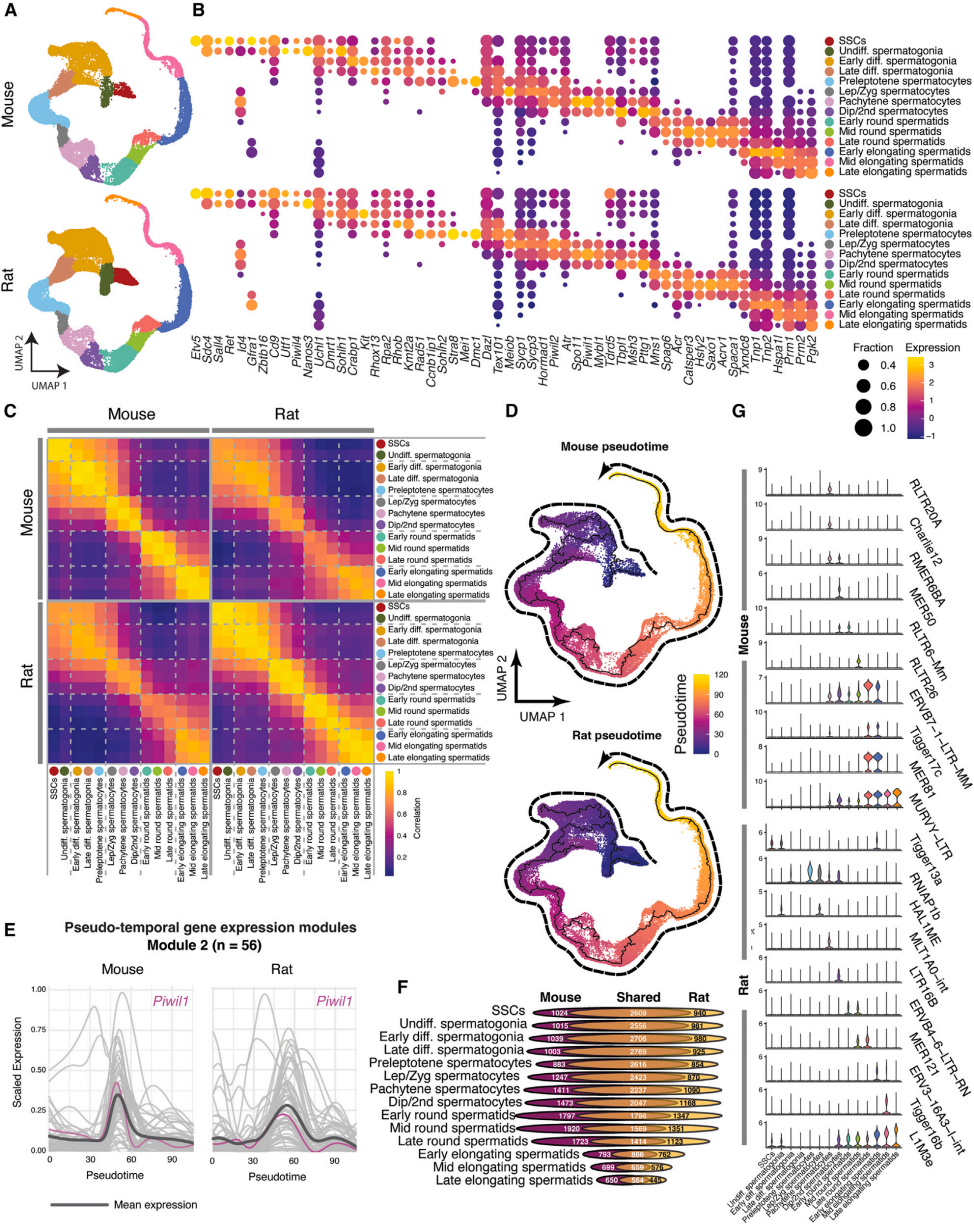
Differentiation of rodent germ cells (A) UMAP projection of mouse and rat spermatogenic lineages (all panels in this figure are based on integrated RNA data from 8 mice and 13 rats). (B) Gene expression of key spermatogenesis genes by cell type. Normalized gene expression is represented by color, whereas the fraction expression of the gene within each cell type is shown (fractions below 0.2 are not shown). (C) Pearson’s correlation of mouse and rat gene expression by cell type between and within species. (D) Pseudotime for mouse and rat germ cell progression overlaid on UMAP projection. (E) Representative gene expression module showing genes with similar expression profiles across pseudotime and correlated between species (r > 0.9, Pearson’s correlation). *Piwil1* expression is highlighted in magenta. Mean expression of all genes is shown by the black line. (F) Number of expressed genes (normalized expression >0.5) in each species as a scaled Venn diagram per cell type. (G) Transposable element (TE) expression by cell type for select subfamilies.

While spermatogenesis in the rat is similar enough to mouse that stem cells from the rat can be transplanted into the mouse and produce all differentiating cell types including sperm (Ryu et al., 2005; Whelan et al., 2022), there remain key differences in cell morphology and seminiferous cycling (Russell et al., 1993). All identified germ cell clusters were present in both mouse and rat (Figure 2A), each displaying a progression of known spermatogenesis markers at appropriate stages, conserved between mice and rats (Figure 2B). Each germ cell type displayed a distinct transcriptional identity as evidenced by differentially expressed genes between stages (Figure S4B). To assess the similarities in transcriptional features during mouse and rat spermatogenesis, we evaluated the correlation of expressed genes between cell types within and between species (Figure 2C). Within species, both mice and rats showed a similar pattern of groupings corresponding to mitotic, meiotic, and spermiogenic phases of spermatogenesis. Within the mitotic phase, a subdivision between undifferentiated and differentiated cells was observed, and spermatids could be divided into round and elongating spermatids, although in both cases these distinctions were more apparent in mouse than rat. Correlations were slightly lower when compared between species, but the overall pattern of cell types was retained. Moreover, despite the differences between mouse and rat spermatogenesis at a morphological level, cell type correlation was highest between any given mouse cell type and its rat counterpart than compared to any different mouse cell type, and likewise for the rat.

Germ cells in both species formed a single progression of cells. This trajectory was assessed by implementing a pseudotime analysis—cells from each species’ trajectory were ordered from least differentiated to most differentiated. This allowed for an unbiased ordering of cells to view changes over time in gene expression and chromatin accessibility (Figure 2D). It also allowed for the grouping of genes into modules of genes that are correlated between species and co-regulated across pseudotime (Figures 2E and S4C). Many expressed genes were shared between mouse and rat within cell types, although both the total number of genes expressed and the proportion of shared genes were reduced in later stages of spermatogenesis (Figure 2F; Table S1).

To evaluate the expression of transposable elements (TEs), we employed SoloTE (Rodríguez-Quiroz and Valdebenito-Maturana 2022) to characterize subfamilies of TEs differentially expressed across spermatogenesis (Figure 2G). No subfamilies were specific to spermatogonia in mouse, although a few, such as Tigger13a, were found in rat. However, starting with the onset of meiosis, stage-specific expression of TEs was observed with narrow expression such as Charlie12 and MLT1A0-int in mouse and rat pachytene spermatocytes, respectively, and MER50 and LTR16B in mouse and rat diplotene/secondary spermatocytes. Interestingly, despite the relative transcriptional repression in spermatids, a number of stage-specific TEs can be observed in both species such as Tigger17c in mouse and Tigger16b in rat.

### Signaling networks and long non-coding RNA expression are conserved in mouse and rat spermatogenesis

**Figure 3:**
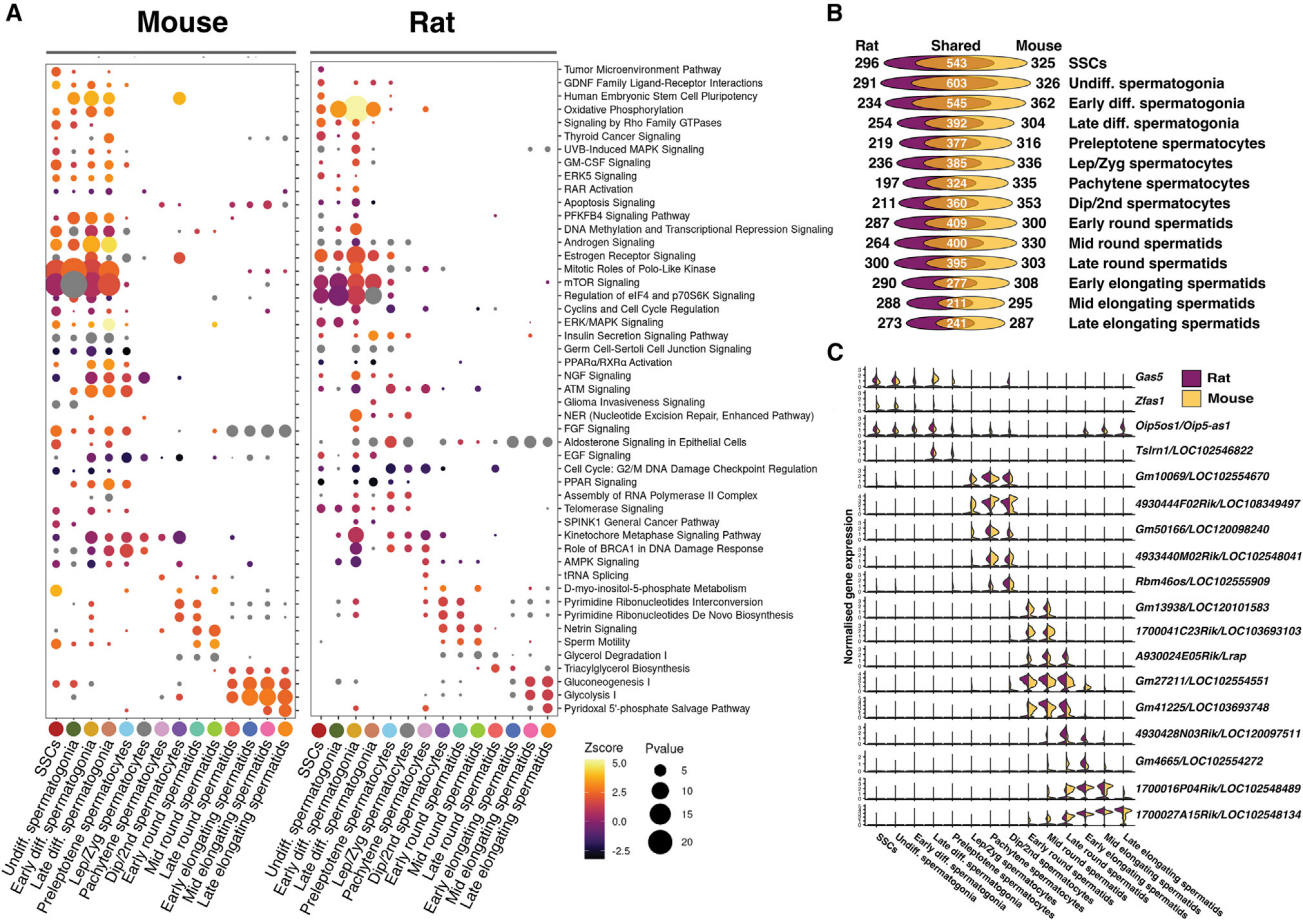
Rodent spermatogenesis involves conserved lncRNA expression and signaling pathways (A) Pathways enriched at each stage of spermatogenesis for mouse (left) and rat (right) determined using an Ingenuity Pathway Analysis based on differentially expressed genes at each stage. *Z* score is denoted by color and −log_10_*p* value is shown by dot size. (B) Number of expressed lncRNAs (normalized expression >0.5) in each species, matched across species using a genomic liftover, as a scaled Venn diagram per cell type. (C) Normalized expression of select lncRNAs with conserved expression patterns between species. All data in this figure were generated from integrated sn/scRNA-seq profiling (*n* = 8 mice and 13 rats).

To further understand the changes transpiring across rodent spermatogenesis, we studied the pathways involved at each stage using an Ingenuity Pathway Analysis with stage-specific differentially expressed genes. Stem cells showed an enrichment of pathways associated with stem cell pluripotency, glial-derived neurotropic factor (GDNF) signaling, and mitogen-activated protein kinase (MAPK) signaling, as expected from previous studies examining the proliferation of SSCs in culture (Ishii et al., 2012; Yang et al., 2021) (Figure 3A). Undifferentiated and differentiating spermatogonia showed enrichment for cell cycle regulation and oxidative phosphorylation, consistent with their proliferating nature. Retinoic acid (RA) signaling was apparent in both spermatogonia and preleptotene spermatocytes, corresponding to the stage-dependent and RA-responsive expression of *Stra8* in differentiating spermatogonia (Gewiss et al., 2021; Sinha et al., 2021) and preleptotene spermatocytes (Koubova et al., 2006; Huang et al., 2023) (Figure 1D). Interestingly, genes associated with upregulation of androgen signaling were identified in both mouse and rat premeiotic cells, despite the absence of functional androgen receptors in germ cells (Wang et al., 2009). Such findings suggest an overlap of gene expression between androgen-driven responses in Sertoli cells and germ cell responses to Sertoli cell signaling. Meiotic cells exhibited a dramatic metabolic switch resulting in upregulation of anaerobic glycolysis and gluconeogenesis in the late stages of spermatogenesis. Numerous long non-coding RNAs (lncRNAs) were expressed in germ cells at various stages. To compare lncRNA expression between species, we took mouse annotations of lncRNAs and inferred orthologous rat loci via a genomic liftover. While the total number of lncRNAs is more consistent between cell types than total mRNA, the proportion of shared lncRNAs does reduce as spermatogenesis proceeds, particularly in elongating spermatids, similar to total mRNA (Figure 3B; Table S2). Of the lncRNAs expressed in both rat and mouse, multiple lncRNAs show exceedingly similar gene expression patterns (Figure 3C). *Gas5* is expressed in undifferentiated spermatogonia of both species, while *Tsirn1* (mouse)/*LOC102546822* (rat) is specifically expressed in late-differentiating spermatogonia. A number of lncRNAs show mid-to-late meiotic expression such as *Gm10069* (mouse)/*LOC102554670* (rat) and *Rbm46os* (mouse)/*LOC102555909* (rat). Still, more show specific expression in round spermatids such as *A930024E05Rik* (mouse)/*Lrap* (rat), and others show high expression in elongating spermatids, for example *1700027A15Rik* (mouse, also known as *Hsf2*)/*LOC102548134* (rat) suggesting an important role in spermiogenesis given the RNA’s retention after transcription has ceased (Hong et al., 2021).

### Transcriptomic differences between mouse and rat germ cells

To examine differences in spermatogenesis between the species, we identified differentially expressed genes between mice and rat within each cell type separately (Figures S5A and S5B; Table S3). *Etv4*, *Cd9*, and *Crabp1* were comparatively elevated in rat SSCs, whereas *Gfra1*, *Foxp1*, and *Zbtb16* were expressed higher in mouse. *Dmrt1* was more expressed in mouse differentiating spermatogonia, but *Sycp3* was expressed higher in rat spermatocytes. *Fgf14* was expressed higher in mouse spermatocytes, and *Spem2* showed elevated expression in mouse spermatids, whereas *Spam1* and the transition proteins *Tnp1* and *Tnp2* were expressed earlier in rat spermatids than rat. *Prm1* expression in spermatids was higher in the rat than mouse, whereas the inverse is true for *Prm2*, a result that is in accordance with the observation that protamine 2 makes up 67% of mouse protamines but only 2%–5% in rat (Corzett et al., 2002).

**Figure 4:**
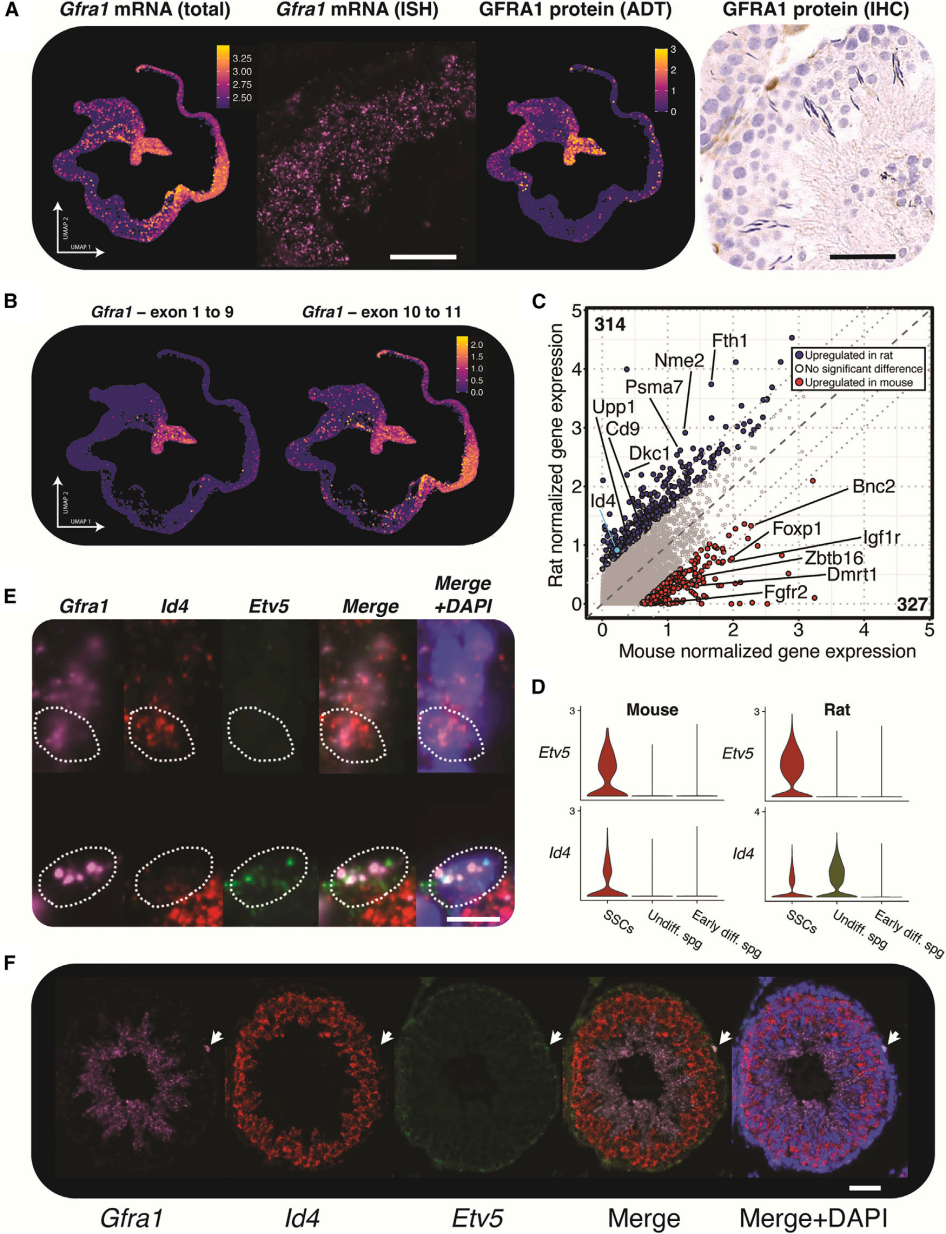
*Id4* and *Etv5* mark distinct populations of spermatogonia in rat (A) From left to right: *Gfra1* mRNA distribution in rat single-cell germ cell data (*n* = 13), *Gfra1* mRNA localization in rat testis histology via ISH (representative image from 3 replicates; scale bar, 50 μm), GFRA1 protein distribution in rat single-cell data (*n* = 5), GFRA1 protein localization in rat testis histology via IHC (representative image from 3 replicates; scale bar, 50 μm). (B) *Gfra1* expression counting transcripts that match individual exons (*n* = 9). (C) Differential gene expression between mice and rats for the undifferentiated spermatogonia across all integrated samples. Significantly different genes, including *Id4*, are colored. (D) Expression of *Etv5* and *Id4* in the early stages of mouse and rat germ cell differentiation. (E) *Gfra1*^+^*Etv5*^+^*Id4*^−^ and *Gfra1*^+^*Etv5*^−^*Id4*^+^ cells were observed on the basement membranes of rat seminiferous tubules visualized by *in situ* RNA hybridization. Dotted lines indicate estimate of cell boundaries, representative of 3 replicates. Scale bar, 10 μm. (F) Representative tubule; scale bar, 50 μm. *Gfra1*+ *Etv5*+ *Id4−* cell denoted by white arrow.

To examine transcription factor (TF) expression in SSCs, we first sought to unambiguously identify undifferentiated spermatogonia. *Gfra1* encodes a GDNF receptor critical for retaining stem cell function in SSCs and is a known marker for undifferentiated spermatogonia (Grasso et al., 2012). In the rat, both undifferentiated spermatogonia and early spermatids expressed *Gfra1* mRNA, confirmed in histological sections by *in situ* RNA hybridization, but protein expression was limited to the undifferentiated spermatogonia as measured by CITE-seq and immunohistochemistry (Figure 4A). This discrepancy may be accounted for by the fact that only exons 10 and 11 of *Gfra1* are detected in spermatids (Figure 4B), suggesting that a new *Gfra1* splice variant may account for why some *Gfra1* transcripts are detected in spermiogenesis, and suggests an alternative function of *Gfra1* in late spermatogenesis.

We identified 534 differentially expressed genes between mouse and rat undifferentiated spermatogonia including *Id4*, *Foxp1*, *Igf1r*, *Dmrt1*, and *Upp1* (Figure 4C). As *Id4* is a key marker of SSCs in the mouse (Helsel et al., 2017), it was surprising to see little expression in the rat SSCs, so we selected this gene for further analysis along with the TF *Etv5* (which did not show differential expression between species and shows co-expression with *Gfra1* in the SSC cluster, Figure S5C). *Id4* and *Etv5* co-localized in our mouse dataset, but in the rat sc/snRNA-seq, *Etv5* and *Id4* transcripts mark distinct populations of cells (Figure 4D), suggesting that in the rat, there exist two populations of cells within the undifferentiated spermatogonia, one *Gfra1*^+^*Id4*^+^*Etv5*^−^ population and another *Gfra1*^+^*Id4*^−^*Etv5*^+^ population. Supporting this, cells matching these criteria were found along the basement membrane of tubules by *in situ* RNA hybridization in the rat (Figures 4E and 4F). Therefore, *Id4* has been shown to be chiefly expressed in SSCs in mouse, which is not conserved in rat, suggesting that *Id4* may have diverging functions even among closely related species.

### Chromatin accessibility dynamics throughout spermatogenesis

**Figure 5:**
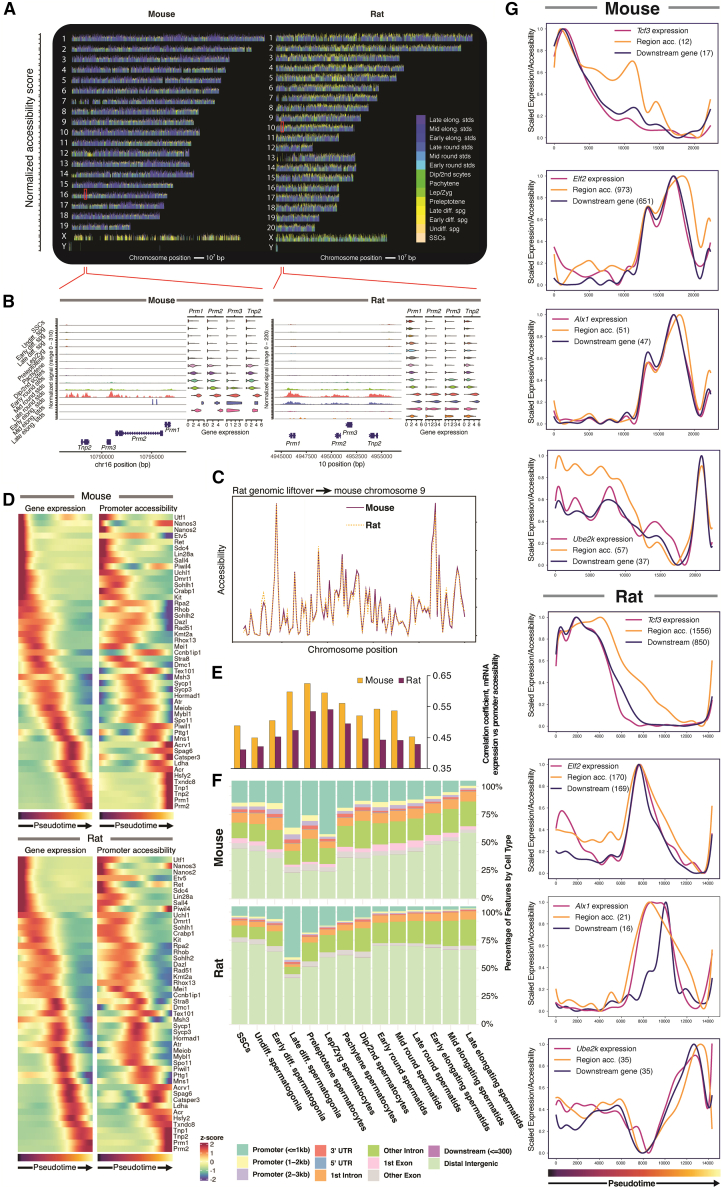
Chromatin accessibility in rodent spermatogenesis (A) Differentially expressed peaks specific to individual cell types across integrated ATAC datasets (mouse *n* = 6, rat *n* = 4 in all panels in this figure). Peaks are arranged along each chromosome on the x axis, with the height of each peak proportional to the log-fold change in peak accessibility. (B) Accessibility of chromatin for regions corresponding to red boxes in (A) that contain conserved genes associated with histone replacement. Gene expression for each gene is shown as violin plots. (C) Gene expression for select marker genes with matching ATAC promoter activity score in both species along pseudotime trajectory. (D) Broad-scale comparison of mouse chromosome 9 displaying average accessibility for binned genomic locations along with corresponding rat locations using genomic liftover. (E) Pearson’s correlation of gene expression and promoter activity was assessed by cell type. (F) Breakdown of differentially expressed peak genomic locations by cell type and species. (G) Pseudotime-ordered activity of four representative transcription factors active at different stages of spermatogenesis. For each TF, gene expression is shown along with accessibility of target regions and downstream gene expression aggregate. The number of associated chromatin peaks or genes in the corresponding regulon is shown in parentheses.

Male germ cell maturation is accompanied by changes in chromatin accessibility (Figure 5A). Some chromatin regions, such as those containing the histone replacement protein genes *Tnp2* and *Prm1-3*, undergo dramatic remodeling across cell types and exhibit high accessibility in late round and early elongating spermatids, associated with intense transcription (Figure 5B). On a larger scale, through a comparison of chromatin structure via a genomic liftover of orthologous sequences, we found that epigenetic features are remarkably conserved between the species (Figures 5C and S6A).

We next assessed the concordance between chromatin accessibility and gene expression. We selected genes known to be important at various stages of germ cell development for both species and calculated the chromatin accessibility at gene body and promoter regions (±2 kb from the transcription start site) for each gene along its pseudotime trajectory (Figure 5D). In each case, an increase in promoter accessibility corresponded with increased gene expression. Examining the correlation of transcription and chromatin accessibility of all genes between cell types revealed that correlations are comparatively low in SSCs and spermatogonia, rising to the highest levels in early meiosis and then dropping again once the cells enter spermiogenesis (Figures 5E and S5B). Then, examining the locations of differentially accessible peaks revealed a substantial relative increase in the proportion of promoter-associated peaks in the late-differentiating spermatogonia in both species that continues until mid-meiosis (Figure 5F). After meiosis, this proportion reduced, and most differentially accessible peaks were found in intergenic and intronic sequences. Taken together, we find dramatic shifts in chromatin accessibility at the onset of meiosis together with a greater correlation of promoter accessibility to gene expression, suggesting tight epigenetic control of transcription of meiotic genes that begins in the differentiating spermatogonia.

### Gene regulatory network analysis reveals conserved mechanisms of transcriptional control

To further understand the mechanistic basis for spermatogenesis, we jointly explored TF interactions with chromatin and downstream gene expression. TF expression and motif accessibility displayed correlations across the breadth of cell types (Figure S5C). Using SCENIC+ (Bravo González-Blas et al., 2023) with our multiomic snRNA/ATAC-seq data, we inferred links between predicted enhancer and promoter regions along with predicted TF-gene target links within single cells. This analysis yielded a collection of enhancer-gene regulatory networks (GRNs) that integrates region co-accessibility, TF motifs, and gene expression data to yield discrete “regulons.” After filtering to retain high-confidence regulons, we identified 174 distinct regulons in the mouse (113 activating regulators and 62 repressive regulators) along with 318 regulons in the rat (220 positive regulators and 99 repressive regulators). For each TF, there was a mean of 481.6 and 352.9 regions per regulon and 175.7 and 211.6 downstream genes per regulon for mouse and rat, respectively (Figures S7A and S7B; Table S4). Most positively regulated regulons show tight associations between TF expression, target region accessibility, and downstream gene expression (Figure 5G). We found that 40 regulons were shared between mouse and rat, and the majority showed strong conservation between mouse and rat when cells were ordered by pseudotime. TCF3 was activated early in spermatogenesis. In contrast, the ELF2 regulon was activated during meiosis, although in the mouse, this persisted into spermiogenesis, whereas in the rat, it showed a more pronounced decrease in activity after meiosis (Figure 5G). The ALX1 regulon showed similar late-meiotic and spermiogenic activation, although we observed a delay in gene upregulation in the rat. The UBE2K regulation also showed a similar pattern in mice and rats: early moderate activation, near-complete drop during meiosis, and then a sharp peak of TF expression, target peaks, and downstream gene expression in late round spermatids (Figure 5G). Overall, we saw strong correlations of TF expression with target region accessibility and downstream gene expression for a wide selection of regulons, underscoring the importance of tight timing of the effects of these TFs for proper progression of spermatogenesis.

### *Cd9* RNA and protein expression correlates with putative enhancer elements

**Figure 6:**
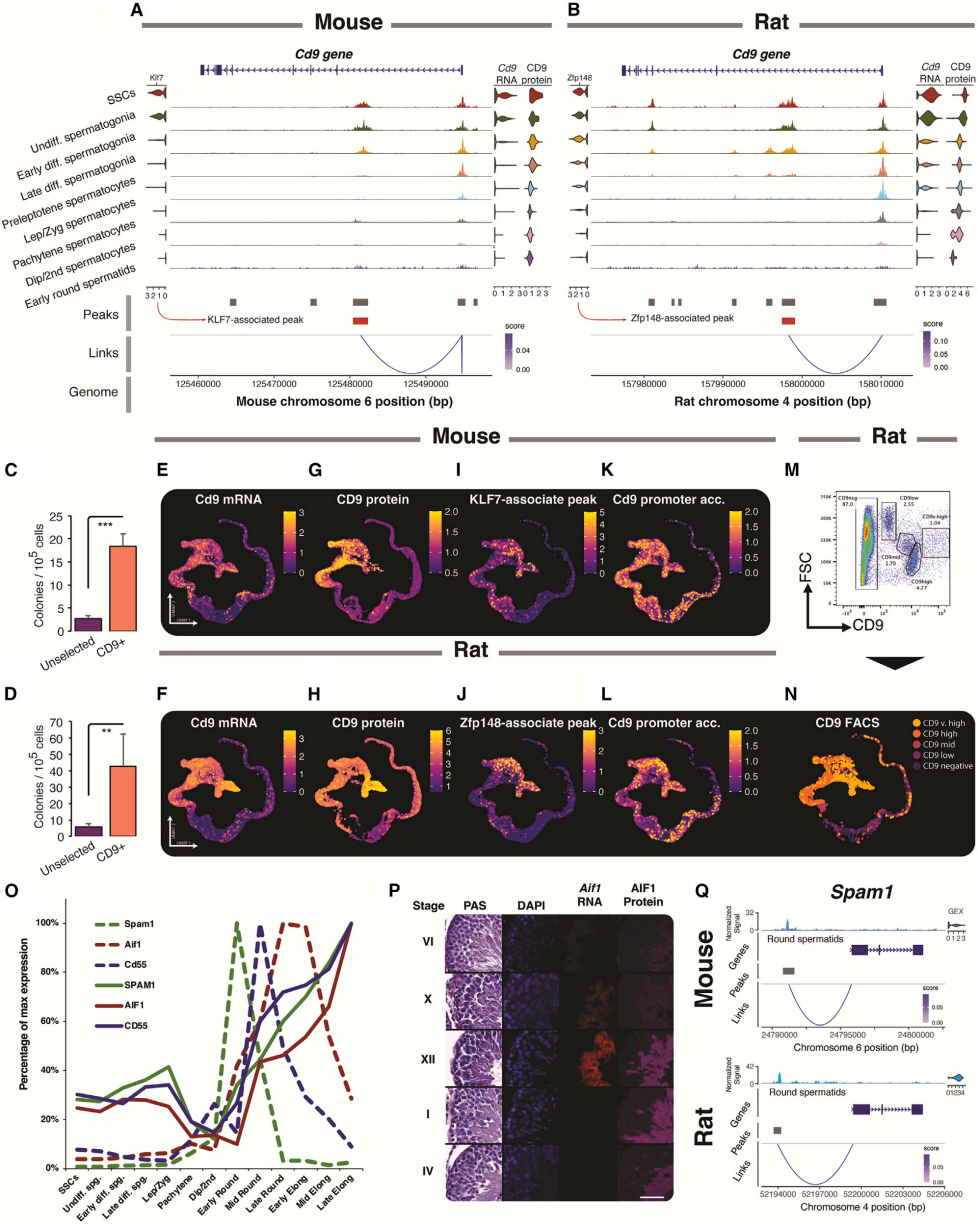
Chromatin, gene, and protein relationships (A and B) (A) Mouse and (B) rat *Cd9* chromatin accessibility by cell type, together with *Cd9* gene expression and CD9 protein as assessed by ADT (*n* = 2 mice, *n* = 2 rats). Transcription factors *Klf7* and *Zfp148* are predicted by GRN to associate with *Cd9* in mice and rats, respectively. (C) Transplant efficiency of CD9^+^ mouse cells adapted from Kanatsu-Shinohara et al., 2004; *n* = 16 CD9^+^ and *n* = 13 control. (D) Transplant efficiency of CD9^+^ rat cells by MACS selection, significance assessed by Student’s t test, ^∗∗^*p* < 0.01, ^∗∗∗^*p* < 0.001, *n* = 4. (E and F) UMAP projection of *Cd9* mRNA expression in mouse (E, *n* = 8) and rat (F, *n* = 8). (G and H) CD9 protein expression in mouse (G, *n* = 2) and rat (H, *n* = 2). (I and J) Downstream predicted enhancer accessibility in mouse (I, *n* = 6) and rat (J, *n* = 4). (K and L) *Cd9* promoter accessibility in mouse (K, *n* = 6) and rat (L, *n* = 4). (M) Flow cytometric analysis of CD9 expression and gates used to sort cells. (N) Cells sorted in (M) were encapsulated and projected onto the UMAP space (*n* = 1). (O) mRNA and protein expression for three select spermatid-associated genes normalized by percentage of max expression (mRNA, *n* = 13; CD55, *n* = 6; SPAM1, *n* = 2; AIF1, *n* = 3). (P) Histology of PAS-stained staged sections with DAPI, *Aif1* ISH, and AIF1 immunofluorescence; scale bar = 50 μm, representative image of 3 replicates shown. (Q) *Spam1* expression is associated with a peak upstream of the transcriptional start site in both species matched by liftover (*n* = 4).

To investigate the interplay between accessibility, transcription, and protein expression, we analyzed CD9 protein surface expression as assayed by CITE-seq, selecting CD9 as this protein is enriched on SSCs in mice and rats (Kanatsu-Shinohara et al., 2004). We found that the promoter of *Cd9* was widely accessible in pre-meiotic cells in both species (Figures 6A and 6B). This tracks broadly with mRNA and protein expression via our single-cell sequencing data. However, mRNA and protein expression were markedly higher in stem cells than in undifferentiated spermatogonia. *Cd9* expression in functional stem cells was confirmed in both species via a transplantation assay (Figures 6C and 6D). Promoter accessibility was reasonably broad in premeiotic cells and did not correlate well with gene/protein expression. Both species display an accessibility peak in the first intron of *Cd9* that significantly linked to mRNA expression, suggesting a conserved regulatory element. Additionally, in the rat, but not mouse, the presence of this peak coupled with the lack of a nearby putative repressor element peak correlated with the highest mRNA/protein expression, suggesting localization of a distal enhancer element. The downstream peak correlated more strongly with mRNA and protein expression than did the promoter region (Figures 6E–6L). *Cd9* expression was therefore better predicted by nearby putative enhancer regions than the promoter, which was broadly open in premeiotic cells.

Given the markedly higher protein expression in the stem cell compartment of the rat using CITE-seq, we next sought to confirm this finding by flow cytometry. We identified five populations of rat testicular cells using CD9 antibody staining (Figure 6M), which were then sorted based upon CD9 protein abundance via fluorescence-activated cell sorting. Each population was subjected to scRNA-seq and mapped onto the rat uniform manifold approximation and projection (UMAP) (Figure 6N). As predicted, high CD9-expressing cells mapped to undifferentiated spermatogonia, whereas CD9-negative cells were chiefly meiotic and post-meiotic cells. This result confirmed that spermatogonial populations can be sorted based on CD9 expression level alone.

We also investigated proteins involved in post-meiotic haploid germ cells in the rat, CD55, AIF1, and SPAM1. We saw evidence for delayed translation in all three, with peak mRNA expression preceding maximum protein detection (Figure 6O). For AIF1, we confirmed this via RNA *in situ* hybridization and immunofluorescence, indicating that mRNA was detected in stage X up to stage XII whereas protein was detected from stage XII elongating spermatids onward (Figure 6P), staging consistent with our single-cell observations.

Examination of peaks of accessible chromatin concomitant with gene expression within individual cells can provide evidence of sites occupied by DNA-binding proteins, which may represent key regulatory regions, especially when conserved across species. Simultaneous profiling of RNA and ATAC datasets revealed peaks, both proximal and distal to transcriptional start sites, that correlated tightly with gene expression across a range of genes that were conserved between mouse and rat (Figure S7C). For example, *Sdc4* showed a distal element downstream of the promoter site, whereas the meiotic gene *Sycp3* displayed a proximal element close to the promoter that correlated with gene expression. Other genes such as *Spam1*, expressed at the onset of spermiogenesis, indicated a strong relationship of gene expression with upstream elements (Figure 6Q). These elements are observed in both mouse and rat, consistent with their conserved regulatory roles.

### Gene regulatory analysis provides a cohesive view of the conserved regulation of spermatogenesis

**Figure 7:**
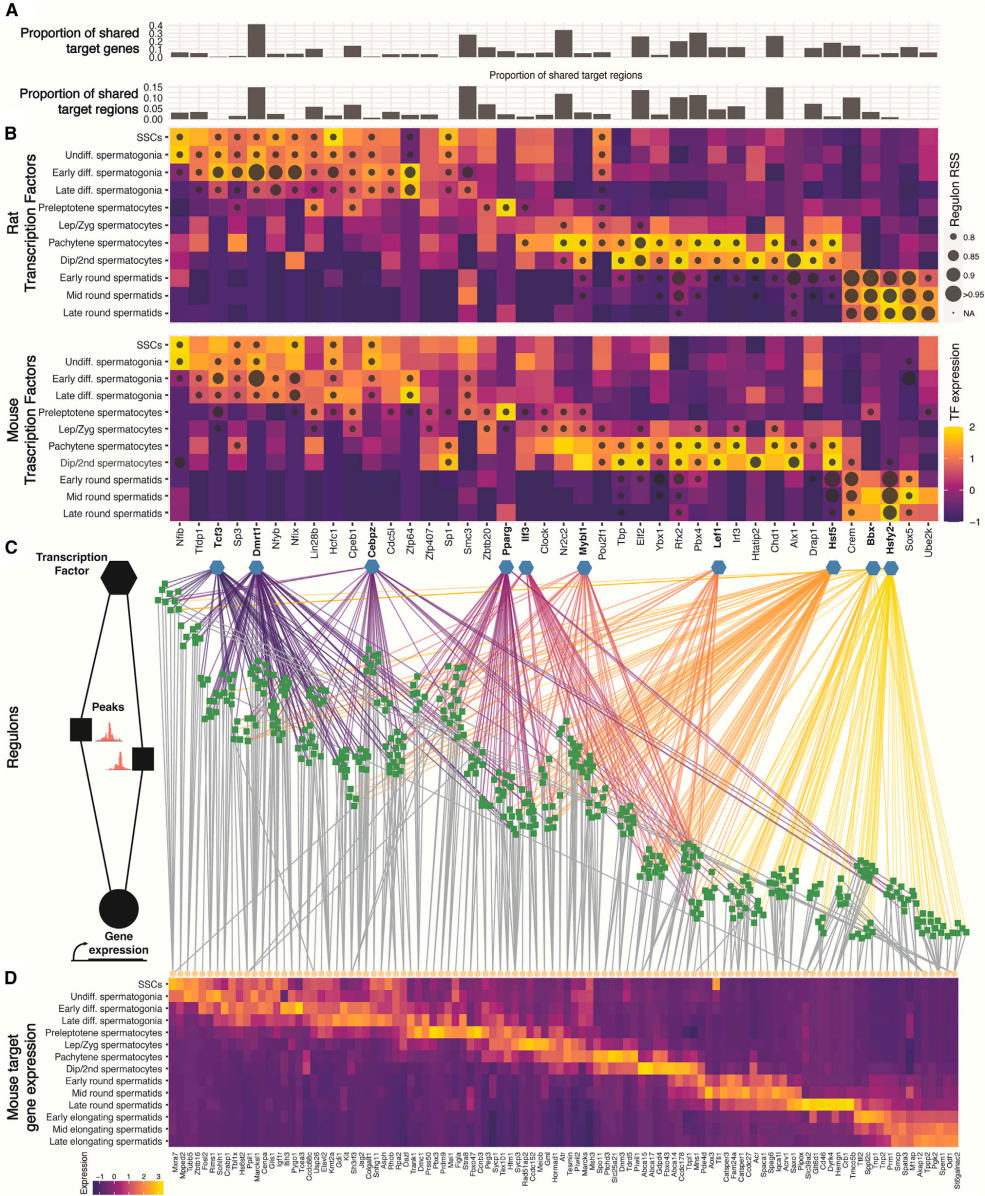
Gene regulatory analysis of rodent spermatogenesis (A) For each regulon, the proportion of shared target genes and target chromosomal regions are shown. (B) Heatmap of gene expression for 40 regulons significantly associated with spermatogenesis in both mouse and rat. Color shows gene expression of the TF by cell type, dot size indicates regulon activation as indicated by regulon specificity score. (C) For 10 selected regulons, gene regulatory network nodes are shown. Blue hexagons indicate selected TFs. Each TF’s connections to target regions have been colored with a different color. Target regions are indicated as square boxes. Connections between regions and target genes (yellow circles) are indicated in gray. A subset of target regions and genes are shown, selected by taking the top 10 gene hits for each regulon in addition to any genes from Figure 2B that were found in the selected regulons. (D) Heatmap of the gene expression of the selected genes shown by cell type. All data in this figure were generated from snRNA/ATAC-seq multiomic profiling (*n* = 6 mice and 4 rats).

When examining the conserved transcriptional regulons in mice and rats, several trends are apparent. Of the 40 conserved regulons in our analysis, there is appreciable variation in the proportion of conserved downstream regions and genes (Figure 7A). Interestingly, both TF expression and their respective regulon activation were highly conserved by cell type between the species with few exceptions (Figure 7B), suggesting that while the activity of the TFs is conserved, the downstream targets may be species specific. We selected 10 regulons active at different stages of spermatogenesis and visualized their top gene target and accessible regions (Figure 7C). Even with a limited selection of TFs and downstream genes/regions, a complex progression of peak accessibility and subsequent gene expression is brought into focus. While some far-ranging connections were made, largely TF-associated peak accessibility and corresponding gene expression were linked to TFs in a cell-type-specific manner, indicating that these TFs and their regulons have limited and specific action in their respective cell types (Figure 7D). These associations can also be translated into categorizations of TF activity (Figure S7C). For example, we observed TF control of key germ cell markers such as *Sohlh1*, *Igf1r*, *Kit*, *Dmc1*, *Sycp1*, *Tex101, Spaca1*, *Acrv1*, *Tnp1*, and *Prm1*, indicating that these TFs may be master regulators of normal spermatogenic differentiation. In addition, genes with no known testis function such as *Mxra7*, *Snhg11*, *Pipox*, and *Ccdc27* were indicated, suggesting potential roles for these previously unreported spermiogenic genes.

## Discussion

Spermatogenesis is defined by three stages that are conserved across mammals: the mitotic phase, where spermatogonia divide and proliferate; meiosis, during which the critical stages of chromosome crossing-over and segregation into haploid gametes occur; and finally spermiogenesis, where spermatids differentiate into functional sperm. In this study, we characterized these events at a molecular level by simultaneously comparing chromatin accessibility across the genome with transcriptomic information for individual cells and surface marker expression of a select subset of proteins. The joint RNA/ATAC multiomic analysis allowed for the identification of GRNs, in which TFs were linked via acting chromatin regions to downstream genes. Our analysis associated cell types with distinct cellular pathways. SSCs were marked by GDNF signaling, stem cell pluripotency, and MAPK signaling, consistent with established reports (Meng et al., 2000; Niu et al., 2017; Yang et al., 2021). In addition, in concordance with published data, we observed an upregulation of oxidative phosphorylation in mitotic phases (Chen et al., 2020), but not stem cells (Helsel et al., 2017), followed by a switch to glycolysis in spermiogenesis (Boussouar and Benahmed 2004), indicative of the dramatic bioenergetic shifts throughout spermatogenesis. We saw evidence for netrin signaling being important in late meiosis/early spermiogenesis, as opposed to its importance in spermatogonial differentiation that has been reported (Barroca et al., 2022). The evidence for netrin signaling’s importance in late meiosis and early spermiogenesis offers a new perspective and may suggest a broader role beyond its established function in spermatogonial differentiation, potentially in guiding cellular transitions and structural organization during sperm development and influencing chromatin remodeling or cytoskeletal dynamics in late spermatogenic phases (Lai Wing Sun et al., 2011).

Mice and rats displayed remarkably similar patterns of chromatin accessibility. Notably, the correlation of gene expression with promoter accessibility was strongest during meiosis in both species, suggesting a stringent control of chromatin accessibility during meiosis. Chromatin accessibility could increase either directly as a control of expression or as a side effect of the dramatic chromatin changes during meiosis. The most substantial changes in germ cell gene accessibility were those accompanying the entry into meiosis, potentially because chromatin accessibility may control the frequency of double-stranded breaks during prophase I (Liu et al., 2022).

Our comparative multiomic analysis of mouse and rat spermiogenesis revealed a number of strong transcriptional, regulatory and chromatin similarities between mice and rats across the complex differentiation process to make male gametes. These similarities were strongest in premeiotic and meiotic cells, whereas spermatids exhibit more differences in gene and lncRNA expression, consistent with the sperm morphological differences between the two species. lncRNAs are known to have important roles in spermatogenesis. We detected stage-specific and conserved expression of known lncRNAs involved in spermatogenesis such as *Hsf2*, expressed in elongating spermatids, whose absence is associated with abnormal sperm morphology and reduced fertility and a female-biased sex ratio in offspring (Hong et al., 2021). Gene regulatory systems were also strongly conserved, from TF abundance to regulon activation in cell types, although the degree of similarity varied within regulons. Such findings suggest that despite being expressed in the same cell types, the principal downstream TF targets can be species-specific depending. While TCF3 is known as a stem cell regulatory factor (Zhou et al., 2021), our GRN analysis revealed that its effects extend into differentiating spermatogonia. TCF3 did indeed associate with a series of peaks linked to specifically stem-cell-expressed genes; other modules of downstream chromatin/gene associations were expressed in late-differentiating spermatogonia, such as TCEA3, whose function in spermatogenesis has yet to be determined. We found that *Pparg* had highly specific expression and downstream effects, limited to preleptotene spermatocytes. PPARγ forms heterodimers with the retinoid X receptor and is a crucial protein for proper spermatogenesis (Santoro et al., 2020). Our network highlighted other key TFs, such as MYB11, a master regulator of meiosis, which has been associated with a range of enhancer elements primed prior to meiosis. We found a cluster of preleptotene MYB11 enhancers as well as those acting in later spermatocytes.

We also observed notable differences in expression and regulatory patterns between species, including *Zbtb16*, which is essential for SSC maintenance in mice (Buaas et al., 2004), but whose expression was much reduced in rats relative to mice in our comparison. This raises the question as to whether rat SSCs maintain stemness with less ZBTB16 protein, or if post-transcriptional mechanisms compensate, or if interactions with another protein such as SALL4 explains the difference (Lovelace et al., 2016)*.* For example, high expression of the TF ID4 marks SSCs in mice (Helsel et al., 2017), and the TF ETV5 is necessary for pro-stem cell signaling in SSCs (Oatley et al., 2006; Yang et al., 2021) as its knockout gradually results in a Sertoli-cell-only phenotype (Morrow et al., 2007; Schlesser et al., 2008). *Id4* and *Etv5* show a very strong overlap (Hermann et al., 2018) in single-cell profiles of the mouse testis, consistent with both being markers of SSCs. However, undifferentiated spermatogonia in the rat consistently cluster into two clusters at the beginning of spermatogenesis; one cluster is marked distinctly by *Etv5* and another by *Id4.* Therefore, we conclude that, unlike in mouse, rat SSCs are not characterized by high *Id4* expression.

CD9 is a tetraspanin membrane protein that is involved in a wide range of physiological processes, including cell motility and fertilization (Hemler 2005), and is expressed in mouse and rat SSCs (Kanatsu-Shinohara et al., 2004; Hermann et al., 2018). While there is evidence that CD9 can serve as a marker of human SSCs (Zohni et al., 2012) and undifferentiated spermatogonia in goats (Kaul and Kumari, 2012), its expression is not exclusively limited to SSCs. Here, we showed that levels of CD9 protein cell surface expression varied by cell type in both rat and mice. Moreover, germ cells sorted by CD9 surface expression accurately mapped back to our atlas, indicating that CD9 protein expression alone can identify certain spermatogenic cell types.

Finally, evolutionarily conserved genes are typically involved in key biologically processes. On this basis, the conserved 40 regulons identified by our analysis are likely to play a pivotal role in spermatogenesis, ensuring a physiological progression through each maturation stage. Furthermore, lower expression or loss of function would be anticipated to result in impaired spermatogenesis and male infertility, although this will require further experimental validation. However, previous work has demonstrated that TCF3 deficiency is associated with spermatogenesis failure in infertile human patients (Zhou et al., 2021). Similarly, DMRT1 loss of function has been demonstrated in infertile men (Zarkower and Murphy 2022) while PPARG plays a role in testis fatty acid dysmetabolism in men with impaired spermatogenesis (Olia Bagheri et al., 2021). These findings strongly support the hypothesis that our core of conserved regulons includes essential genes for the preservation of normal spermatogenesis. As such, they represent candidates for the establishment of diagnostic molecular tests as well as targeted strategies for the treatment of male infertility.

## Methods

### Animal use

All animal protocols were approved by University of Pennsylvania Institutional Animal Care and Use Committee (protocol number 800375).

### Tissue isolation

Rat cells were isolated from the testes of 3- to 4-month-old Sprague-Dawley rats transgenic for the LacZ gene under the metallothionein promoter (Clouthier et al., 1996). Mouse cells were isolated from C57BL/6 inbred mice also 3–4 months old. Tissue was chopped into fine pieces and incubated in collagenase (Sigma) at a concentration of 1 mg/mL in HBSS (Gibco) for 15 min at 37°C. Cells were spun down for 1 min at 600 *rcf*, then resuspended in warm Trypsin (Gibco, 0.25%) with 20% DNase solution (Sigma, 7 mg/mL dissolved in HBSS). Tissue was pipetted for 2 min with a 10 mL pipette and incubated at 37°C for 5 min. Then tissue was pipetted for another 2 min and incubated at 37°C for 3 min. Fetal bovine serum (FBS) (Sigma F2442) was added to stop the digestion. Additional DNase was added until no turbidity was visible. Cells were washed in PBS-S twice (PBS [Gibco] with 1% FBS, 10 mM HEPES [Sigma-Aldrich], 1 mg/mL glucose [Sigma-Aldrich], 1 mM pyruvate [Gibco], 50 units/mL penicillin [Gibco], and 50 μg/mL streptomycin [Gibco] prepared as described in the study by Kubota and Brinster (2008)). All centrifugation steps were 5 min at 600 *rcf*.

Cells were processed both as unselected samples and enriched for EpCAM as described further. Each biological replicate was then used for either encapsulation of cells with antibody treatment or further processed as nuclei for GEX+ATAC assay.

### Single-cell multiomic sequencing

Cells were encapsulated and libraries generated for CITE-seq using a Chromium Next GEM Single Cells 3′ Kit v.3.1 with Feature Barcoding (10X Genomics) per manufacturer’s protocol. Prepared nuclei were encapsulated with the Chromium Next GEM Single-Cell Multiome ATAC + Gene Expression kit following manufacturer’s instructions. Each biological replicate was encapsulated individually. Libraries were sequenced on a NextSeq2000 sequencer (Illumina) using a 100-cycle sequencing kits to a minimum depth of 30k reads per cell. CellRanger v.7.0.0 was used to align reads to the *Mus musculus* reference (GENCODE vM23/Ensembl 98) and the *Rattus norvegicus* 6.0 DNA primary assembly along with the corresponding GTF file (v.6.0.85) from Ensembl (Yates et al., 2020).

### Single-cell multiomic data processing and integration

Gene counts were analyzed with Seurat v.4 (Butler et al., 2018) for clustering, integration, and differential gene expression and Monocle version 3 (Qiu et al., 2017) for clustering and pseudotime. Filtering cutoffs were set as follows: minimum genes per cell = 500, maximum genes = 5,000, and minimum unique molecular identifiers (UMIs) per cell were set on a per-sample basis after inspecting a rank plot of UMI/cell. For cells with ATAC data, filtering criteria of minimum fragments per cell of 100 and maximum of 10,000 were also applied. Cells with over 20% mitochondrial reads were also excluded. The RNA assay was processed using Seurat’s NormalizeData and ScaleData using 2,000 variable features before being integrated using Seurat’s FindIntegrationAnchors and IntegrateData functions with default parameters. The first 30 dimensions were used for integration of the RNA assay of all samples (regardless of associated modality) and joint UMAP creation. Mouse and rat samples were integrated as mentioned earlier, ensuring all variable features were matched by name to homologous genes. Clusters were found using Monocle (cluster_cells, resolution = 5 × 10^−5^, k = 8, partition_qval = 0.05). Differentially expressed genes were identified via Seurat’s FindMarkers function with default parameters. Pseudotime was generated using Monocle’s learn_graph (default parameters). TE element analysis used SoloTE (Rodríguez-Quiroz and Valdebenito-Maturana 2022) on the RNA assay of mouse and rat datasets using default parameters and normalized using Seurat’s default normalization method on a combined assay involving all genes and TEs.

For the scRNA-seq + ATAC-seq samples, the ATAC modality was processed independently of the RNA assay. Peaks were called using Signac (Stuart et al., 2021) with MACS2, and gene activity scores were calculated with Signac’s GeneActivity function. First, a shared peak list was created by merging all samples and then that shared peak list was used to create Seurat objects for each sample individually. FindTopFeatures with min.cutoff = 10, RunTFIDF, and RunSVD were run. Samples were integrated using FindIntegrationAnchors, reduction = “rlsi,” and 2:30 dimensions. Then LSI embeddings were integrated using 1:30 dimensions. Finally, the integrated ATAC assay was joined to the integrated RNA assay by cell name. For linking peaks to genes, Signac’s LinkPeaks function was used with default parameters to link ATAC and RNA assays.

To compare nascent versus all mRNA, spliced, unspliced, and ambiguous counts were determined using Velocyto (La et al., 2018). Then, using only unspliced counts, the whole process of integration and cell type assignments and UMAP projection was performed as detailed earlier, after which the correlation of original and unspliced-only cell type designations was correlated by cell types.

For CD9-sorted cells, each sorted fraction was included in the main integration with all other cells and their identities were retained. The CD9-sorted cells were subset and projected onto the same UMAP.

## Resource availability

### Lead contact

Requests for further information and resources should be directed to and will be fulfilled by the lead contact, Eoin C. Whelan (ewhelan@vet.upenn.edu).

### Materials availability

This study did not generate new unique reagents.

### Data and code availability

Single-cell RNA/ADT/ATAC data have been deposited at NCBI GEO at GEO accession number GSE268104 and are publicly available as of the date of publication. All original code is available in this paper’s supplemental information.

## Acknowledgments

We thank Kotaro Sasaki, Keren Cheng, Leslie King, and Andrew Modzelewski for helpful advice. We also thank C. Freeman, R. Naroznowski, and D. Lee for animal maintenance. We are grateful for the support of Robert J. Kleberg, Jr and Helen C. Kleberg Foundation (RLB). https://www.klebergfoundation.org/grant-guidelines/medical-research/. Additionally, the Penn Vet Comparative Pathology Core receives funding from the Abrahamson Cancer Center Support Grant (P30 CA016520), the Aperio Versa 200 scanner used for imaging was acquired through an NIH Shared Instrumentation Grant (S10 OD023465-01A1), and the Leica BOND RXm instrument used for IHC and 10.13039/100011054ISH was acquired through the Penn Vet IIZD Core pilot grant opportunity 2022.

## Author contributions

Conceptualization, E.C.W. and R.L.B.; methodology, E.C.W., D.P.B., R.L.B., J.H.S., and D.S.; investigation, E.C.W., J.J.S., F.Y., M.R.A., A.R., C.M., E.R., J.H.S., and D.S.; visualization, E.C.W., J.H.S., and D.S.; funding acquisition, R.L.B.; project supervision, R.L.B.; writing – original draft, E.C.W.; writing – review and editing, E.C.W., F.Y., A.R., D.P.B., J.H.S., and R.L.B.

## Declaration of interests

The authors declare no competing interests.

## Supplemental information

Document S1. Figures S1–S7, Table S5, and Supplemental Methods

Table S1. Genes expressed in mouse and rat germ cells

Table S2. lncRNAs expressed in mouse and rat germ cells

Table S3. DEGs between mouse and rat germ cells

Table S4. Mouse and rat germ cell regulons

Document S2. Article plus supplemental information

## Footnotes

Supplemental information can be found online at https://doi.org/10.1016/j.stemcr.2025.102449.
