# Supplementary material for: Single-cell multiomic comparison of mouse and rat spermatogenesis reveals gene regulatory networks conserved for over 20 million years
Source: Stem Cell Reports. 2025 Mar 13;20(4):102449. doi: 10.1016/j.stemcr.2025.102449 (PMC12069898; doi:10.1016/j.stemcr.2025.102449)
Supplement: Document S1. Figures S1–S7, Table S5, and Supplemental Methods [file mmc1.pdf]

Stem Cell Reports, Volume *20*

Supplemental Information

Single-cell multiomic comparison of mouse and rat spermatogenesis reveals gene regulatory networks conserved for over 20 million years

Eoin C. Whelan, John J. Swain, Jonathan H. Sussman, David Smith, Fan Yang, Antonia Rotolo, Mary R. Avarbock, Clara Malekshahi, Enrico Radaelli, Daniel P. Beiting, and Ralph L. Brinster

SUPPLEMENTARY FIGURES

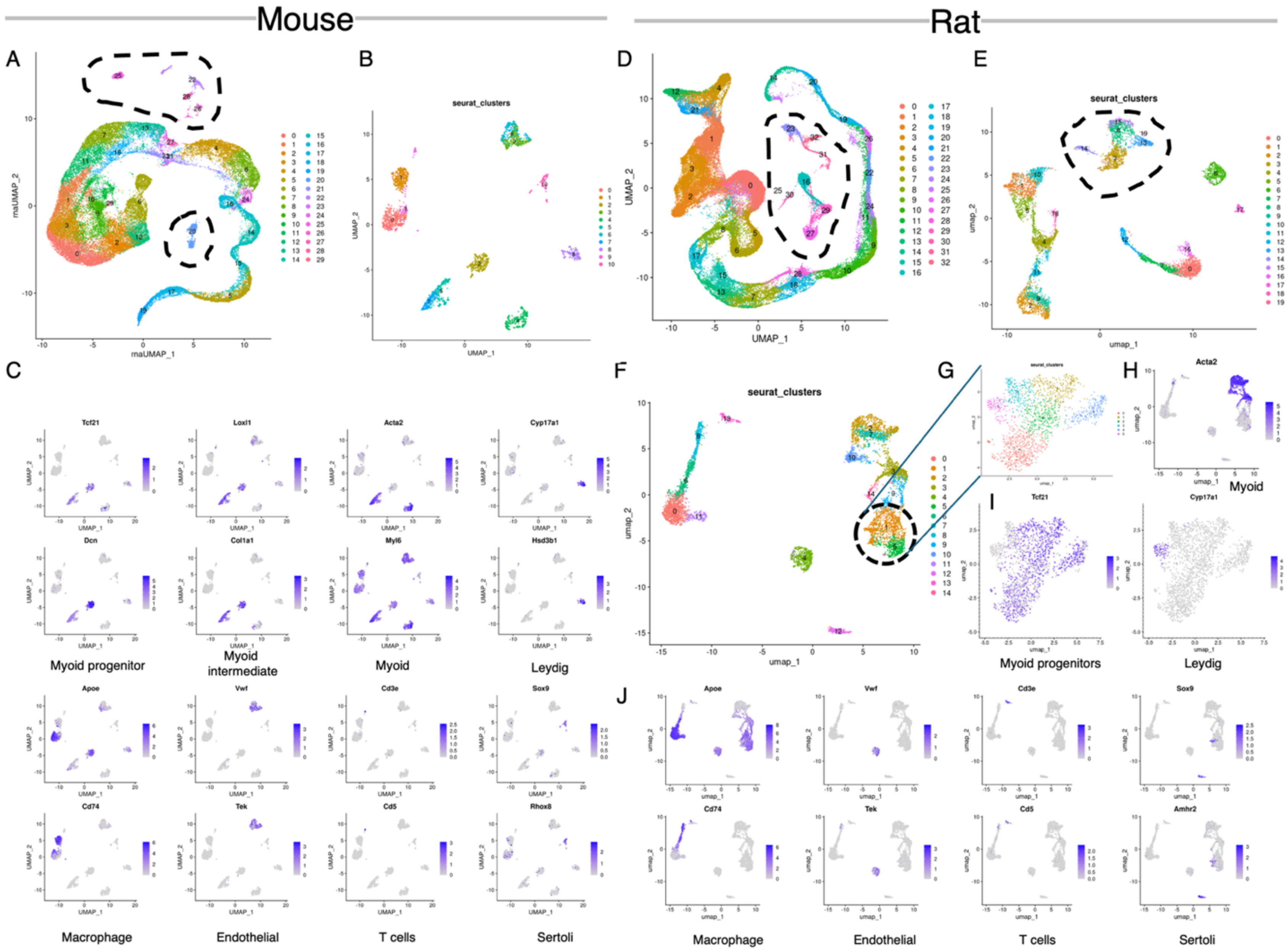
Mouse
Rat
A
B
C
D
E
F
G
H
I
J
seurat_clusters
Myoid progenitor
Myoid intermediate
Myoid
Leydig
Macrophage
Endothelial
T cells
Sertoli
Myoid progenitors
Tcf21
Loxl1
Acta2
Cyp17a1
Dcn
Col1a1
Myl6
Hsd3b1
Apoe
Vwf
Cd3e
Sox9
Cd74
Tek
Cd5
Rhox8
Amhr2

Figure S1. Somatic cells in the mouse and rat. (A) Clustering of all mouse testicular cells. Clusters within dotted lines display somatic markers and were subclustered for somatic analysis. All other cells were used in the germ cell analysis. (B) Resulting somatic clusters after subclustering. (C) *Tcf21*+ Myoid progenitors, intermediate cells and their progeny, myoid and Leydig cells were discrete clusters in mouse. Macrophages, endothelial cells, T cells and Sertoli cells were also apparent and representative gene expression is shown for each of these clusters. (D) Clustering of all rat testicular cells. Clusters within dotted lines display somatic markers and were subclustered for somatic analysis. All other cells were used in the germ cell analysis. (E) Subclustered cells retained some germ cell clusters shown in the dotted lines, which were removed and reclustered again. (F) Resulting somatic clusters after the second subclustering. Myoid progenitor cells were difficult to resolve in rat (unlike mouse) from Leydig cells. (G) After another round of subclustering of the myoid progenitors/Leydig cluster, (H) both cell types were distinct as shown by *Tcf21* (progenitors) and *Cyp17a1* (Leydig cells). (I) Myoid cells show progression from progenitors in rat. (J) Markers of immune, endothelial and Sertoli cells. All data in this figure were generated from sn/scRNA-seq (n = 8 mice and 13 rats).

4

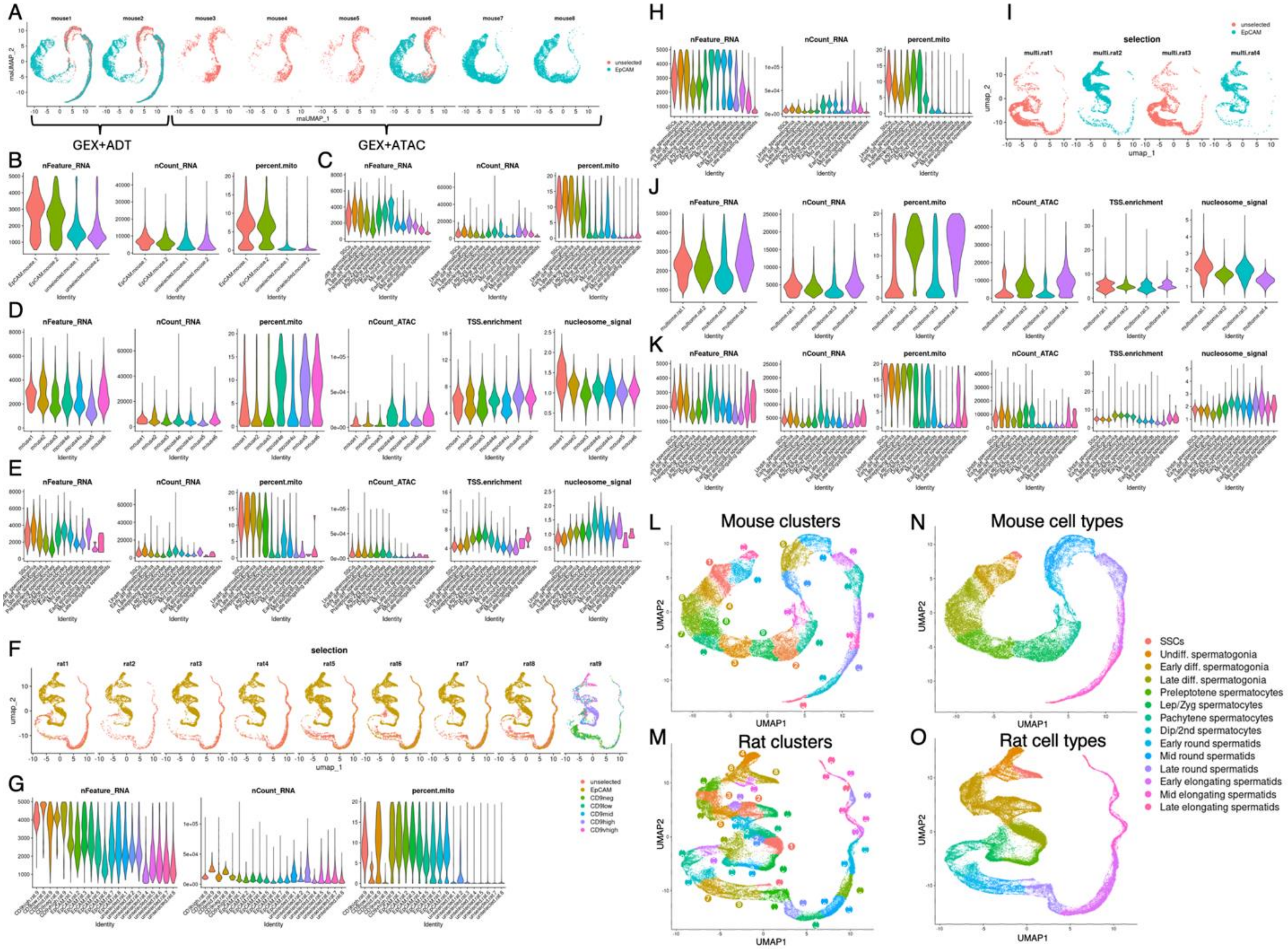

A
mouse1
mouse2
mouse3
mouse4
mouse5
mouse6
mouse7
mouse8
unselected
EpCAM
GEX+ADT
GEX+ATAC
B
C
D
E
F
G
H
I
J
K
nFeature_RNA
nCount_RNA
percent.mito
nCount_ATAC
TSS.enrichment
nucleosome_signal
Identity
selection
multi.rat1
multi.rat2
multi.rat3
multi.rat4
umap_1
umap_2
rat1
rat2
rat3
rat4
rat5
rat6
rat7
rat8
rat9
unselected
EpCAM
CD9neg
CD9low
CD9mid
CD9high
CD9vhigh
L
Mouse clusters
M
Rat clusters
N
Mouse cell types
O
Rat cell types
UMAP1
UMAP2
SSCs
Undiff. spermatogonia
Early diff. spermatogonia
Late diff. spermatogonia
Preleptotene spermatocytes
Lep/Zyg spermatocytes
Pachytene spermatocytes
Dip/2nd spermatocytes
Early round spermatids
Mid round spermatids
Late round spermatids
Early elongating spermatids
Mid elongating spermatids
Late elongating spermatids

Figure S2. Quality metrics in mouse and rat. (A) UMAP presentation of mouse biological replicates used in this study (n = 8 mice, 2 GEX+CITE and 6 GEX+ATAC). (B) Features of GEX+CITE samples. nFeature_RNA = number of genes per cell, nCount_RNA = number of unique molecular identifiers (UMIs) per cell, percent.mito = percentage of mitochondrial genes detected. (C) Features organized by cell type. (D) Features of GEX+ATAC samples by biological replicate: nCount_ATAC = number of fragments per cell, TSS.enrichment = enrichment around transcriptional start sites, nucleosome_signal = nucleosome signal. (E) Features organized by cell type. (F) UMAP presentation of rat GEX+CITE biological replicates used in this study (n = 12 rats). (G) Features of GEX+CITE samples. nFeature_RNA = number of genes per cell, nCount_RNA = number of unique molecular identifiers (UMIs) per cell, percent.mito = percentage of mitochondrial genes detected. (H) Features organized by cell type. (I) UMAP presentation of rat GEX+ATAC biological replicates (n = 4 rats). (J) Features of GEX+ATAC samples by biological replicate: nCount_ATAC = number of fragments per cell, TSS.enrichment = enrichment around transcriptional start sites, (K) Features organized by cell type. Unbiased clustering performed on mouse (L) and rat (M) datasets, combining both GEX + ATAC and GEX + ADT datasets for each species. Cell type assignments (N–O) for each species independently were based on marker gene expression.

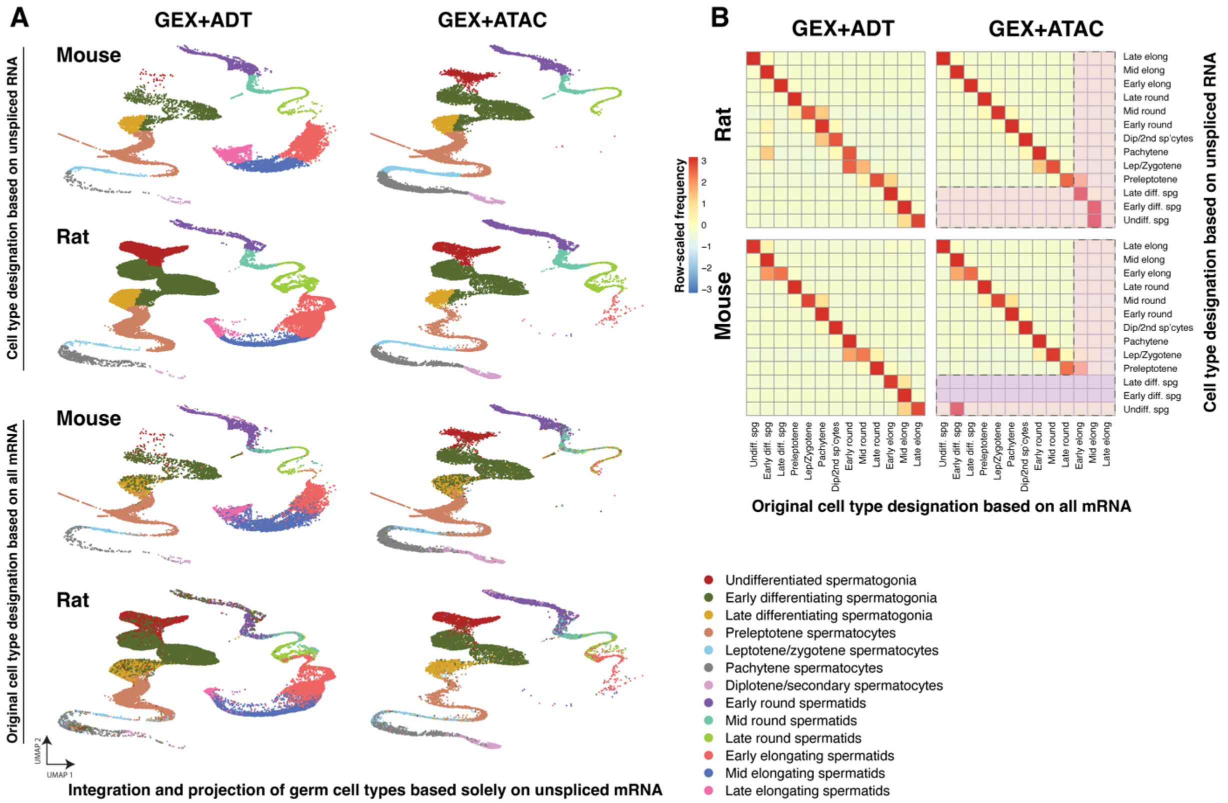

Figure S3: clustering of germ cells based on nuclear and whole-cell nascent unspliced mRNA. (A) UMAP showing distribution of germ cells based entirely on unspliced mRNA (n = 8 mice and 13 rats). Cell type designations are shown (top) assigned based only on nascent mRNA and (bottom) the same as Figure 1, based on whole cell/nuclear mRNA. (B) Comparison of cell designations based off whole cell/nuclear mRNA and based upon unspliced mRNA only. Shading of elongating spermatids in nuclear assay indicates few cells captured.

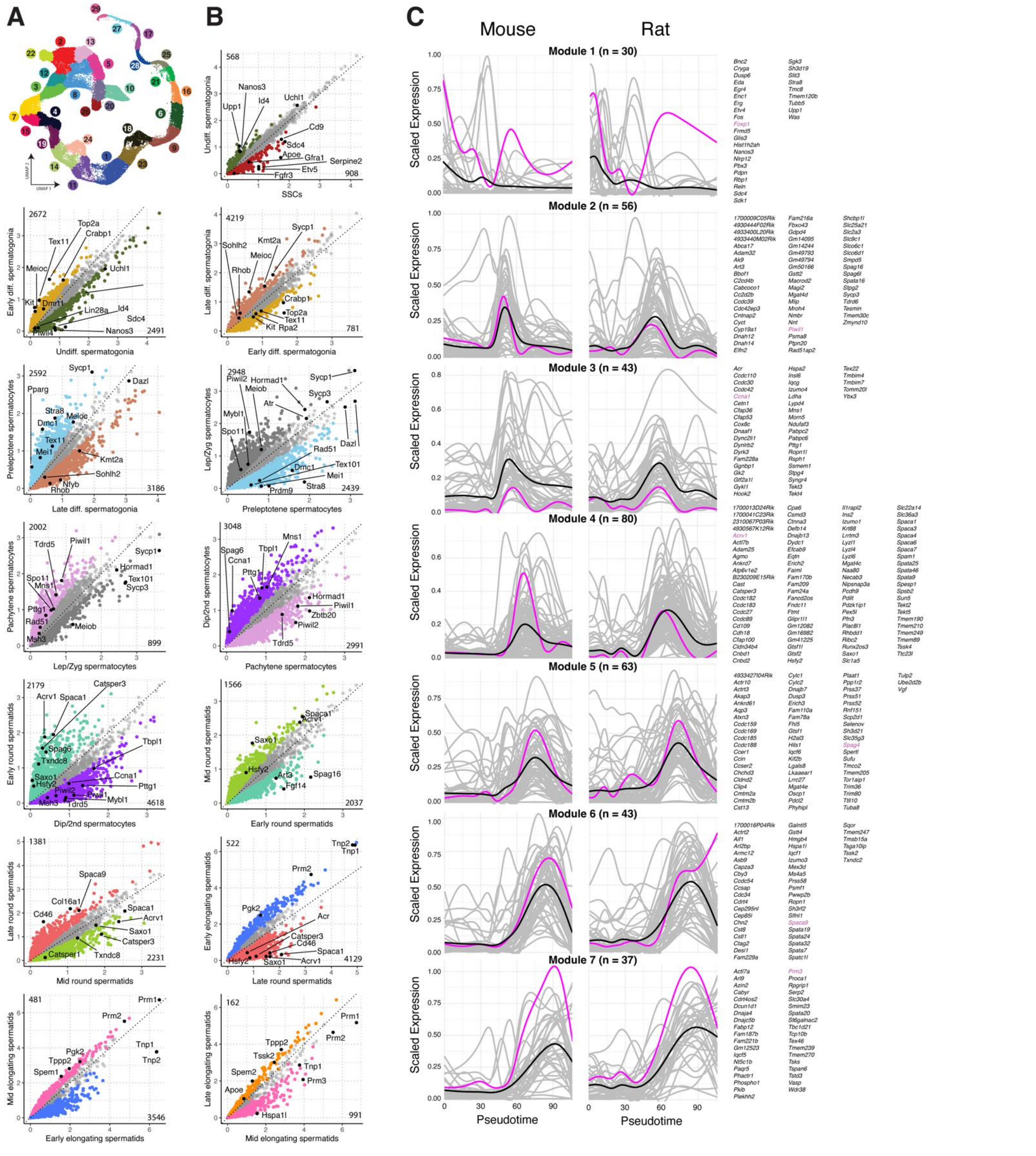
A
B
C
Mouse
Rat
UMAP 1
UMAP 2
Undiff. spermatogonia
SSCs
Early diff. spermatogonia
Late diff. spermatogonia
Preleptotene spermatocytes
Lep/Zyg spermatocytes
Pachytene spermatocytes
Dip/2nd spermatocytes
Early round spermatids
Mid round spermatids
Late round spermatids
Early elongating spermatids
Mid elongating spermatids
Late elongating spermatids
Module 1 (n = 30)
Module 2 (n = 56)
Module 3 (n = 43)
Module 4 (n = 80)
Module 5 (n = 63)
Module 6 (n = 43)
Module 7 (n = 37)
Scaled Expression
Pseudotime

Figure S4. Differential gene expression between cell types and modules of correlated gene expression between species. (A) Unbiased clustering of integrated mouse and rat germ cells. (B) Normalized gene expression shown for each pairwise comparison of sequential cell types. Significant (adjusted p value < 0.05) differentially expressed genes displaying >1.5-fold difference are colored by cell type. (C) Genes that correlate in expression between mouse and rat ($r > 0.9$, Pearson's correlation) were selected and modules of genes that behave similarly across pseudotime were calculated. For each module, expression (scaled to a percentage of maximum ex-pression) of all genes in the module by species is shown and one representative gene has been highlighted in magenta. All data in this figure were generated from sn/scRNA-seq (n = 8 mice and 13 rats).

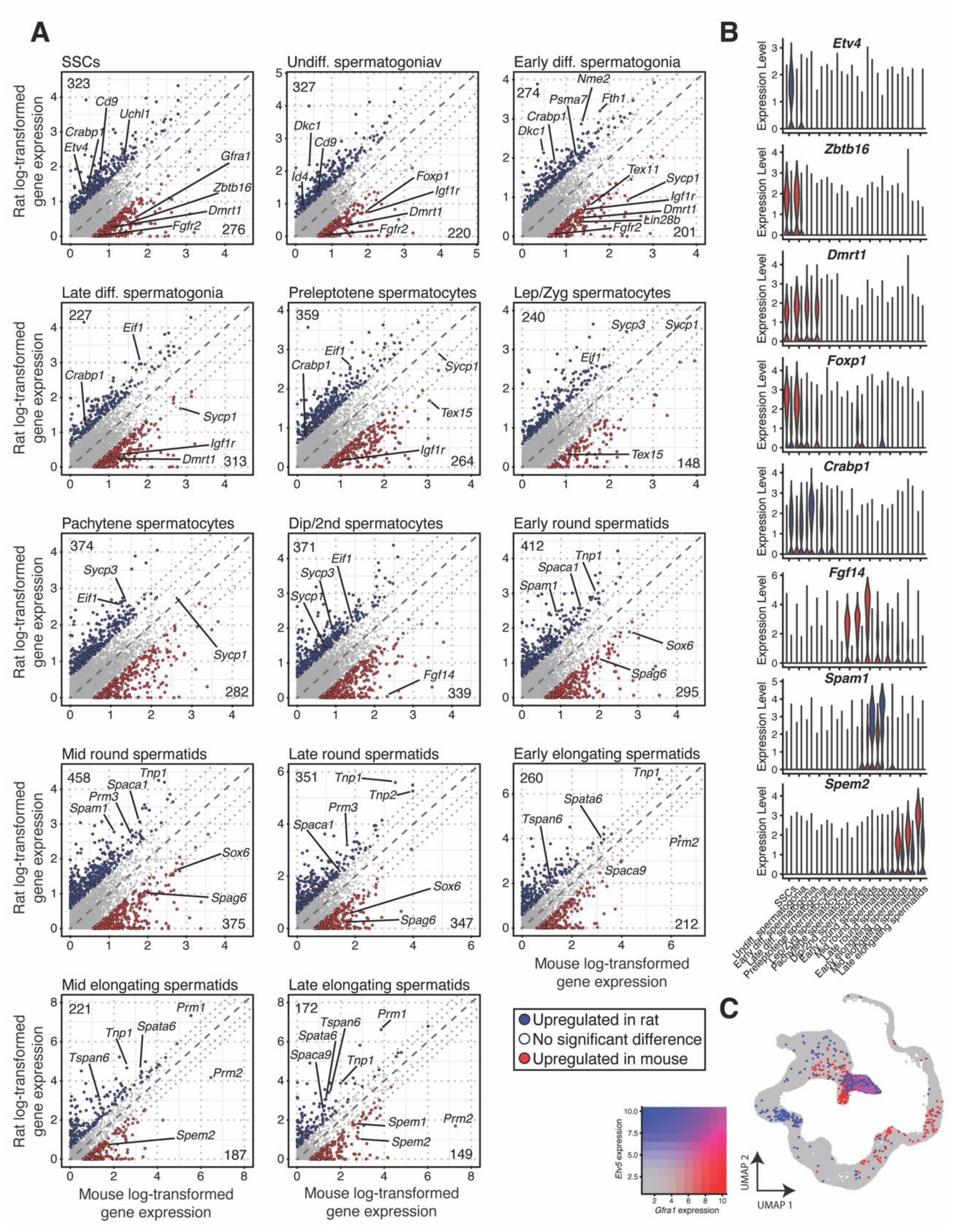

Figure S5. Differential gene expression between species. (A) Normalized gene expression shown for each cell type showing the pairwise comparison between mice and rats. Significant (adjusted *p* value < 0.05) differentially expressed genes showing a minimum of 10% difference in

proportion of cells expressing the gene as well as displaying >1.5-fold difference are colored. (B) Violin plots showing normalized gene expression of selected genes significantly different between species in at least one cell type. (C) Co-localization of *Etv5* and *Gfra1* in spermatogonia, colored by normalized gene expression. All data in this figure were generated from sn/scRNA-seq (n = 8 mice and 13 rats).

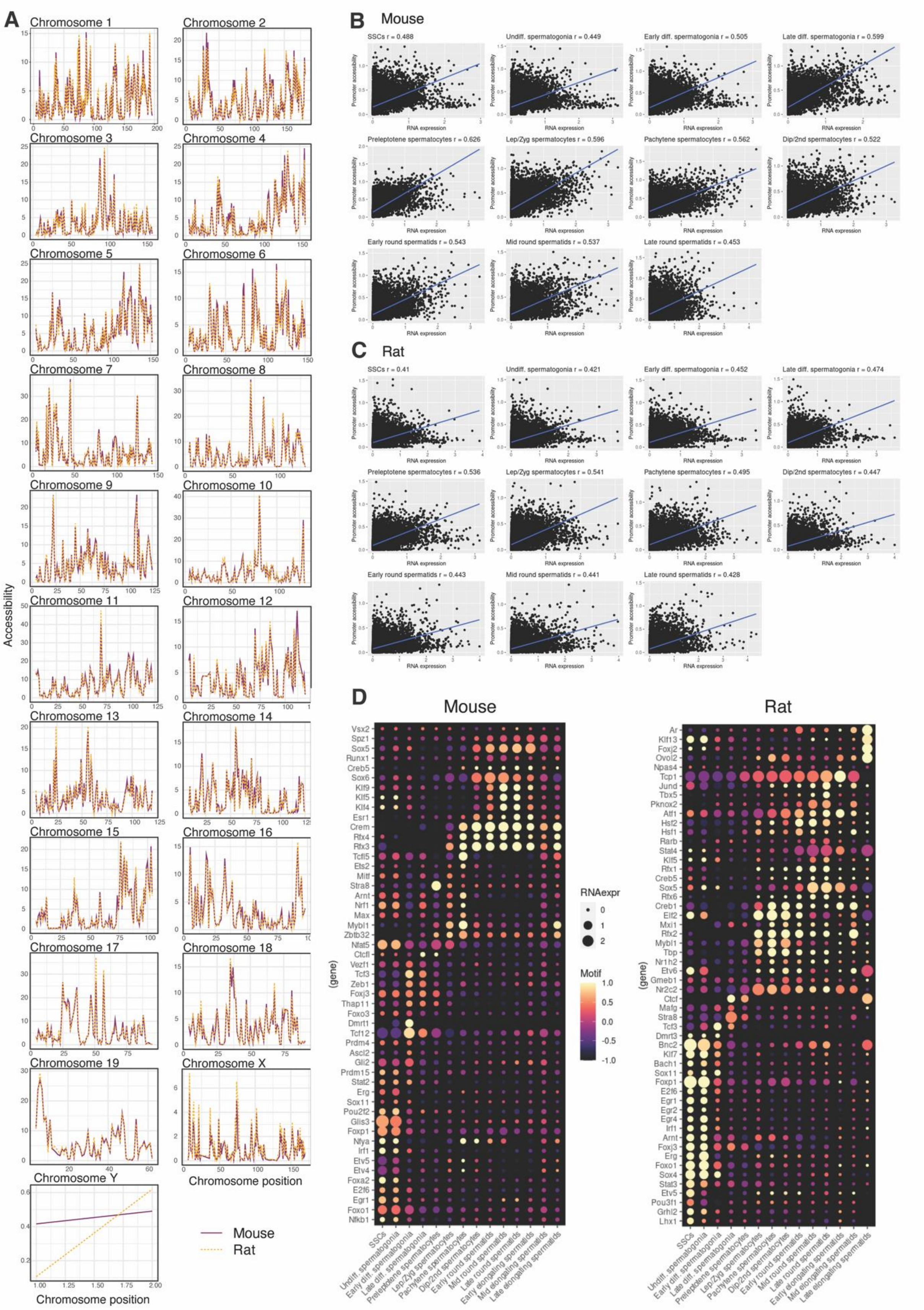
A
Chromosome 1
Chromosome 2
Chromosome 3
Chromosome 4
Chromosome 5
Chromosome 6
Chromosome 7
Chromosome 8
Chromosome 9
Chromosome 10
Chromosome 11
Chromosome 12
Chromosome 13
Chromosome 14
Chromosome 15
Chromosome 16
Chromosome 17
Chromosome 18
Chromosome 19
Chromosome X
Chromosome Y
Accessibility
Chromosome position
Mouse
Rat
B Mouse
SSCs r = 0.488
Undiff. spermatogonia r = 0.449
Early diff. spermatogonia r = 0.505
Late diff. spermatogonia r = 0.599
Preleptotene spermatocytes r = 0.626
Lep/Zyg spermatocytes r = 0.596
Pachytene spermatocytes r = 0.562
Dip/2nd spermatocytes r = 0.522
Early round spermatids r = 0.543
Mid round spermatids r = 0.537
Late round spermatids r = 0.453
Promoter accessibility
RNA expression
C Rat
SSCs r = 0.41
Undiff. spermatogonia r = 0.421
Early diff. spermatogonia r = 0.452
Late diff. spermatogonia r = 0.474
Preleptotene spermatocytes r = 0.536
Lep/Zyg spermatocytes r = 0.541
Pachytene spermatocytes r = 0.495
Dip/2nd spermatocytes r = 0.447
Early round spermatids r = 0.443
Mid round spermatids r = 0.441
Late round spermatids r = 0.428
D
Mouse
Rat
RNAexpr
Motif
(gene)
SSCs
Undiff. spermatogonia
Early diff. spermatogonia
Late diff. spermatogonia
Preleptotene spermatocytes
Lep/Zyg spermatocytes
Pachytene spermatocytes
Dip/2nd spermatocytes
Early round spermatids
Mid round spermatids
Late round spermatids
Early elongating spermatids
Mid elongating spermatids
Late elongating spermatids

Figure S6. Chromatin accessibility and gene expression. (A) Chromatin accessibility across all chromosomes. Each chromosome was divided into 10^6 bp bins and normalized peak counts were sampled and summed to the species minimum number of cells observed. (B) Correlation of promoter accessibility with mRNA expression in mouse and rat. Correlation coefficient (r) shown for each cell type, and blue line denotes linear model. (C) Transcription factor and motif accessibility correlation. Normalized expression of transcription factors selected with high motif correlation coeffects scaled across rows. Expression is denoted by size of dot and motif represented by chromVAR motif deviation score shown by color. All data in this figure were generated from snRNA/ATAC-seq multiomic profiling (n = 6 mice and 4 rats).

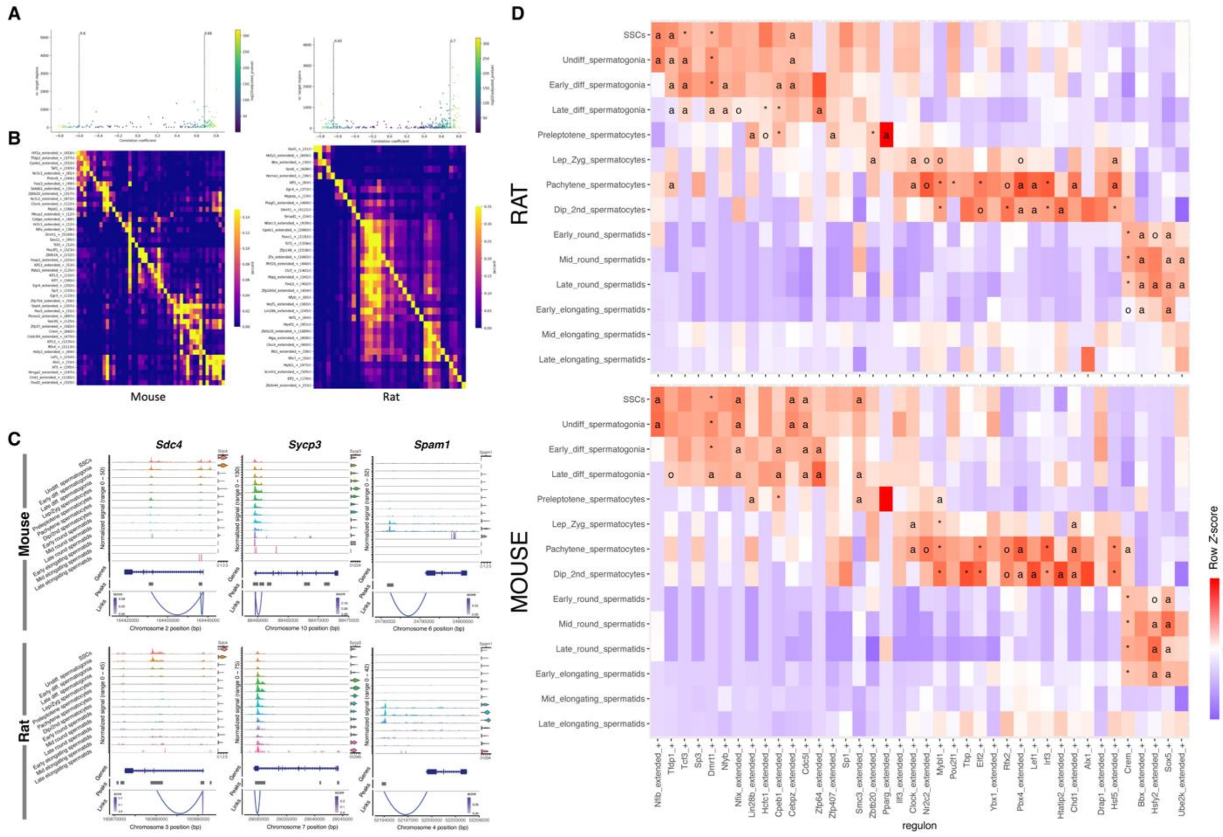

Figure S7. Cross-species conservation of gene regulatory networks in spermatogenesis. (A) Thresholds for GRN analysis. Scatter plot showing number of target regions versus TF expression-to-region AUC Pearson correlation coefficients for each regulon inferred for each species are shown. Regulons were filtered based on a threshold on the correlation coefficient, indicated by the dotted line as part of SCENIC+ pipeline. (B) Overlap of target regions of positive-regulated regulons by species. The overlap is divided by the number of target regions of the regulon in each row, yielding Jaccard similarity index. The color of the upper limit is set manually for visualization purposes. (C) Conserved putative regulatory regions in and around important spermatogenic genes. Regions matched by liftover of the rat genome onto mouse. Significant peak linkage shown. (D) Classification of chromatin effects. Heatmap of TF gene expression, overlaid with assessment of

TF activity, where “open” is a significant enrichment of TF cistromes in DARs and “active” indicates the mean AUC exceeds the regulon threshold. a = expression + active, o = expression + open, * = expression + active + open. All data in this figure were generated from snRNA/ATAC-seq multiomic profiling (n = 6 mice and 4 rats).

Target	Clone	Gene	Catalog number	Manufacturer	Type	Appl	ADT sequence
F4/80	BM8	*Adgre1*	TotalSeq™-B0114	Biolegend	Primary antibody	ADT	TTAACTTCAGCCCGT
IBA1	Polyclonal	*Aif1*	019-19741	Fujifilm Wako Chemicals	Primary antibody	ADT	-
Rat *Aif1*	-	*Aif1*	427068	ACD	RNA Probe	ISH	-
CD13	WM15	*Anpep*	TotalSeq™-B0364	Biolegend	Primary antibody	ADT	TTTCAACGCCCTTTC
CD317	927	*Bst2*	TotalSeq™-B0811	Biolegend	Primary antibody	ADT	TGTGGTAGCCCTTGT
CD274	MIH6	*Cd274*	TotalSeq™-B0190	Biolegend	Primary antibody	ADT	TCGATTCCACCAACT
CD52	HI186	*Cd52*	TotalSeq™-B0033	Biolegend	Primary antibody	ADT	CTTTGTACGAGCAAA
CD55	RIKO-3	*Cd55*	TotalSeq™-B0558	Biolegend	Primary antibody	ADT	ATTGTTGTCAGACCA
CD59	p282 (H19)	*Cd59*	TotalSeq™-B0361	Biolegend	Primary antibody	ADT	AATTAGCCGTCGAGA
CD9	MZ3	*Cd9*	TotalSeq™-B0813	Biolegend	Primary antibody	ADT	TAGCAGTCACTCCTA
CD9	2A1/CD9	*Cd9*	206506	Biolegend	Primary antibody	ADT/ FACS	-
Rat *Etv5*	-	*Etv5*	427068	ACD	RNA Probe	ISH	-
CD142	NY2	*F3*	TotalSeq™-B0822	Biolegend	Primary antibody	ADT	CACTGCCGTCGATTA
CD32	FUN-2	*Fcgr2a*	TotalSeq™-B0142	Biolegend	Primary antibody	ADT	GCTTCCGAATTACCG
Rat *Gfra1*	-	*Gfra1*	463031-C2	ACD	RNA Probe	ISH	-
GFRA1/CD326 (rat)	Polyclonal	*Gfra1*	BAF560	R&D Systems	Primary antibody	ADT/IHC/ MACS	-
GFRA1/CD326 (mouse)	98.8	*Gfra1*	118203	Biolegend	Primary antibody	ADT/ MACS	-
CD278	C398.4A	*Icos*	TotalSeq™-B0171	Biolegend	Primary antibody	ADT	CGCGCACCCATTAAA
Rat *Id4* (C2)	-	*Id4*	873098-C2	ACD	RNA Probe	ISH	-
KIT	2B8	*Kit*	TotalSeq™-B0012	Biolegend	Primary antibody	ADT	TGCATGTCATCGGTG
KIT	Polyclonal	*Kit*	ITA1763	G Biosciences	Primary antibody	ADT	-
CD205	HD30	*Ly75*	TotalSeq™-B0814	Biolegend	Primary antibody	ADT	CTATCGTTTGATGCA
CD56	5.1H11	*Ncam1*	TotalSeq™-B0047	Biolegend	Primary antibody	ADT	TCCTTTCCTGATAGG
CD273	24F.10C12	*Pdcd1lg2*	TotalSeq™-B0008	Biolegend	Primary antibody	ADT	TCAACGCTTGGCTAG
SPAM1	Polyclonal	*Spam1*	A2120	ABClonal Science Inc	Primary antibody	ADT	-
CD302	Polyclonal		LS-C119435	Lifespan Biosciences	Primary antibody	ADT	-
Biotin	-	-	TotalSeq™-B0952	Biolegend	Secondary antibody	ADT	CAGGTTGTTGTCATT
Biotin	-	-	TotalSeq™-B0953	Biolegend	Secondary antibody	ADT	GTCCGACTAATAGCT
Anti-APC	APC003	-	TotalSeq™-B0987	Biolegend	Secondary antibody	ADT	TTAACCGTCTCCCTT
Anti-PE	PE001	-	TotalSeq™-B0911	Biolegend	Secondary antibody	ADT	TGACCAGTTCCGCAT
Anti-FITC	FIT-22	-	TotalSeq™-B0988	Biolegend	Secondary antibody	ADT	-

Table S5. Antibodies and probes used in this study. "Target" denotes the reactive protein or mRNA. Appl = Application. ADT = antibody derived tags used for feature barcoding. IHC = immunohistochemistry. ISH = in situ hybridization. MACS = magnetically activated cell sorting. FACS = fluorescently activated cell sorting.

SUPPLEMENTARY METHODS

Selection of cell fractions

For rat samples selected for EpCAM, cells were incubated with mouse anti-rat EpCAM antibody (clone GZ1 produced by Dr Gottfried Dohr in the Medical University of Graz, Austria[1]) for 20 minutes at 4°C. Mouse samples instead were selected with anti-mouse EpCAM clone 98.8 (see Table S5). Samples were washed twice in PBS-S, resuspended and incubated with anti-mouse secondary antibody conjugated to magnetic microbeads (Miltenyi) for 20 minutes at 4°C. Cells were washed twice and selected via MACS MS columns (Miltenyi). Sample viability was typically 90-95% and no samples had viability below 80%. Flow cytometry was performed on a FACSCanto II Flow Cytometry System (BD Bioscience) and sorting on FACSAria II Cell Sorter (BD Bioscience) using the same antibodies as for ADT conjugated with APC/PE/FITC as appropriate (see Table S5) and for CD9 sorting the gating system is shown in **Figure 6M**.

Antibody labeling.

Cells were incubated with primary and secondary antibodies as described in Table A. 1-2 million cells were suspended per sample in 50 µl Cell Staining Buffer (Biolegend) with 0.5ul of TruStain FcX PLUS (anti-mouse CD16/32) Blocking Reagent. After 10 minutes of incubation at 4°C, an antibody cocktail containing all of the TotalSeq antibodies for that experiment. In the cases where an ADT secondary was used, the antibodies were stained in series with three washes in between to limit cross-reactivity. For each incubation, cells were incubated for 30 minutes at 4°C and then spun down at 400 × *rcf* followed by three washes. Washes were performed by adding 1ml for each wash and spinning down at 400 × *rcf.*

Nuclei preparation

Nuclei were prepared according to 10X's protocol CG000366 Rev B with changes as noted. Briefly, 10^5 cells were centrifuged at 500 × *rcf* for 5 minutes at 4C (all subsequent spins used the same conditions). 100ul of chilled 0.1X Lysis buffer was added, consisting of nuclease-free water with final concentrations after dilution: 10mM Tris-HCL (pH7.4, Sigma-Aldrich), 10mM NaCl (Sigma-Aldrich), 3mM $MgCl_2$ (Sigma-Aldrich), 0.01% Tween-20 (Thermo Fisher Scientific), 0.01% non-idet P40 substitute (Sigma-Aldrich), 0.001% digitonin (Thermo Fisher Scientific), 1% BSA (Miltenyi Biotech), 1mM DL-Dithiothreitol (DTT, Sigma-Aldrich), 1 U/µl sigma protector RNase inhibitor (Sigma-Aldrich). Cells were digested for 2 minutes on ice before resulting nuclei were pelleted. Nuclei were washed three times in a wash buffer identical to the lysis buffer lacking the P40 substitute and digitonin. Finally, nuclei were suspended in nuclei buffer (10X) with 1mM DTT and 1 U/ µl RNAse inhibitor.

Differential accessibility and pathway analysis

Differentially-accessible peaks were identified via Seurat's FindMarkers function with default options (i.e., the Wilcoxon rank-sum test). *TxDb.Mmusculus.UCSC.mm10.knownGene* and *TxDb.Rnorvegicus.UCSC.rn6.refGene* were used with ChIPseeker [2] to annotate peaks by relative locations to genes (promoter, intergenic, etc). All peak visualization was performed using Signac's CoveragePlot. Motif accessibility was calculated via ChromVAR [3] using the JASPAR2020 [4] set using Core collection and tax_group = vertebrates, and then using Signac's AddMotifs, FindMotifs and RunChromVAR with the appropriate genomes. Promoter activity was calculated with Signac's GeneActivity function using 2000bp upstream of the TSS and 0bp downstream and Pearson's correlations were performed against gene expression on a per-cell basis within each cell type.

Ingenuity Pathway Analysis (QIAGEN Inc, https://www.qiagenbioinformatics.com/products/ingenuity-pathway-analysis) was used for all pathway analyses. In all cases gene lists were used with a minimum fold change cutoff of ± 1.5 and *p*-adjusted value of ≤ 0.05.

Rat genome annotations

Rat genome annotations and peak calls were lifted to mouse coordinates using UCSC's LiftOver tool[5]. For both mouse and rat, many of the lncRNA names were arbitrary identifiers assigned after genome assembly. To coordinate orthologous lncRNAs, mouse lncRNA positions were queried against the lifted rat lncRNA coordinates with PyRanges[6]. If multiple mouse lncRNAs overlapped a single, lifted rat lncRNA, then the gene with largest %overlap was selected as the orthologous annotation. To assess chromosome-scale accessibility, the positions of lifted rat peaks and overlapping mouse peaks were organized into 10^6 bp bins. For each cell type, normalized peak counts were sampled to the specie minimum number of cells observed and summed. To analyze expression of individual *Gfra1* exons, a second rat reference was created where each exon of *Gfra1* was annotated in the GTF as a separate gene and counts calculated for all cells and processed in the same manner as the main analysis, but only used for the purpose of *Gfra1* visualization.

Transcriptional regulatory analysis with SCENIC+

The TRN analysis was conducted using SCENIC+ v1.01.dev3+g3741a4b[7]. First, custom cisTarget databases were created by extracting the genomic sequences for each peak feature in the snATAC-seq component of the multiome data, using the mm10 or rn6 genomes for mouse and rat

respectively, and then compiling the databases using the create_cistarget_motif_databases.py tool (https://github.com/aertslab/create_cisTarget_databases). The SCENIC+ pipeline was run largely in accordance with the suggested protocol for 10x multiome data. Briefly, 20 topics were selected for the snATAC-seq data following topic modeling for both mouse and rat and were binarized with both the otsu method and with ntop=3000. The motifs-v10-nr.mgi-m0.00001-o0.0.tbl database was used for the mouse motif annotation. The rat motif annotation was constructed by converting each gene to the closest matching rat ortholog. The run_pycistarget function was implemented with run_without_promoters=True, followed by the run_scenicplus function with upstream=downstream=[1000,150000]. The rat transcription factor collection was obtained through converting mouse transcription factors to the closest matching ortholog. Regulons were filtered qualitatively to retain approximately 80 eRegulons for mouse and rat each. Pseudotime plots were constructed using the plot_potential function with the joint Monocle pseudotime values.

Transcription factor (TF) activity was calculated using methods described by Garcia-Alonso *et al.* [8] adapted for SCENIC+ methods. The criterion for TF expression was as described by Garcia-Alonso *et al.* [8] "Open" TF is defined by significant enrichment of TF cistromes in cell type-specific differentially accessible regions (DARs) as scored by pycistarget[7]. For RNA-based activity, we consider the empirically determined gene targets of each TF regulon. If the mean AUC score for a given cell type is greater than the regulon threshold, then the TF is "active."

Transplantation

Rat samples were enriched for CD9+ cells as described above. Transplants were performed at 3×10^6 cells per ml into NU/J nude mice (Jackson Laboratories 002019). Transplantation procedure was performed as described previously[9]. Host animals were sacrificed after 4 months

according to institutional guidelines. Testes were extracted, weighed and the tunica removed. For encapsulation, cells were prepared as described above. For colony counting, transplanted testes were stained with X-gal as described previously[10].

Immunohistochemistry and *in situ* RNA hybridization

One 4-month-old male HomoMTLacZ rat was sacrificed according to university animal use protocols. Both right and left testes were removed and placed individually into 2 mL of Hank's balanced salt solution (Gibco #14175079) in well of a 6 well culture plate. The tunica albuginea was removed, and the tissue was transferred into a 50 mL tube filled to 50 mL with 10% formalin (Fisher #23245684), then shaken slowly for 24 hours at room temperature. Tissue was dehydrated by a standard ethanol series followed by xylene, then finally embedded in paraffin using standard procedures. Paraffin blocks were sectioned at 5 um using a microtome. Staging of the seminiferous tubules was performed according to Russel *et al.* 1993[11] using FFPE sections stained with hematoxylin-eosin (HE) and Periodic acid-Schiff (PAS). HE and PAS histochemistry was performed following the protocols of the Armed Forces Institute of Pathology[12].

For multiplex *in situ* hybridization (ISH), the RNAscope® technology was employed. RNAscope® (ISH) was carried out on a Leica BOND RXm platform according to manufacturer's protocols. Probes used are detailed in Table S5. Multiplex RNAscope® probes and antibody codetection was also carried out on a Leica BOND RXm platform according to manufacturer's protocols. Immunohistochemistry/immunofluorescence was performed as described elsewhere[13] using the primary antibodies listed in Table S5.

1. Schiechl, H. & Dohr, G. Immunohistochemical studies of the distribution of a basolateral-membrane protein in intestinal epithelial cells (GZ1-Ag) in rats using monoclonal antibodies. *Histochemistry* **87**, 491-498 (1987).
2. Wang, Q. *et al.* Exploring Epigenomic Datasets by ChIPseeker. *Curr Protoc* **2**, e585 (2022).
3. Schep, A.N., Wu, B., Buenrostro, J.D. & Greenleaf, W.J. chromVAR: inferring transcription-factor-associated accessibility from single-cell epigenomic data. *Nature methods* **14**, 975-978 (2017).
4. Rauluseviciute, I. *et al.* JASPAR 2024: 20th anniversary of the open-access database of transcription factor binding profiles. *Nucleic Acids Res* **52**, D174-D182 (2024).
5. Perez, G. *et al.* The UCSC Genome Browser database: 2025 update. *Nucleic Acids Res* (2024).
6. Stovner, E.B. & Saetrom, P. PyRanges: efficient comparison of genomic intervals in Python. *Bioinformatics* **36**, 918-919 (2020).
7. Bravo Gonzalez-Blas, C. *et al.* SCENIC+: single-cell multiomic inference of enhancers and gene regulatory networks. *Nature methods* **20**, 1355-1367 (2023).
8. Garcia-Alonso, L. *et al.* Single-cell roadmap of human gonadal development. *Nature* **607**, 540-547 (2022).
9. Sinha, N., Whelan, E.C. & Brinster, R.L. Isolation, Cryopreservation, and Transplantation of Spermatogonial Stem Cells. *Methods Mol Biol* **2005**, 205-220 (2019).
10. Nagano, M., Avarbock, M.R. & Brinster, R.L. Pattern and kinetics of mouse donor spermatogonial stem cell colonization in recipient testes. *Biology of reproduction* **60**, 1429-1436 (1999).
11. Russell, L.D., Ettlin, R.A., Hikim, A.P.S. & Clegg, E.D. Histological and Histopathological Evaluation of the Testis. *International Journal of Andrology* **16**, 83-83 (1993).
12. LH, P.E.M.B.S. *Laboratory Methods in Histotechnology*. (American Registry of Pathology, Washington DC, USA; 1992).
13. Tarrant, J.C. *et al.* Pathology of macrophage activation syndrome in humanized NSGS mice. *Res Vet Sci* **134**, 137-146 (2021).
