# Supplementary material for: Single-cell multiomic comparison of mouse and rat spermatogenesis reveals gene regulatory networks conserved for over 20 million years
Source: Stem Cell Reports. 2025 Mar 13;20(4):102449. doi: 10.1016/j.stemcr.2025.102449 (PMC12069898; doi:10.1016/j.stemcr.2025.102449)
Supplement: Document S2. Article plus supplemental information [file mmc6.pdf]

Stem Cell Reports
Resource
OPEN ACCESS

Single-cell multiomic comparison of mouse and rat spermatogenesis reveals gene regulatory networks conserved for over 20 million years

Eoin C. Whelan,[1,10,*] John J. Swain,[1,2,6,8] Jonathan H. Sussman,[3,4,5,8] David Smith,[3] Fan Yang,[1,7] Antonia Rotolo,[2] Mary R. Avarbock,[1] Clara Malekshahi,[2] Enrico Radaelli,[2] Daniel P. Beiting,[2] and Ralph L. Brinster[1,9,*]

[1]Department of Biomedical Sciences, University of Pennsylvania, School of Veterinary Medicine, Philadelphia, PA, USA
[2]Department of Pathobiology, University of Pennsylvania, School of Veterinary Medicine, Philadelphia, PA, USA
[3]Children's Hospital of Philadelphia, Philadelphia, PA, USA
[4]Medical Scientist Training Program, Perelman School of Medicine, University of Pennsylvania, Philadelphia, PA, USA
[5]Graduate Group in Genomics and Computational Biology, Perelman School of Medicine, University of Pennsylvania, Philadelphia, PA, USA
[6]Present address: Department of Pathobiology, University of Pennsylvania, School of Veterinary Medicine; Philadelphia, Pennsylvania, USA
[7]Present address: Yangzhou University Medical College; Yangzhou, China
[8]These authors contributed equally
[9]Senior author
[10]Lead contact
*Correspondence: ewhelan@vet.upenn.edu (E.C.W.), brinster@vet.upenn.edu (R.L.B.)
https://doi.org/10.1016/j.stemcr.2025.102449

SUMMARY

Spermatogenesis is driven by dramatic changes in chromatin regulation, gene transcription, and protein expression. To assess the mechanistic bases for these developmental changes, we utilized multiomic single-cell/nucleus RNA sequencing (sc/snRNA-seq) and single-nucleus assay for transposase-accessible chromatin with sequencing (snATAC-seq) to identify chromatin changes associated with transcription in adult mouse and rat testes. We characterized the relationships between the transcriptomes and chromatin of both species, including the divergent expression of *Id4* in spermatogonial stem cells between species. Promoter accessibility and gene expression showed the greatest association during meiosis in both species. We mapped the cross-species conservation of putative regulatory regions for key spermatogenic genes, including *Cd9* and *Spam1*, and investigated correlations and disconnects in chromatin accessibility, gene expression, and protein expression via antibody-derived tags. Using a gene regulatory network (GRN) model, we identified 40 core regulons conserved between mouse and rat germ cells, highlighting the relevance of chromatin-related factors in regulating the transcription of canonical genes across spermatogenesis.

INTRODUCTION

During spermatogenesis, undifferentiated spermatogonial stem cells (SSCs) differentiate to produce all male germ cells in the adult testis. Proliferating mitotic spermatogonia differentiate into spermatocytes that undergo meiosis to become spermatids that mature into spermatozoa (Russell et al., 1993). The complex cellular changes accompanying spermatogenesis are driven by complex changes in gene expression, as evidenced by single-cell mRNA sequencing (scRNA-seq) throughout spermatogenesis in mouse (Green et al., 2018; Hermann et al., 2018; La et al., 2018; Lukassen et al., 2018; Ernst et al., 2019; Grive et al., 2019; Jung et al., 2019), human (Guo et al., 2018; Hermann et al., 2018; Wang et al., 2018; Sohni et al., 2019; Salehi and Totonchi 2023; Li et al., 2024; Wu et al., 2024), and other mammalian species including macaques (Lau et al., 2020; Shami et al., 2020), sheep (Yang et al., 2021; Wu et al., 2022a), pigs (Zhang et al., 2021, 2022a), dairy goats (Yu et al., 2021), buffalo (Huang et al., 2023), yak (Mipam et al., 2023; Wang et al., 2023), and giant pandas (Zheng et al., 2022). However, despite its ability to resolve differentiation trajectories with high resolution, scRNA-seq is unable to identify gene regulatory mechanisms or post-transcriptional impacts on protein abundance that could influence spermatogenesis. Single-cell epigenetic and proteomic studies of mammalian spermatogenesis (Wu et al., 2022b; Zhang et al., 2022b) have shed light on the regulatory and post-transcriptional mechanisms of spermatogenesis, but, to date, an integrated and comprehensive cross-species analysis of changes in chromatin structure, transcription, and protein expression has not yet been undertaken. Both mouse and rat are long-standing models of mammalian spermatogenesis, and the developmental process from stem cells to sperm has been firmly established (Russell et al., 1993), so these are vital species to dissect the molecular events driving differentiation.

In this work, we have established a multiomic analysis of mouse and rat spermatogenesis, comparing and contrasting multi-modal fingerprints of cell states between the two species. Specifically, we used single-cell/nucleus RNA sequencing (sc/snRNA-seq) and single-nucleus assay for transposase-accessible chromatin with sequencing (snATAC-seq) jointly recorded in the same cells, which provided an unprecedented comparison of the gene regulatory mechanisms driving transcriptomic shifts during

© 2025 The Authors. Published by Elsevier Inc. on behalf of International Society for Stem Cell Research. This is an open access article under the CC BY license (http://creativecommons.org/licenses/by/4.0/).

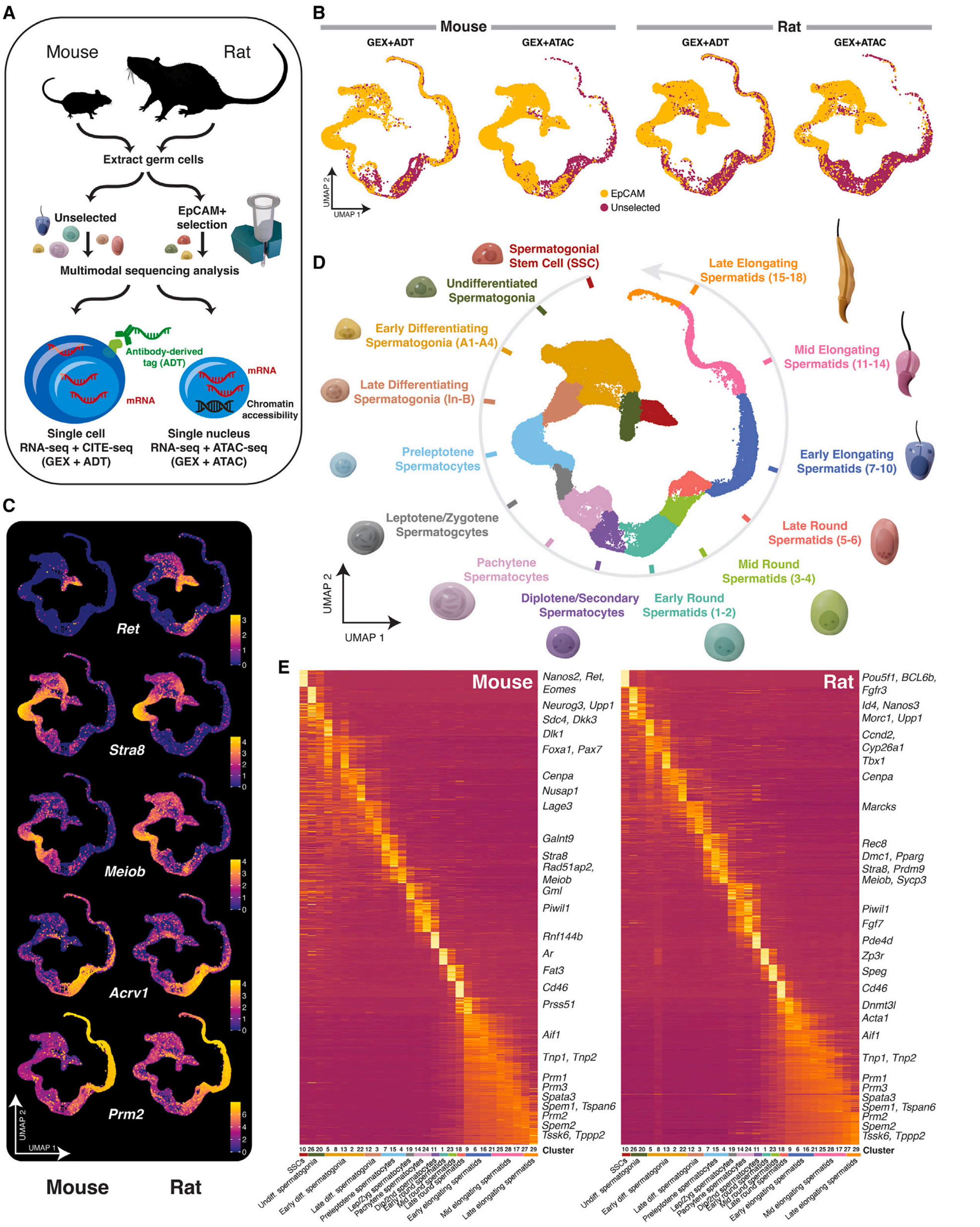

(legend on next page)

spermatogenesis. We found regulatory regions that correlated with gene expression of known germ cell-related genes. In addition, we applied cellular indexing of transcriptomes and epitopes by sequencing (CITE-seq) in order to jointly capture global cellular transcription with selected protein expression. These joint profiling techniques are particularly important for assessing the mechanistic basis of spermiogenesis, as a variety of post-transcriptional controls can delay translation of spermiogenic genes, thus de-coupling transcript and protein levels. This study elucidates a core set of conserved regulons that are responsible for regulating multiple steps of the spermatogenic trajectory.

RESULTS

To mechanistically assess the molecular events accompanying spermatogenesis, testis cells from adult *Rattus norvegicus* as well as adult *Mus musculus* were isolated and analyzed along one of two single-cell workflows: (1) as nuclei jointly assayed for gene expression via snRNA+ATAC-seq and (2) as whole cells jointly assayed for gene and protein expression using CITE-seq (Figure 1A). Taken together, these assays enabled us to directly connect epigenetic changes, gene transcription, and selected protein expression within single cells.

Unbiased scRNA-seq clustering recapitulates spermatogenic processes

To determine the populations of germ cells captured by the single-cell multiomic assays in an unbiased fashion, the shared sc/snRNA-seq assay was used to computationally integrate samples, and cells were clustered and manually annotated based on their gene expression patterns. Somatic cells were removed from the analysis (Figure S1). We captured 23,000 and 17,544 cells by snRNA+ATAC-seq and 17,489 and 54,120 cells by CITE-seq for mouse and rat, respectively (Figure 1B). To adequately resolve the molecular profiles of rare pre-meiotic cells, especially SSCs that comprise only 0.03% of mouse testis cells (Tegelenbosch and de Rooij 1993), we applied these workflows to EpCAM-selected cells known to be enriched for germ cell progenitors (Whelan et al., 2022) as well as unselected mouse and rat testis cells in which post-meiotic cells make up the majority of germ states (Figure 1B). Reproducibility between and within individuals was high across assays (Figures S2A–S2K). Integrated datasets were then clustered in an unsupervised manner by species (Figures S2L and S2M). To improve the resolution of each cell differentiation stage and capture the entire process of spermatogenesis with no discontinuations, we integrated our multimodal datasets for both species based on homologous genes and characterized the transcriptome of each differentiation state based on both the nuclear and whole-cell assays. We used an analysis of unspliced RNA as a surrogate for nascent RNA to ensure that the integrated analysis based on nuclear and whole-cell mRNA was faithful to the cell types designated (Figure S3).

Populations displayed known transcribed markers of germ cell differentiation, showing high degree of conservation between mouse and rat in stem cell markers such as *Nanos2* and *Ret*, as well as genes involved in spermatogonial differentiation (*Kit* and *Stra8*), meiosis (*Sycp3* and *Meiob*), and spermiogenesis (*Acrv1*, *Tnp1*, and *Prm2*) (Figure 1C). Certain genes showed repeated stage-specific expression such as *Stra8*, which was detected in both differentiating spermatogonia and preleptotene spermatocytes in mouse and rat. We manually labeled the cell clusters to delineate discrete stages of germ cell maturation based on gene expression, yielding a single unbroken progression along the differentiation pathway of both rat and mouse germ cells (Figures 1D and S4A). All cell types were captured in both assays, with the exception that the terminal stages of germ cell differentiation (e.g., elongating spermatids) were sparse in the single-nucleus data, which we speculate may be due to loss of nuclear integrity of late-stage cells during detergent-based lysis. We observed strong concordance between mouse and rat clusters, indicating that corresponding cell types shared similar gene expression profiles in the two species (Figure 1E).

Transcriptomic similarities between mouse and rat germ cells

While spermatogenesis in the rat is similar enough to mouse that stem cells from the rat can be transplanted into the mouse and produce all differentiating cell types including sperm (Ryu et al., 2005; Whelan et al., 2022), there remain key differences in cell morphology and seminiferous cycling (Russell et al., 1993). All identified germ cell clusters were

Figure 1. Overview of rodent spermatogenesis

(A) Experimental design. Unselected and EpCAM-enriched mouse and rat testicular cells were encapsulated as either nuclei or whole cells for snRNA/ATAC-seq multiomic profiling (n = 6 mice and 4 rats) and CITE-seq (n = 2 mice and 9 rats), respectively.
(B) UMAP projection of unselected and EpCAM+ germ cells from each assay for each species.
(C) Normalized RNA expression of key genes involved in spermatogenesis.
(D) Cell types assigned after unbiased clustering of integrated mouse and rat germ cells.
(E) Heatmap showing gene expression for top 30 genes for each unbiased cluster. Cell type assignments are shown below cluster numbers.

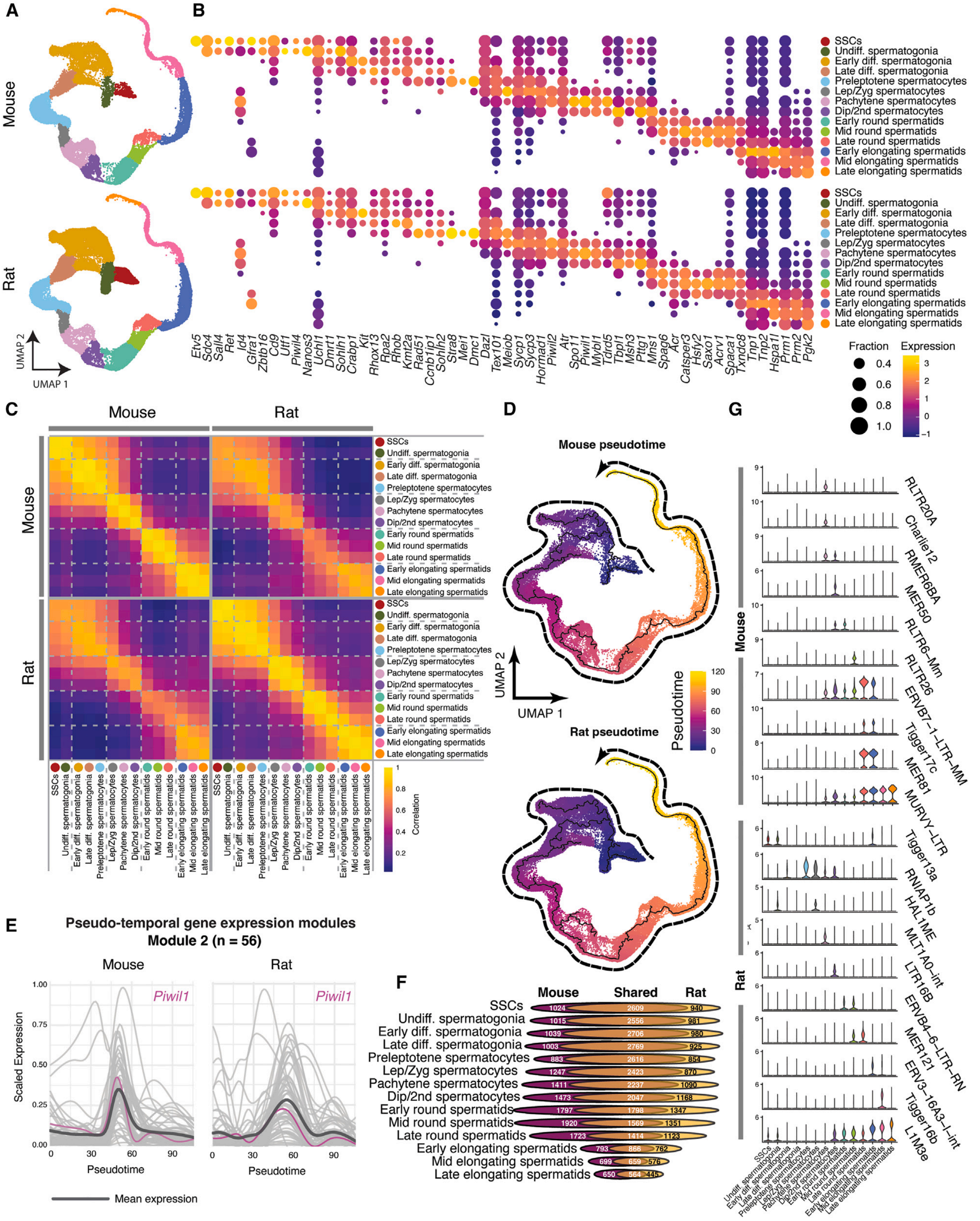

(legend on next page)

present in both mouse and rat (Figure 2A), each displaying a progression of known spermatogenesis markers at appropriate stages, conserved between mice and rats (Figure 2B). Each germ cell type displayed a distinct transcriptional identity as evidenced by differentially expressed genes between stages (Figure S4B). To assess the similarities in transcriptional features during mouse and rat spermatogenesis, we evaluated the correlation of expressed genes between cell types within and between species (Figure 2C). Within species, both mice and rats showed a similar pattern of groupings corresponding to mitotic, meiotic, and spermiogenic phases of spermatogenesis. Within the mitotic phase, a subdivision between undifferentiated and differentiated cells was observed, and spermatids could be divided into round and elongating spermatids, although in both cases these distinctions were more apparent in mouse than rat. Correlations were slightly lower when compared between species, but the overall pattern of cell types was retained. Moreover, despite the differences between mouse and rat spermatogenesis at a morphological level, cell type correlation was highest between any given mouse cell type and its rat counterpart than compared to any different mouse cell type, and likewise for the rat.

Germ cells in both species formed a single progression of cells. This trajectory was assessed by implementing a pseudotime analysis—cells from each species' trajectory were ordered from least differentiated to most differentiated. This allowed for an unbiased ordering of cells to view changes over time in gene expression and chromatin accessibility (Figure 2D). It also allowed for the grouping of genes into modules of genes that are correlated between species and co-regulated across pseudotime (Figures 2E and S4C). Many expressed genes were shared between mouse and rat within cell types, although both the total number of genes expressed and the proportion of shared genes were reduced in later stages of spermatogenesis (Figure 2F; Table S1).

To evaluate the expression of transposable elements (TEs), we employed SoloTE (Rodríguez-Quiroz and Valdebenito-Maturana 2022) to characterize subfamilies of TEs differentially expressed across spermatogenesis (Figure 2G). No subfamilies were specific to spermatogonia in mouse, although a few, such as Tigger13a, were found in rat. However, starting with the onset of meiosis, stage-specific expression of TEs was observed with narrow expression such as Charlie12 and MLT1A0-int in mouse and rat pachytene spermatocytes, respectively, and MER50 and LTR16B in mouse and rat diplotene/secondary spermatocytes. Interestingly, despite the relative transcriptional repression in spermatids, a number of stage-specific TEs can be observed in both species such as Tigger17c in mouse and Tigger16b in rat.

Signaling networks and long non-coding RNA expression are conserved in mouse and rat spermatogenesis

To further understand the changes transpiring across rodent spermatogenesis, we studied the pathways involved at each stage using an Ingenuity Pathway Analysis with stage-specific differentially expressed genes. Stem cells showed an enrichment of pathways associated with stem cell pluripotency, glial-derived neurotropic factor (GDNF) signaling, and mitogen-activated protein kinase (MAPK) signaling, as expected from previous studies examining the proliferation of SSCs in culture (Ishii et al., 2012; Yang et al., 2021) (Figure 3A). Undifferentiated and differentiating spermatogonia showed enrichment for cell cycle regulation and oxidative phosphorylation, consistent with their proliferating nature. Retinoic acid (RA) signaling was apparent in both spermatogonia and preleptotene spermatocytes, corresponding to the stage-dependent and RA-responsive expression of *Stra8* in differentiating spermatogonia (Gewiss et al., 2021; Sinha et al., 2021) and preleptotene spermatocytes (Koubova et al., 2006; Huang et al., 2023) (Figure 1D). Interestingly, genes associated with upregulation of androgen signaling were identified in both mouse and rat premeiotic cells, despite the absence of functional androgen receptors in germ cells (Wang et al., 2009). Such findings suggest an overlap of gene expression between androgen-driven responses in Sertoli cells and germ cell responses to Sertoli cell signaling. Meiotic cells exhibited a dramatic metabolic switch

Figure 2. Differentiation of rodent germ cells

(A) UMAP projection of mouse and rat spermatogenic lineages (all panels in this figure are based on integrated RNA data from 8 mice and 13 rats).

(B) Gene expression of key spermatogenesis genes by cell type. Normalized gene expression is represented by color, whereas the fraction expression of the gene within each cell type is shown (fractions below 0.2 are not shown).

(C) Pearson's correlation of mouse and rat gene expression by cell type between and within species.

(D) Pseudotime for mouse and rat germ cell progression overlaid on UMAP projection.

(E) Representative gene expression module showing genes with similar expression profiles across pseudotime and correlated between species ($r > 0.9$, Pearson's correlation). *Piwil1* expression is highlighted in magenta. Mean expression of all genes is shown by the black line.

(F) Number of expressed genes (normalized expression >0.5) in each species as a scaled Venn diagram per cell type.

(G) Transposable element (TE) expression by cell type for select subfamilies.

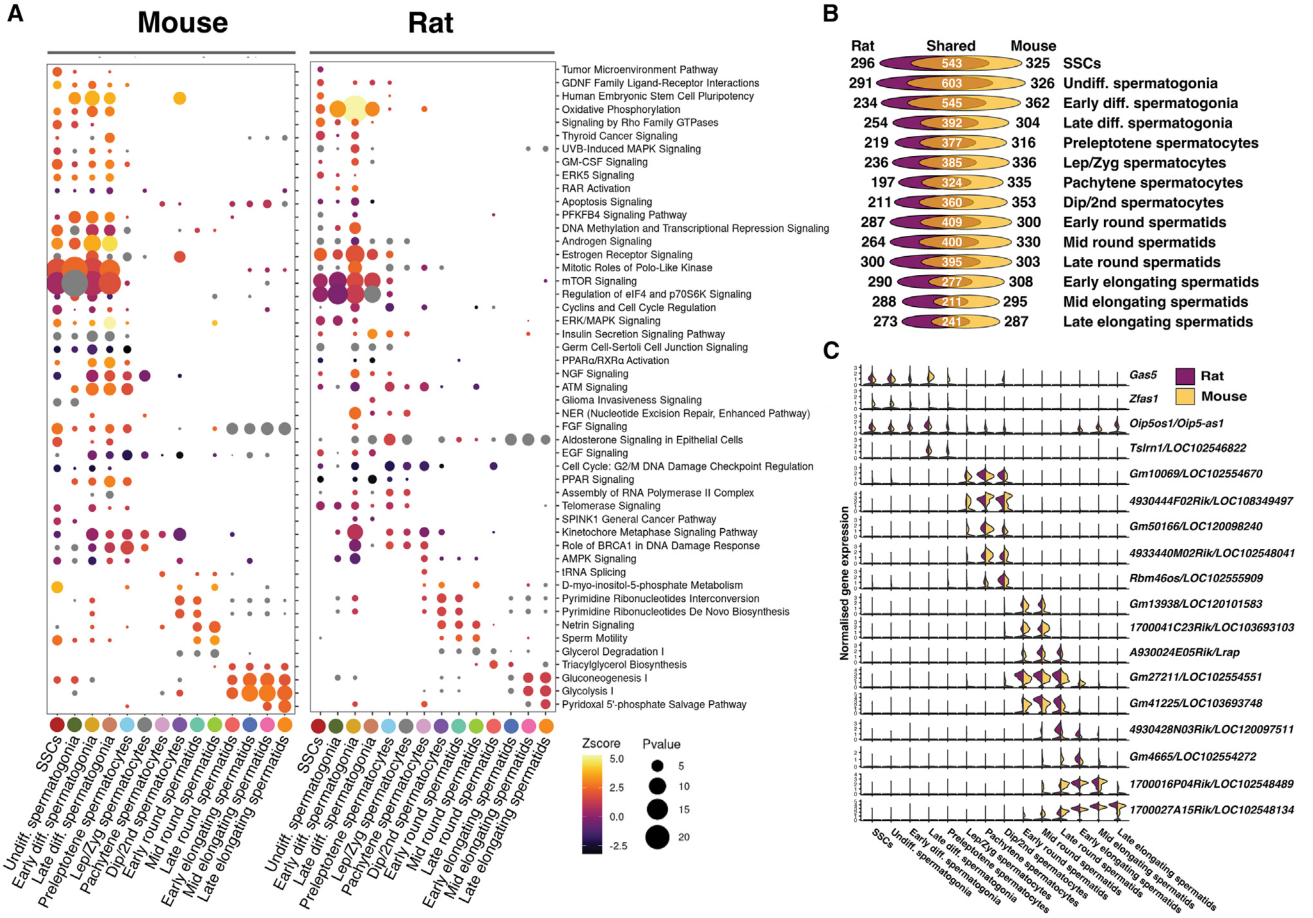

Figure 3. Rodent spermatogenesis involves conserved lncRNA expression and signaling pathways

(A) Pathways enriched at each stage of spermatogenesis for mouse (left) and rat (right) determined using an Ingenuity Pathway Analysis based on differentially expressed genes at each stage. Z score is denoted by color and $-\log_{10} p$ value is shown by dot size.

(B) Number of expressed lncRNAs (normalized expression >0.5) in each species, matched across species using a genomic liftover, as a scaled Venn diagram per cell type.

(C) Normalized expression of select lncRNAs with conserved expression patterns between species.

All data in this figure were generated from integrated sn/scRNA-seq profiling (n = 8 mice and 13 rats).

resulting in upregulation of anaerobic glycolysis and gluconeogenesis in the late stages of spermatogenesis. Numerous long non-coding RNAs (lncRNAs) were expressed in germ cells at various stages. To compare lncRNA expression between species, we took mouse annotations of lncRNAs and inferred orthologous rat loci via a genomic liftover. While the total number of lncRNAs is more consistent between cell types than total mRNA, the proportion of shared lncRNAs does reduce as spermatogenesis proceeds, particularly in elongating spermatids, similar to total mRNA (Figure 3B; Table S2). Of the lncRNAs expressed in both rat and mouse, multiple lncRNAs show exceedingly similar gene expression patterns (Figure 3C). *Gas5* is expressed in undifferentiated spermatogonia of both species, while *Tsirn1* (mouse)/*LOC102546822* (rat) is specifically expressed in late-differentiating spermatogonia. A number of lncRNAs show mid-to-late meiotic expression such as *Gm10069* (mouse)/*LOC102554670* (rat) and *Rbm46os* (mouse)/*LOC102555909* (rat). Still, more show specific expression in round spermatids such as *A930024E05Rik* (mouse)/*Lrap* (rat), and others show high expression in elongating spermatids, for example *1700027A15Rik* (mouse, also known as *Hsf2*)/*LOC102548134* (rat) suggesting an important role in spermiogenesis given the RNA's retention after transcription has ceased (Hong et al., 2021).

Transcriptomic differences between mouse and rat germ cells

To examine differences in spermatogenesis between the species, we identified differentially expressed genes

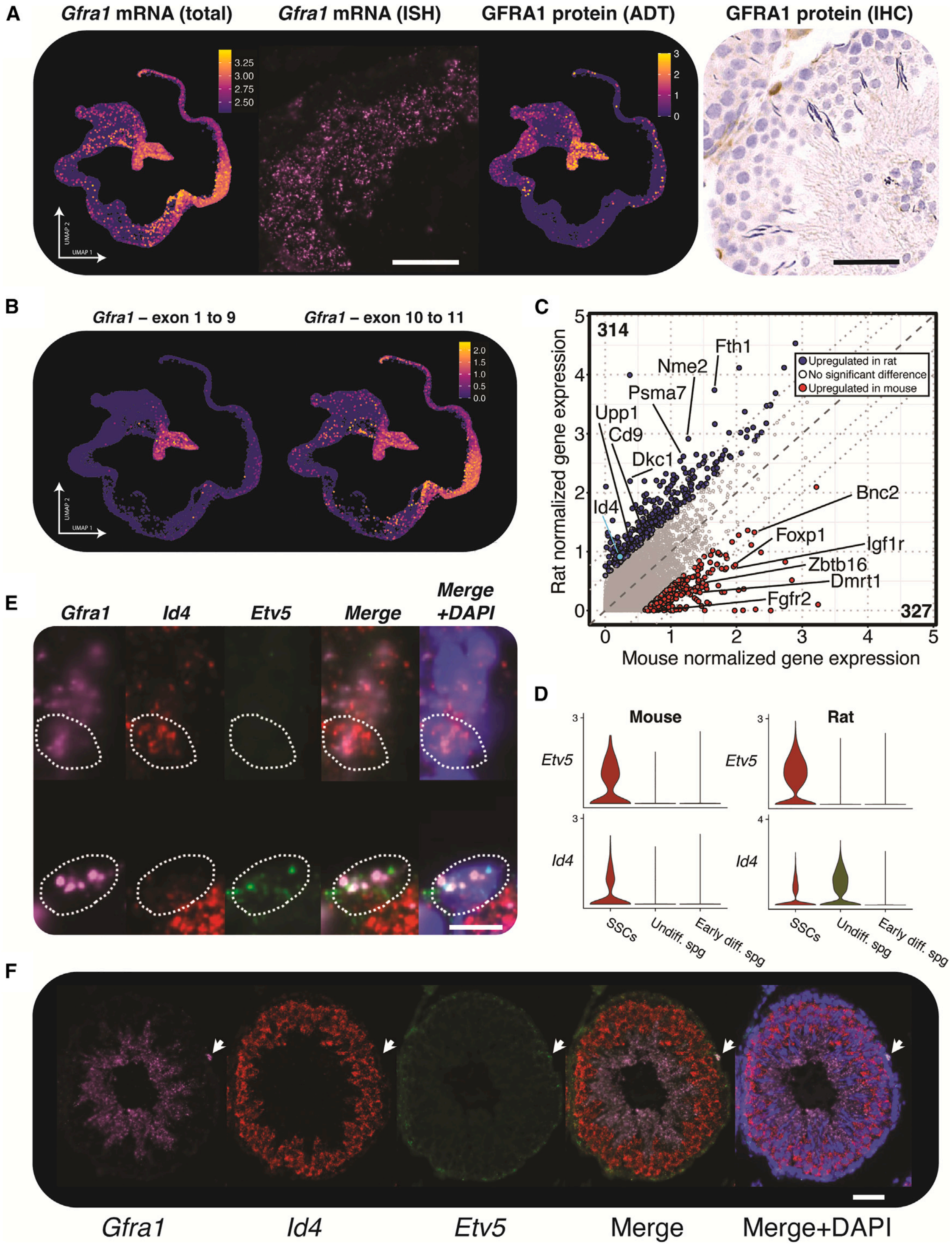

Figure 4. *Id4* and *Etv5* mark distinct populations of spermatogonia in rat

(A) From left to right: *Gfra1* mRNA distribution in rat single-cell germ cell data ($n = 13$), *Gfra1* mRNA localization in rat testis histology via ISH (representative image from 3 replicates; scale bar, 50 μm), GFRA1 protein distribution in rat single-cell data ($n = 5$), GFRA1 protein localization in rat testis histology via IHC (representative image from 3 replicates; scale bar, 50 μm).

(B) *Gfra1* expression counting transcripts that match individual exons ($n = 9$).

(legend continued on next page)

between mice and rat within each cell type separately (Figures S5A and S5B; Table S3). *Etv4*, *Cd9*, and *Crabp1* were comparatively elevated in rat SSCs, whereas *Gfra1*, *Foxp1*, and *Zbtb16* were expressed higher in mouse. *Dmrt1* was more expressed in mouse differentiating spermatogonia, but *Sycp3* was expressed higher in rat spermatocytes. *Fgf14* was expressed higher in mouse spermatocytes, and *Spem2* showed elevated expression in mouse spermatids, whereas *Spam1* and the transition proteins *Tnp1* and *Tnp2* were expressed earlier in rat spermatids than rat. *Prm1* expression in spermatids was higher in the rat than mouse, whereas the inverse is true for *Prm2*, a result that is in accordance with the observation that protamine 2 makes up 67% of mouse protamines but only 2%–5% in rat (Corzett et al., 2002).

To examine transcription factor (TF) expression in SSCs, we first sought to unambiguously identify undifferentiated spermatogonia. *Gfra1* encodes a GDNF receptor critical for retaining stem cell function in SSCs and is a known marker for undifferentiated spermatogonia (Grasso et al., 2012). In the rat, both undifferentiated spermatogonia and early spermatids expressed *Gfra1* mRNA, confirmed in histological sections by *in situ* RNA hybridization, but protein expression was limited to the undifferentiated spermatogonia as measured by CITE-seq and immunohistochemistry (Figure 4A). This discrepancy may be accounted for by the fact that only exons 10 and 11 of *Gfra1* are detected in spermatids (Figure 4B), suggesting that a new *Gfra1* splice variant may account for why some *Gfra1* transcripts are detected in spermiogenesis, and suggests an alternative function of *Gfra1* in late spermatogenesis.

We identified 534 differentially expressed genes between mouse and rat undifferentiated spermatogonia including *Id4*, *Foxp1*, *Igf1r*, *Dmrt1*, and *Upp1* (Figure 4C). As *Id4* is a key marker of SSCs in the mouse (Helsel et al., 2017), it was surprising to see little expression in the rat SSCs, so we selected this gene for further analysis along with the TF *Etv5* (which did not show differential expression between species and shows co-expression with *Gfra1* in the SSC cluster, Figure S5C). *Id4* and *Etv5* co-localized in our mouse dataset, but in the rat sc/snRNA-seq, *Etv5* and *Id4* transcripts mark distinct populations of cells (Figure 4D), suggesting that in the rat, there exist two populations of cells within the undifferentiated spermatogonia, one *Gfra1*$^{+}$*Id4*$^{+}$*Etv5*$^{-}$ population and another *Gfra1*$^{+}$*Id4*$^{-}$*Etv5*$^{+}$ population. Supporting this, cells matching these criteria were found along the basement membrane of tubules by *in situ* RNA hybridization in the rat (Figures 4E and 4F). Therefore, *Id4* has been shown to be chiefly expressed in SSCs in mouse, which is not conserved in rat, suggesting that *Id4* may have diverging functions even among closely related species.

Chromatin accessibility dynamics throughout spermatogenesis

Male germ cell maturation is accompanied by changes in chromatin accessibility (Figure 5A). Some chromatin regions, such as those containing the histone replacement protein genes *Tnp2* and *Prm1-3*, undergo dramatic remodeling across cell types and exhibit high accessibility in late round and early elongating spermatids, associated with intense transcription (Figure 5B). On a larger scale, through a comparison of chromatin structure via a genomic liftover of orthologous sequences, we found that epigenetic features are remarkably conserved between the species (Figures 5C and S6A).

We next assessed the concordance between chromatin accessibility and gene expression. We selected genes known to be important at various stages of germ cell development for both species and calculated the chromatin accessibility at gene body and promoter regions (±2 kb from the transcription start site) for each gene along its pseudotime trajectory (Figure 5D). In each case, an increase in promoter accessibility corresponded with increased gene expression. Examining the correlation of transcription and chromatin accessibility of all genes between cell types revealed that correlations are comparatively low in SSCs and spermatogonia, rising to the highest levels in early meiosis and then dropping again once the cells enter spermiogenesis (Figures 5E and S5B). Then, examining the locations of differentially accessible peaks revealed a substantial relative increase in the proportion of promoter-associated peaks in the late-differentiating spermatogonia in both species that continues until mid-meiosis (Figure 5F). After meiosis, this proportion reduced, and most differentially accessible peaks were found in intergenic and intronic sequences. Taken together, we find dramatic shifts in chromatin accessibility at the onset of meiosis together with a greater correlation of promoter accessibility to gene expression, suggesting tight epigenetic control of transcription of meiotic genes that begins in the differentiating spermatogonia.

(C) Differential gene expression between mice and rats for the undifferentiated spermatogonia across all integrated samples. Significantly different genes, including *Id4*, are colored.
(D) Expression of *Etv5* and *Id4* in the early stages of mouse and rat germ cell differentiation.
(E) *Gfra1*$^{+}$*Etv5*$^{+}$*Id4*$^{-}$ and *Gfra1*$^{+}$*Etv5*$^{-}$*Id4*$^{+}$ cells were observed on the basement membranes of rat seminiferous tubules visualized by *in situ* RNA hybridization. Dotted lines indicate estimate of cell boundaries, representative of 3 replicates. Scale bar, 10 μm.
(F) Representative tubule; scale bar, 50 μm. *Gfra1+ Etv5+ Id4−* cell denoted by white arrow.

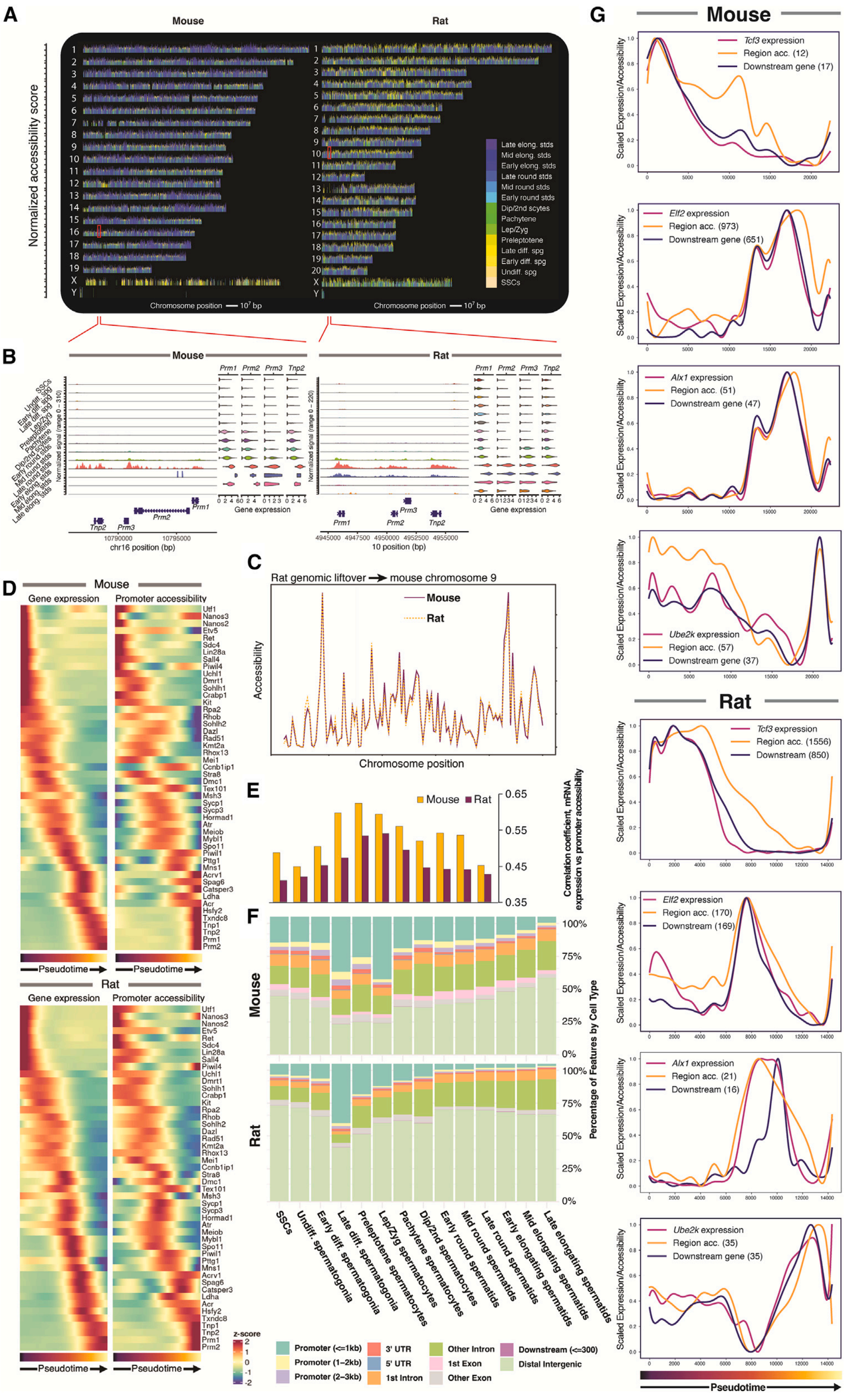

(legend on next page)

Gene regulatory network analysis reveals conserved mechanisms of transcriptional control

To further understand the mechanistic basis for spermatogenesis, we jointly explored TF interactions with chromatin and downstream gene expression. TF expression and motif accessibility displayed correlations across the breadth of cell types (Figure S5C). Using SCENIC+ (Bravo González-Blas et al., 2023) with our multiomic snRNA/ATAC-seq data, we inferred links between predicted enhancer and promoter regions along with predicted TF-gene target links within single cells. This analysis yielded a collection of enhancer-gene regulatory networks (GRNs) that integrates region co-accessibility, TF motifs, and gene expression data to yield discrete "regulons." After filtering to retain high-confidence regulons, we identified 174 distinct regulons in the mouse (113 activating regulators and 62 repressive regulators) along with 318 regulons in the rat (220 positive regulators and 99 repressive regulators). For each TF, there was a mean of 481.6 and 352.9 regions per regulon and 175.7 and 211.6 downstream genes per regulon for mouse and rat, respectively (Figures S7A and S7B; Table S4). Most positively regulated regulons show tight associations between TF expression, target region accessibility, and downstream gene expression (Figure 5G). We found that 40 regulons were shared between mouse and rat, and the majority showed strong conservation between mouse and rat when cells were ordered by pseudotime. TCF3 was activated early in spermatogenesis. In contrast, the ELF2 regulon was activated during meiosis, although in the mouse, this persisted into spermiogenesis, whereas in the rat, it showed a more pronounced decrease in activity after meiosis (Figure 5G). The ALX1 regulon showed similar late-meiotic and spermiogenic activation, although we observed a delay in gene upregulation in the rat. The UBE2K regulation also showed a similar pattern in mice and rats: early moderate activation, near-complete drop during meiosis, and then a sharp peak of TF expression, target peaks, and downstream gene expression in late round spermatids (Figure 5G). Overall, we saw strong correlations of TF expression with target region accessibility and downstream gene expression for a wide selection of regulons, underscoring the importance of tight timing of the effects of these TFs for proper progression of spermatogenesis.

Cd9 RNA and protein expression correlates with putative enhancer elements

To investigate the interplay between accessibility, transcription, and protein expression, we analyzed CD9 protein surface expression as assayed by CITE-seq, selecting CD9 as this protein is enriched on SSCs in mice and rats (Kanatsu-Shinohara et al., 2004). We found that the promoter of *Cd9* was widely accessible in pre-meiotic cells in both species (Figures 6A and 6B). This tracks broadly with mRNA and protein expression via our single-cell sequencing data. However, mRNA and protein expression were markedly higher in stem cells than in undifferentiated spermatogonia. *Cd9* expression in functional stem cells was confirmed in both species via a transplantation assay (Figures 6C and 6D). Promoter accessibility was reasonably broad in premeiotic cells and did not correlate well with gene/protein expression. Both species display an accessibility peak in the first intron of *Cd9* that significantly linked to mRNA expression, suggesting a conserved regulatory element. Additionally, in the rat, but not mouse, the presence of this peak coupled with the lack of a nearby putative repressor element peak correlated with the highest mRNA/protein expression, suggesting localization of a distal enhancer element. The downstream peak correlated more strongly with mRNA and protein expression than did the promoter region (Figures 6E–6L). *Cd9* expression was therefore better predicted by nearby putative enhancer regions than the promoter, which was broadly open in premeiotic cells.

Given the markedly higher protein expression in the stem cell compartment of the rat using CITE-seq, we next sought to confirm this finding by flow cytometry. We identified five populations of rat testicular cells using CD9

Figure 5. Chromatin accessibility in rodent spermatogenesis

(A) Differentially expressed peaks specific to individual cell types across integrated ATAC datasets (mouse $n = 6$, rat $n = 4$ in all panels in this figure). Peaks are arranged along each chromosome on the x axis, with the height of each peak proportional to the log-fold change in peak accessibility.

(B) Accessibility of chromatin for regions corresponding to red boxes in (A) that contain conserved genes associated with histone replacement. Gene expression for each gene is shown as violin plots.

(C) Gene expression for select marker genes with matching ATAC promoter activity score in both species along pseudotime trajectory.

(D) Broad-scale comparison of mouse chromosome 9 displaying average accessibility for binned genomic locations along with corresponding rat locations using genomic liftover.

(E) Pearson's correlation of gene expression and promoter activity was assessed by cell type.

(F) Breakdown of differentially expressed peak genomic locations by cell type and species.

(G) Pseudotime-ordered activity of four representative transcription factors active at different stages of spermatogenesis. For each TF, gene expression is shown along with accessibility of target regions and downstream gene expression aggregate. The number of associated chromatin peaks or genes in the corresponding regulon is shown in parentheses.

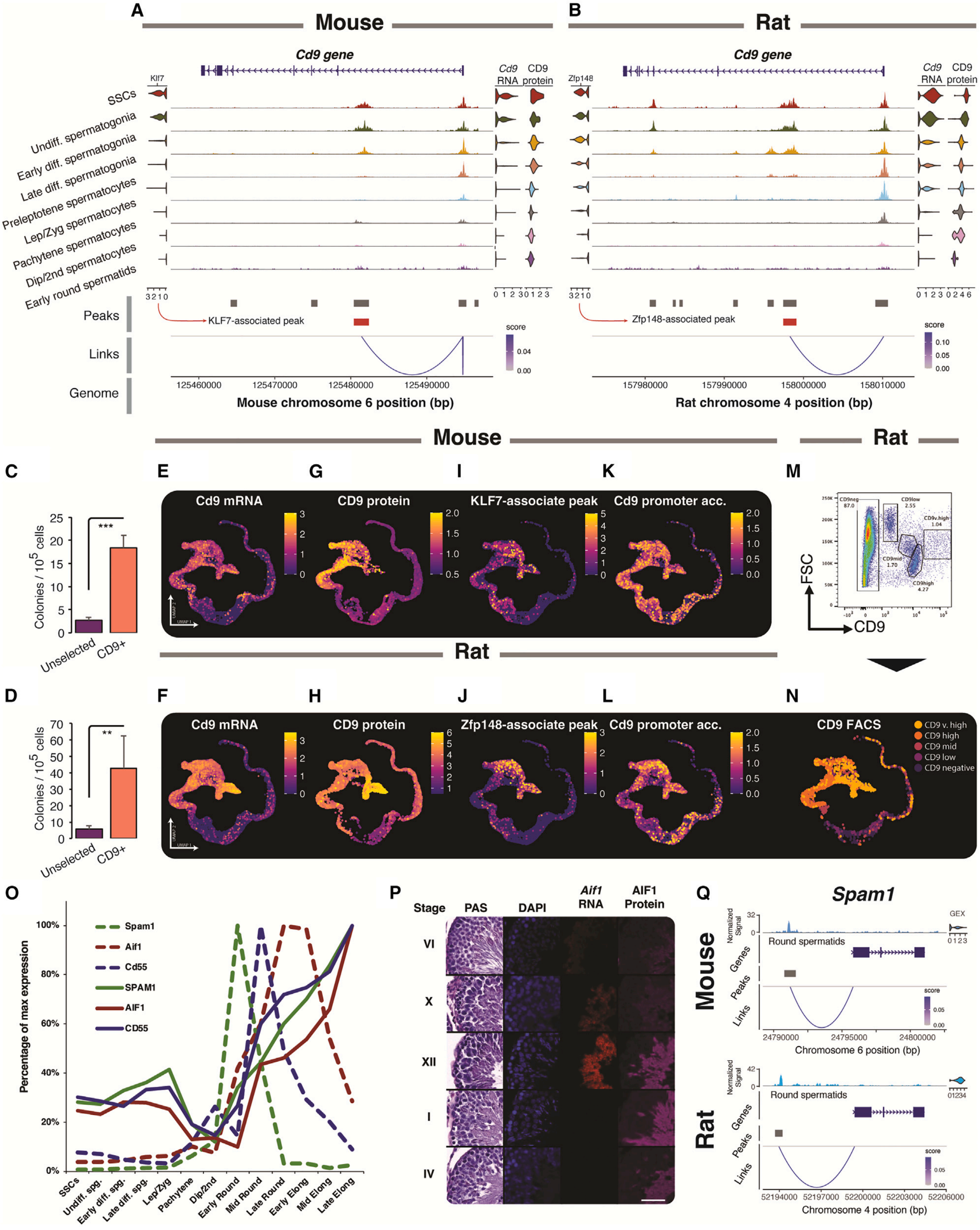

(legend on next page)

antibody staining (Figure 6M), which were then sorted based upon CD9 protein abundance via fluorescence-activated cell sorting. Each population was subjected to scRNA-seq and mapped onto the rat uniform manifold approximation and projection (UMAP) (Figure 6N). As predicted, high CD9-expressing cells mapped to undifferentiated spermatogonia, whereas CD9-negative cells were chiefly meiotic and post-meiotic cells. This result confirmed that spermatogonial populations can be sorted based on CD9 expression level alone.

We also investigated proteins involved in post-meiotic haploid germ cells in the rat, CD55, AIF1, and SPAM1. We saw evidence for delayed translation in all three, with peak mRNA expression preceding maximum protein detection (Figure 6O). For AIF1, we confirmed this via RNA *in situ* hybridization and immunofluorescence, indicating that mRNA was detected in stage X up to stage XII whereas protein was detected from stage XII elongating spermatids onward (Figure 6P), staging consistent with our single-cell observations.

Examination of peaks of accessible chromatin concomitant with gene expression within individual cells can provide evidence of sites occupied by DNA-binding proteins, which may represent key regulatory regions, especially when conserved across species. Simultaneous profiling of RNA and ATAC datasets revealed peaks, both proximal and distal to transcriptional start sites, that correlated tightly with gene expression across a range of genes that were conserved between mouse and rat (Figure S7C). For example, *Sdc4* showed a distal element downstream of the promoter site, whereas the meiotic gene *Sycp3* displayed a proximal element close to the promoter that correlated with gene expression. Other genes such as *Spam1*, expressed at the onset of spermiogenesis, indicated a strong relationship of gene expression with upstream elements (Figure 6Q). These elements are observed in both mouse and rat, consistent with their conserved regulatory roles.

Gene regulatory analysis provides a cohesive view of the conserved regulation of spermatogenesis

When examining the conserved transcriptional regulons in mice and rats, several trends are apparent. Of the 40 conserved regulons in our analysis, there is appreciable variation in the proportion of conserved downstream regions and genes (Figure 7A). Interestingly, both TF expression and their respective regulon activation were highly conserved by cell type between the species with few exceptions (Figure 7B), suggesting that while the activity of the TFs is conserved, the downstream targets may be species specific. We selected 10 regulons active at different stages of spermatogenesis and visualized their top gene target and accessible regions (Figure 7C). Even with a limited selection of TFs and downstream genes/regions, a complex progression of peak accessibility and subsequent gene expression is brought into focus. While some far-ranging connections were made, largely TF-associated peak accessibility and corresponding gene expression were linked to TFs in a cell-type-specific manner, indicating that these TFs and their regulons have limited and specific action in their respective cell types (Figure 7D). These associations can also be translated into categorizations of TF activity (Figure S7C). For example, we observed TF control of key germ cell markers such as *Sohlh1*, *Igf1r*, *Kit*, *Dmc1*, *Sycp1*, *Tex101*, *Spaca1*, *Acrv1*, *Tnp1*, and *Prm1*, indicating that these TFs may be master regulators of normal spermatogenic differentiation. In addition, genes with no known testis function such as *Mxra7*, *Snhg11*, *Pipox*, and *Ccdc27* were indicated, suggesting potential roles for these previously unreported spermiogenic genes.

DISCUSSION

Spermatogenesis is defined by three stages that are conserved across mammals: the mitotic phase, where

Figure 6. Chromatin, gene, and protein relationships

(A and B) (A) Mouse and (B) rat *Cd9* chromatin accessibility by cell type, together with *Cd9* gene expression and CD9 protein as assessed by ADT ($n = 2$ mice, $n = 2$ rats). Transcription factors *Klf7* and *Zfp148* are predicted by GRN to associate with *Cd9* in mice and rats, respectively.
(C) Transplant efficiency of CD9$^+$ mouse cells adapted from Kanatsu-Shinohara et al., 2004; $n = 16$ CD9$^+$ and $n = 13$ control.
(D) Transplant efficiency of CD9$^+$ rat cells by MACS selection, significance assessed by Student's t test, $^{**}p < 0.01$, $^{***}p < 0.001$, $n = 4$.
(E and F) UMAP projection of *Cd9* mRNA expression in mouse (E, $n = 8$) and rat (F, $n = 8$).
(G and H) CD9 protein expression in mouse (G, $n = 2$) and rat (H, $n = 2$).
(I and J) Downstream predicted enhancer accessibility in mouse (I, $n = 6$) and rat (J, $n = 4$).
(K and L) *Cd9* promoter accessibility in mouse (K, $n = 6$) and rat (L, $n = 4$).
(M) Flow cytometric analysis of CD9 expression and gates used to sort cells.
(N) Cells sorted in (M) were encapsulated and projected onto the UMAP space ($n = 1$).
(O) mRNA and protein expression for three select spermatid-associated genes normalized by percentage of max expression (mRNA, $n = 13$; CD55, $n = 6$; SPAM1, $n = 2$; AIF1, $n = 3$).
(P) Histology of PAS-stained staged sections with DAPI, *Aif1* ISH, and AIF1 immunofluorescence; scale bar = 50 μm, representative image of 3 replicates shown.
(Q) *Spam1* expression is associated with a peak upstream of the transcriptional start site in both species matched by liftover ($n = 4$).

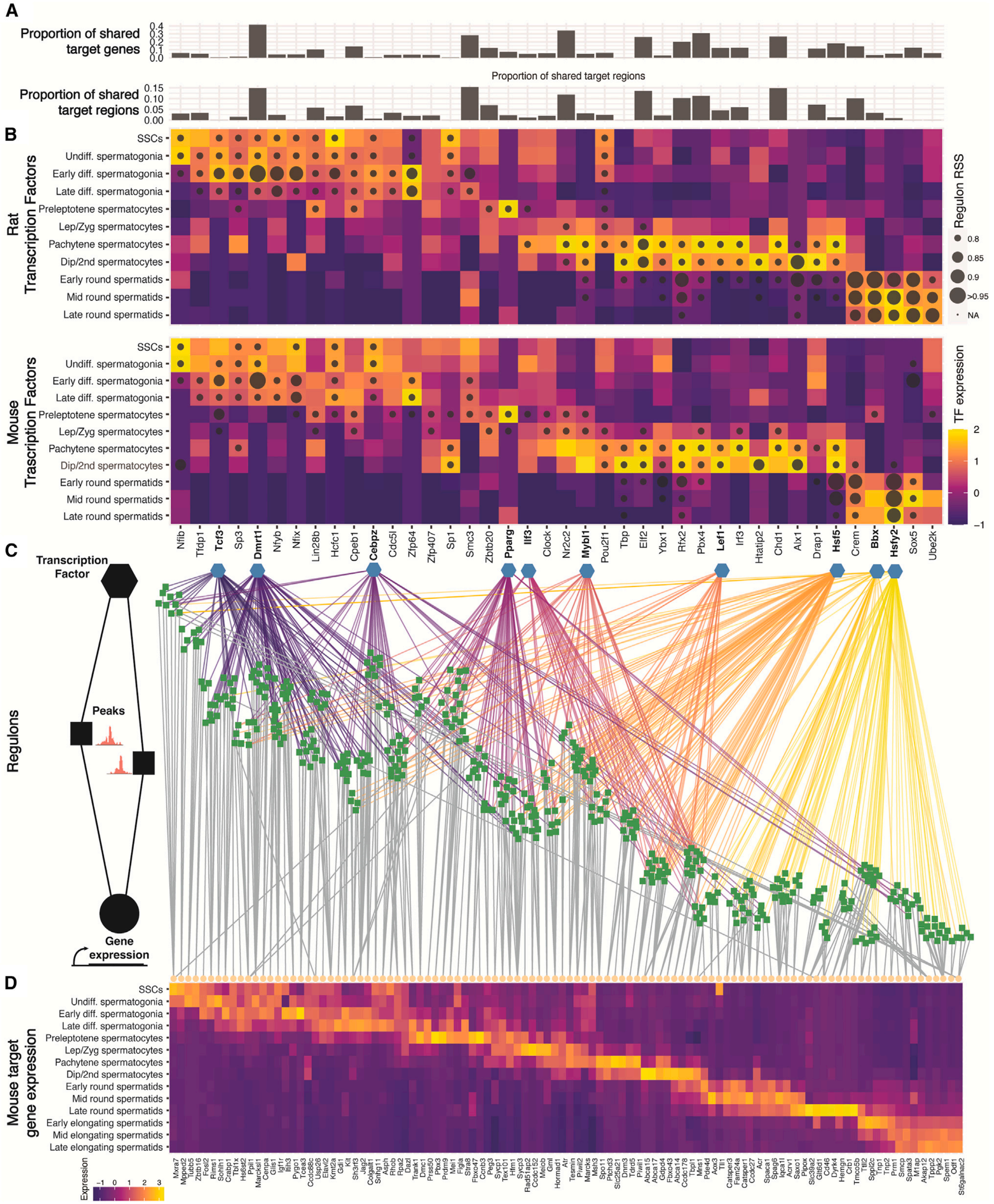

(legend on next page)

spermatogonia divide and proliferate; meiosis, during which the critical stages of chromosome crossing-over and segregation into haploid gametes occur; and finally spermiogenesis, where spermatids differentiate into functional sperm. In this study, we characterized these events at a molecular level by simultaneously comparing chromatin accessibility across the genome with transcriptomic information for individual cells and surface marker expression of a select subset of proteins. The joint RNA/ATAC multiomic analysis allowed for the identification of GRNs, in which TFs were linked via acting chromatin regions to downstream genes. Our analysis associated cell types with distinct cellular pathways. SSCs were marked by GDNF signaling, stem cell pluripotency, and MAPK signaling, consistent with established reports (Meng et al., 2000; Niu et al., 2017; Yang et al., 2021). In addition, in concordance with published data, we observed an upregulation of oxidative phosphorylation in mitotic phases (Chen et al., 2020), but not stem cells (Helsel et al., 2017), followed by a switch to glycolysis in spermiogenesis (Boussouar and Benahmed 2004), indicative of the dramatic bioenergetic shifts throughout spermatogenesis. We saw evidence for netrin signaling being important in late meiosis/early spermiogenesis, as opposed to its importance in spermatogonial differentiation that has been reported (Barroca et al., 2022). The evidence for netrin signaling's importance in late meiosis and early spermiogenesis offers a new perspective and may suggest a broader role beyond its established function in spermatogonial differentiation, potentially in guiding cellular transitions and structural organization during sperm development and influencing chromatin remodeling or cytoskeletal dynamics in late spermatogenic phases (Lai Wing Sun et al., 2011).

Mice and rats displayed remarkably similar patterns of chromatin accessibility. Notably, the correlation of gene expression with promoter accessibility was strongest during meiosis in both species, suggesting a stringent control of chromatin accessibility during meiosis. Chromatin accessibility could increase either directly as a control of expression or as a side effect of the dramatic chromatin changes during meiosis. The most substantial changes in germ cell gene accessibility were those accompanying the entry into meiosis, potentially because chromatin accessibility may control the frequency of double-stranded breaks during prophase I (Liu et al., 2022).

Our comparative multiomic analysis of mouse and rat spermiogenesis revealed a number of strong transcriptional, regulatory and chromatin similarities between mice and rats across the complex differentiation process to make male gametes. These similarities were strongest in premeiotic and meiotic cells, whereas spermatids exhibit more differences in gene and lncRNA expression, consistent with the sperm morphological differences between the two species. lncRNAs are known to have important roles in spermatogenesis. We detected stage-specific and conserved expression of known lncRNAs involved in spermatogenesis such as *Hsf2*, expressed in elongating spermatids, whose absence is associated with abnormal sperm morphology and reduced fertility and a female-biased sex ratio in offspring (Hong et al., 2021). Gene regulatory systems were also strongly conserved, from TF abundance to regulon activation in cell types, although the degree of similarity varied within regulons. Such findings suggest that despite being expressed in the same cell types, the principal downstream TF targets can be species-specific depending. While TCF3 is known as a stem cell regulatory factor (Zhou et al., 2021), our GRN analysis revealed that its effects extend into differentiating spermatogonia. TCF3 did indeed associate with a series of peaks linked to specifically stem-cell-expressed genes; other modules of downstream chromatin/gene associations were expressed in late-differentiating spermatogonia, such as TCEA3, whose function in spermatogenesis has yet to be determined. We found that *Pparg* had highly specific expression and downstream effects, limited to preleptotene spermatocytes. PPARγ forms heterodimers with the retinoid X receptor and is a crucial protein for proper spermatogenesis (Santoro et al., 2020). Our network highlighted other key TFs, such as MYB11, a master regulator of meiosis, which has been associated with a range of enhancer elements primed prior to meiosis. We found a cluster of preleptotene MYB11 enhancers as well as those acting in later spermatocytes.

Figure 7. Gene regulatory analysis of rodent spermatogenesis

(A) For each regulon, the proportion of shared target genes and target chromosomal regions are shown.

(B) Heatmap of gene expression for 40 regulons significantly associated with spermatogenesis in both mouse and rat. Color shows gene expression of the TF by cell type, dot size indicates regulon activation as indicated by regulon specificity score.

(C) For 10 selected regulons, gene regulatory network nodes are shown. Blue hexagons indicate selected TFs. Each TF's connections to target regions have been colored with a different color. Target regions are indicated as square boxes. Connections between regions and target genes (yellow circles) are indicated in gray. A subset of target regions and genes are shown, selected by taking the top 10 gene hits for each regulon in addition to any genes from Figure 2B that were found in the selected regulons.

(D) Heatmap of the gene expression of the selected genes shown by cell type.

All data in this figure were generated from snRNA/ATAC-seq multiomic profiling (n = 6 mice and 4 rats).

We also observed notable differences in expression and regulatory patterns between species, including *Zbtb16*, which is essential for SSC maintenance in mice (Buaas et al., 2004), but whose expression was much reduced in rats relative to mice in our comparison. This raises the question as to whether rat SSCs maintain stemness with less ZBTB16 protein, or if post-transcriptional mechanisms compensate, or if interactions with another protein such as SALL4 explains the difference (Lovelace et al., 2016). For example, high expression of the TF ID4 marks SSCs in mice (Helsel et al., 2017), and the TF ETV5 is necessary for pro-stem cell signaling in SSCs (Oatley et al., 2006; Yang et al., 2021) as its knockout gradually results in a Sertoli-cell-only phenotype (Morrow et al., 2007; Schlesser et al., 2008). *Id4* and *Etv5* show a very strong overlap (Hermann et al., 2018) in single-cell profiles of the mouse testis, consistent with both being markers of SSCs. However, undifferentiated spermatogonia in the rat consistently cluster into two clusters at the beginning of spermatogenesis; one cluster is marked distinctly by *Etv5* and another by *Id4*. Therefore, we conclude that, unlike in mouse, rat SSCs are not characterized by high *Id4* expression.

CD9 is a tetraspanin membrane protein that is involved in a wide range of physiological processes, including cell motility and fertilization (Hemler 2005), and is expressed in mouse and rat SSCs (Kanatsu-Shinohara et al., 2004; Hermann et al., 2018). While there is evidence that CD9 can serve as a marker of human SSCs (Zohni et al., 2012) and undifferentiated spermatogonia in goats (Kaul and Kumari, 2012), its expression is not exclusively limited to SSCs. Here, we showed that levels of CD9 protein cell surface expression varied by cell type in both rat and mice. Moreover, germ cells sorted by CD9 surface expression accurately mapped back to our atlas, indicating that CD9 protein expression alone can identify certain spermatogenic cell types.

Finally, evolutionarily conserved genes are typically involved in key biologically processes. On this basis, the conserved 40 regulons identified by our analysis are likely to play a pivotal role in spermatogenesis, ensuring a physiological progression through each maturation stage. Furthermore, lower expression or loss of function would be anticipated to result in impaired spermatogenesis and male infertility, although this will require further experimental validation. However, previous work has demonstrated that TCF3 deficiency is associated with spermatogenesis failure in infertile human patients (Zhou et al., 2021). Similarly, DMRT1 loss of function has been demonstrated in infertile men (Zarkower and Murphy 2022) while PPARG plays a role in testis fatty acid dysmetabolism in men with impaired spermatogenesis (Olia Bagheri et al., 2021). These findings strongly support the hypothesis that our core of conserved regulons includes essential genes for the preservation of normal spermatogenesis. As such, they represent candidates for the establishment of diagnostic molecular tests as well as targeted strategies for the treatment of male infertility.

METHODS

Animal use

All animal protocols were approved by University of Pennsylvania Institutional Animal Care and Use Committee (protocol number 800375).

Tissue isolation

Rat cells were isolated from the testes of 3- to 4-month-old Sprague-Dawley rats transgenic for the LacZ gene under the metallothionein promoter (Clouthier et al., 1996). Mouse cells were isolated from C57BL/6 inbred mice also 3–4 months old. Tissue was chopped into fine pieces and incubated in collagenase (Sigma) at a concentration of 1 mg/mL in HBSS (Gibco) for 15 min at 37°C. Cells were spun down for 1 min at 600 *rcf*, then resuspended in warm Trypsin (Gibco, 0.25%) with 20% DNase solution (Sigma, 7 mg/mL dissolved in HBSS). Tissue was pipetted for 2 min with a 10 mL pipette and incubated at 37°C for 5 min. Then tissue was pipetted for another 2 min and incubated at 37°C for 3 min. Fetal bovine serum (FBS) (Sigma F2442) was added to stop the digestion. Additional DNase was added until no turbidity was visible. Cells were washed in PBS-S twice (PBS [Gibco] with 1% FBS, 10 mM HEPES [Sigma-Aldrich], 1 mg/mL glucose [Sigma-Aldrich], 1 mM pyruvate [Gibco], 50 units/mL penicillin [Gibco], and 50 μg/mL streptomycin [Gibco] prepared as described in the study by Kubota and Brinster (2008)). All centrifugation steps were 5 min at 600 *rcf*.

Cells were processed both as unselected samples and enriched for EpCAM as described further. Each biological replicate was then used for either encapsulation of cells with antibody treatment or further processed as nuclei for GEX+ATAC assay.

Single-cell multiomic sequencing

Cells were encapsulated and libraries generated for CITE-seq using a Chromium Next GEM Single Cells 3′ Kit v.3.1 with Feature Barcoding (10X Genomics) per manufacturer's protocol. Prepared nuclei were encapsulated with the Chromium Next GEM Single-Cell Multiome ATAC + Gene Expression kit following manufacturer's instructions. Each biological replicate was encapsulated individually. Libraries were sequenced on a NextSeq2000 sequencer (Illumina) using a 100-cycle sequencing kits to a minimum depth of 30k reads per cell. CellRanger v.7.0.0 was used to

align reads to the *Mus musculus* reference (GENCODE vM23/Ensembl 98) and the *Rattus norvegicus* 6.0 DNA primary assembly along with the corresponding GTF file (v.6.0.85) from Ensembl (Yates et al., 2020).

Single-cell multiomic data processing and integration

Gene counts were analyzed with Seurat v.4 (Butler et al., 2018) for clustering, integration, and differential gene expression and Monocle version 3 (Qiu et al., 2017) for clustering and pseudotime. Filtering cutoffs were set as follows: minimum genes per cell = 500, maximum genes = 5,000, and minimum unique molecular identifiers (UMIs) per cell were set on a per-sample basis after inspecting a rank plot of UMI/cell. For cells with ATAC data, filtering criteria of minimum fragments per cell of 100 and maximum of 10,000 were also applied. Cells with over 20% mitochondrial reads were also excluded. The RNA assay was processed using Seurat's NormalizeData and ScaleData using 2,000 variable features before being integrated using Seurat's FindIntegrationAnchors and IntegrateData functions with default parameters. The first 30 dimensions were used for integration of the RNA assay of all samples (regardless of associated modality) and joint UMAP creation. Mouse and rat samples were integrated as mentioned earlier, ensuring all variable features were matched by name to homologous genes. Clusters were found using Monocle (cluster_cells, resolution = 5×10^{-5}, k = 8, partition_qval = 0.05). Differentially expressed genes were identified via Seurat's FindMarkers function with default parameters. Pseudotime was generated using Monocle's learn_graph (default parameters). TE element analysis used SoloTE (Rodríguez-Quiroz and Valdebenito-Maturana 2022) on the RNA assay of mouse and rat datasets using default parameters and normalized using Seurat's default normalization method on a combined assay involving all genes and TEs.

For the scRNA-seq + ATAC-seq samples, the ATAC modality was processed independently of the RNA assay. Peaks were called using Signac (Stuart et al., 2021) with MACS2, and gene activity scores were calculated with Signac's GeneActivity function. First, a shared peak list was created by merging all samples and then that shared peak list was used to create Seurat objects for each sample individually. FindTopFeatures with min.cutoff = 10, RunTFIDF, and RunSVD were run. Samples were integrated using FindIntegrationAnchors, reduction = "rlsi," and 2:30 dimensions. Then LSI embeddings were integrated using 1:30 dimensions. Finally, the integrated ATAC assay was joined to the integrated RNA assay by cell name. For linking peaks to genes, Signac's LinkPeaks function was used with default parameters to link ATAC and RNA assays.

To compare nascent versus all mRNA, spliced, unspliced, and ambiguous counts were determined using Velocyto (La et al., 2018). Then, using only unspliced counts, the whole process of integration and cell type assignments and UMAP projection was performed as detailed earlier, after which the correlation of original and unspliced-only cell type designations was correlated by cell types.

For CD9-sorted cells, each sorted fraction was included in the main integration with all other cells and their identities were retained. The CD9-sorted cells were subset and projected onto the same UMAP.

RESOURCE AVAILABILITY

Lead contact

Requests for further information and resources should be directed to and will be fulfilled by the lead contact, Eoin C. Whelan (ewhelan@vet.upenn.edu).

Materials availability

This study did not generate new unique reagents.

Data and code availability

Single-cell RNA/ADT/ATAC data have been deposited at NCBI GEO at GEO accession number GSE268104 and are publicly available as of the date of publication. All original code is available in this paper's supplemental information.

ACKNOWLEDGMENTS

We thank Kotaro Sasaki, Keren Cheng, Leslie King, and Andrew Modzelewski for helpful advice. We also thank C. Freeman, R. Naroznowski, and D. Lee for animal maintenance. We are grateful for the support of Robert J. Kleberg, Jr and Helen C. Kleberg Foundation (RLB). https://www.klebergfoundation.org/grant-guidelines/medical-research/. Additionally, the Penn Vet Comparative Pathology Core receives funding from the Abrahamson Cancer Center Support Grant (P30 CA016520), the Aperio Versa 200 scanner used for imaging was acquired through an NIH Shared Instrumentation Grant (S10 OD023465-01A1), and the Leica BOND RXm instrument used for IHC and ISH was acquired through the Penn Vet IIZD Core pilot grant opportunity 2022.

AUTHOR CONTRIBUTIONS

Conceptualization, E.C.W. and R.L.B.; methodology, E.C.W., D.P.B., R.L.B., J.H.S., and D.S.; investigation, E.C.W., J.J.S., F.Y., M.R.A., A.R., C.M., E.R., J.H.S., and D.S.; visualization, E.C.W., J.H.S., and D.S.; funding acquisition, R.L.B.; project supervision, R.L.B.; writing – original draft, E.C.W.; writing – review and editing, E.C.W., F.Y., A.R., D.P.B., J.H.S., and R.L.B.

DECLARATION OF INTERESTS

The authors declare no competing interests.

SUPPLEMENTAL INFORMATION

Supplemental information can be found online at https://doi.org/10.1016/j.stemcr.2025.102449.

Received: May 23, 2024
Revised: February 12, 2025
Accepted: February 13, 2025
Published: March 13, 2025

REFERENCES

Barroca, V., Racine, C., Pays, L., Fouchet, P., Coureuil, M., and Allemand, I. (2022). The netrin-1 receptor UNC5C contributes to the homeostasis of undifferentiated spermatogonia in adult mice. Stem Cell Res. *60*, 102723.

Boussouar, F., and Benahmed, M. (2004). Lactate and energy metabolism in male germ cells. Trends Endocrinol. Metabol. *15*, 345–350.

Bravo González-Blas, C., De Winter, S., Hulselmans, G., Hecker, N., Matetovici, I., Christiaens, V., Poovathingal, S., Wouters, J., Aibar, S., and Aerts, S. (2023). SCENIC+: single-cell multiomic inference of enhancers and gene regulatory networks. Nat. Methods *20*, 1355–1367.

Buaas, F.W., Kirsh, A.L., Sharma, M., McLean, D.J., Morris, J.L., Griswold, M.D., de Rooij, D.G., and Braun, R.E. (2004). Plzf is required in adult male germ cells for stem cell self-renewal. Nat. Genet. *36*, 647–652.

Butler, A., Hoffman, P., Smibert, P., Papalexi, E., and Satija, R. (2018). Integrating single-cell transcriptomic data across different conditions, technologies, and species. Nat. Biotechnol. *36*, 411–420.

Chen, W., Zhang, Z., Chang, C., Yang, Z., Wang, P., Fu, H., Wei, X., Chen, E., Tan, S., Huang, W., et al. (2020). A bioenergetic shift is required for spermatogonial differentiation. Cell Discov. *6*, 56.

Clouthier, D.E., Avarbock, M.R., Maika, S.D., Hammer, R.E., and Brinster, R.L. (1996). Rat spermatogenesis in mouse testis. Nature *381*, 418–421.

Corzett, M., Mazrimas, J., and Balhorn, R. (2002). Protamine 1: protamine 2 stoichiometry in the sperm of eutherian mammals. Mol. Reprod. Dev. *61*, 519–527.

Ernst, C., Eling, N., Martinez-Jimenez, C.P., Marioni, J.C., and Odom, D.T. (2019). Staged developmental mapping and X chromosome transcriptional dynamics during mouse spermatogenesis. Nat. Commun. *10*, 1251.

Gewiss, R.L., Shelden, E.A., and Griswold, M.D. (2021). STRA8 induces transcriptional changes in germ cells during spermatogonial development. Mol. Reprod. Dev. *88*, 128–140.

Grasso, M., Fuso, A., Dovere, L., de Rooij, D.G., Stefanini, M., Boitani, C., and Vicini, E. (2012). Distribution of GFRA1-expressing spermatogonia in adult mouse testis. Reproduction *143*, 325–332.

Green, C.D., Ma, Q., Manske, G.L., Shami, A.N., Zheng, X., Marini, S., Moritz, L., Sultan, C., Gurczynski, S.J., Moore, B.B., et al. (2018). A Comprehensive Roadmap of Murine Spermatogenesis Defined by Single-Cell RNA-Seq. Dev. Cell *46*, 651–667.e10.

Grive, K.J., Hu, Y., Shu, E., Grimson, A., Elemento, O., Grenier, J.K., and Cohen, P.E. (2019). Dynamic transcriptome profiles within spermatogonial and spermatocyte populations during postnatal testis maturation revealed by single-cell sequencing. PLoS Genet. *15*, e1007810.

Guo, J., Grow, E.J., Mlcochova, H., Maher, G.J., Lindskog, C., Nie, X., Guo, Y., Takei, Y., Yun, J., Cai, L., et al. (2018). The adult human testis transcriptional cell atlas. Cell Res. *28*, 1141–1157.

Helsel, A.R., Oatley, M.J., and Oatley, J.M. (2017). Glycolysis-Optimized Conditions Enhance Maintenance of Regenerative Integrity in Mouse Spermatogonial Stem Cells during Long-Term Culture. Stem Cell Rep. *8*, 1430–1441.

Helsel, A.R., Yang, Q.E., Oatley, M.J., Lord, T., Sablitzky, F., and Oatley, J.M. (2017). ID4 levels dictate the stem cell state in mouse spermatogonia. Development *144*, 624–634.

Hemler, M.E. (2005). Tetraspanin functions and associated microdomains. Nat. Rev. Mol. Cell Biol. *6*, 801–811.

Hermann, B.P., Cheng, K., Singh, A., Roa-De La Cruz, L., Mutoji, K.N., Chen, I.C., Gildersleeve, H., Lehle, J.D., Mayo, M., Westernströer, B., et al. (2018). The Mammalian Spermatogenesis Single-Cell Transcriptome, from Spermatogonial Stem Cells to Spermatids. Cell Rep. *25*, 1650–1667.e8.

Hong, S.H., Han, G., Lee, S.J., Cocquet, J., and Cho, C. (2021). Testicular germ cell–specific lncRNA, Teshl, is required for complete expression of Y chromosome genes and a normal offspring sex ratio. Sci. Adv. *7*, eabg5177.

Huang, L., Zhang, J., Zhang, P., Huang, X., Yang, W., Liu, R., Sun, Q., Lu, Y., Zhang, M., and Fu, Q. (2023). Single-cell RNA sequencing uncovers dynamic roadmap and cell-cell communication during buffalo spermatogenesis. iScience *26*, 105733.

Ishii, K., Kanatsu-Shinohara, M., Toyokuni, S., and Shinohara, T. (2012). FGF2 mediates mouse spermatogonial stem cell self-renewal via upregulation of Etv5 and Bcl6b through MAP2K1 activation. Development *139*, 1734–1743.

Jung, M., Wells, D., Rusch, J., Ahmad, S., Marchini, J., Myers, S.R., and Conrad, D.F. (2019). Unified single-cell analysis of testis gene regulation and pathology in five mouse strains. Elife *8*, e43966.

Kanatsu-Shinohara, M., Toyokuni, S., and Shinohara, T. (2004). CD9 is a surface marker on mouse and rat male germline stem cells. Biol. Reprod. *70*, 70–75.

Kaul, G., and Kumari, S. (2012). Enrichment of CD9+ spermatogonial stem cells from goat (Capra aegagrus hircus) testis using magnetic microbeads. Stem Cell Discov. *2*, 92–99.

Koubova, J., Menke, D.B., Zhou, Q., Capel, B., Griswold, M.D., and Page, D.C. (2006). Retinoic acid regulates sex-specific timing of meiotic initiation in mice. Proc. Natl. Acad. Sci. USA *103*, 2474–2479.

Kubota, H., and Brinster, R.L. (2008). Culture of rodent spermatogonial stem cells, male germline stem cells of the postnatal animal. Methods Cell Biol. *86*, 59–84.

La, H.M., Mäkelä, J.A., Chan, A.L., Rossello, F.J., Nefzger, C.M., Legrand, J.M.D., De Seram, M., Polo, J.M., and Hobbs, R.M. (2018). Identification of dynamic undifferentiated cell states within the male germline. Nat. Commun. *9*, 2819.

La Manno, G., Soldatov, R., Zeisel, A., Braun, E., Hochgerner, H., Petukhov, V., Lidschreiber, K., Kastriti, M.E., Lönnerberg, P., Furlan, A., et al. (2018). RNA velocity of single cells. Nature *560*, 494–498.

Lai Wing Sun, K., Correia, J.P., and Kennedy, T.E. (2011). Netrins: versatile extracellular cues with diverse functions. Development *138*, 2153–2169.

Lau, X., Munusamy, P., Ng, M.J., and Sangrithi, M. (2020). Single-Cell RNA Sequencing of the Cynomolgus Macaque Testis Reveals Conserved Transcriptional Profiles during Mammalian Spermatogenesis. Dev. Cell *54*, 548–566.e7.

Li, S., Yan, R.G., Gao, X., He, Z., Wu, S.X., Wang, Y.J., Zhang, Y.W., Tao, H.P., Zhang, X.N., Jia, G.X., and Yang, Q.E. (2024). Single-cell transcriptome analyses reveal critical regulators of spermatogonial stem cell fate transitions. BMC Genom. *25*, 138.

Liu, G., Sun, Y., Jia, L., Li, R., and Zuo, Y. (2022). Chromatin accessibility shapes meiotic recombination in mouse primordial germ cells through assisting double-strand breaks and loop formation. Biochim. Biophys. Acta. Gene Regul. Mech. *1865*, 194844.

Lovelace, D.L., Gao, Z., Mutoji, K., Song, Y.C., Ruan, J., and Hermann, B.P. (2016). The regulatory repertoire of PLZF and SALL4 in undifferentiated spermatogonia. Development *143*, 1893–1906.

Lukassen, S., Bosch, E., Ekici, A.B., and Winterpacht, A. (2018). Characterization of germ cell differentiation in the male mouse through single-cell RNA sequencing. Sci. Rep. *8*, 6521.

Meng, X., Lindahl, M., Hyvönen, M.E., Parvinen, M., de Rooij, D.G., Hess, M.W., Raatikainen-Ahokas, A., Sainio, K., Rauvala, H., Lakso, M., et al. (2000). Regulation of cell fate decision of undifferentiated spermatogonia by GDNF. Science *287*, 1489–1493.

Mipam, T., Chen, X., Zhao, W., Zhang, P., Chai, Z., Yue, B., Luo, H., Wang, J., Wang, H., Wu, Z., et al. (2023). Single-cell transcriptome analysis and in vitro differentiation of testicular cells reveal novel insights into male sterility of the interspecific hybrid cattle-yak. BMC Genom. *24*, 149.

Morrow, C.M.K., Hostetler, C.E., Griswold, M.D., Hofmann, M.C., Murphy, K.M., Cooke, P.S., and Hess, R.A. (2007). ETV5 is required for continuous spermatogenesis in adult mice and may mediate blood testes barrier function and testicular immune privilege. Ann. N. Y. Acad. Sci. *1120*, 144–151.

Niu, Z., Mu, H., Zhu, H., Wu, J., and Hua, J. (2017). p38 MAPK pathway is essential for self-renewal of mouse male germline stem cells (mGSCs). Cell Prolif. *50*, e12314.

Oatley, J.M., Avarbock, M.R., Telaranta, A.I., Fearon, D.T., and Brinster, R.L. (2006). Identifying genes important for spermatogonial stem cell self-renewal and survival. Proc. Natl. Acad. Sci. USA *103*, 9524–9529.

Olia Bagheri, F., Alizadeh, A., Sadighi Gilani, M.A., and Shahhoseini, M. (2021). Role of peroxisome proliferator-activated receptor gamma (PPARγ) in the regulation of fatty acid metabolism related gene expressions in testis of men with impaired spermatogenesis. Reprod. Biol. *21*, 100543.

Qiu, X., Mao, Q., Tang, Y., Wang, L., Chawla, R., Pliner, H.A., and Trapnell, C. (2017). Reversed graph embedding resolves complex single-cell trajectories. Nat. Methods *14*, 979–982.

Rodríguez-Quiroz, R., and Valdebenito-Maturana, B. (2022). SoloTE for improved analysis of transposable elements in single-cell RNA-Seq data using locus-specific expression. Commun. Biol. *5*, 1063.

Russell, L.D., Ettlin, R.A., Hikim, A.P.S., and Clegg, E.D. (1993). Histological and Histopathological Evaluation of the Testis (Cache River Press), pp. 62–118.

Ryu, B.Y., Kubota, H., Avarbock, M.R., and Brinster, R.L. (2005). Conservation of spermatogonial stem cell self-renewal signaling between mouse and rat. Proc. Natl. Acad. Sci. USA *102*, 14302–14307.

Salehi, N., and Totonchi, M. (2023). The construction of a testis transcriptional cell atlas from embryo to adult reveals various somatic cells and their molecular roles. J. Transl. Med. *21*, 859.

Santoro, M., De Amicis, F., Aquila, S., and Bonofiglio, D. (2020). Peroxisome proliferator-activated receptor gamma expression along the male genital system and its role in male fertility. Hum. Reprod. *35*, 2072–2085.

Schlesser, H.N., Simon, L., Hofmann, M.C., Murphy, K.M., Murphy, T., Hess, R.A., and Cooke, P.S. (2008). Effects of ETV5 (ets variant gene 5) on testis and body growth, time course of spermatogonial stem cell loss, and fertility in mice. Biol. Reprod. *78*, 483–489.

Shami, A.N., Zheng, X., Munyoki, S.K., Ma, Q., Manske, G.L., Green, C.D., Sukhwani, M., Orwig, K.E., Li, J.Z., and Hammoud, S.S. (2020). Single-Cell RNA Sequencing of Human, Macaque, and Mouse Testes Uncovers Conserved and Divergent Features of Mammalian Spermatogenesis. Dev. Cell *54*, 529–547.e12.

Sinha, N., Whelan, E.C., Tobias, J.W., Avarbock, M., Stefanovski, D., and Brinster, R.L. (2021). Roles of Stra8 and Tcerg1l in retinoic acid induced spermatogonial differentiation in mouse. Biol. Reprod. *105*, 503–518.

Sohni, A., Tan, K., Song, H.W., Burow, D., de Rooij, D.G., Laurent, L., Hsieh, T.C., Rabah, R., Hammoud, S.S., Vicini, E., and Wilkinson, M.F. (2019). The Neonatal and Adult Human Testis Defined at the Single-Cell Level. Cell Rep. *26*, 1501–1517.e4.

Stuart, T., Srivastava, A., Madad, S., Lareau, C.A., and Satija, R. (2021). Single-cell chromatin state analysis with Signac. Nat. Methods *18*, 1333–1341.

Tegelenbosch, R.A., and de Rooij, D.G. (1993). A quantitative study of spermatogonial multiplication and stem cell renewal in the C3H/101 F1 hybrid mouse. Mutat. Res. *290*, 193–200.

Wang, M., Liu, X., Chang, G., Chen, Y., An, G., Yan, L., Gao, S., Xu, Y., Cui, Y., Dong, J., et al. (2018). Single-Cell RNA Sequencing Analysis Reveals Sequential Cell Fate Transition during Human Spermatogenesis. Cell Stem Cell *23*, 599–614.e4.

Wang, R.S., Yeh, S., Tzeng, C.R., and Chang, C. (2009). Androgen receptor roles in spermatogenesis and fertility: lessons from testicular cell-specific androgen receptor knockout mice. Endocr. Rev. *30*, 119–132.

Wang, X., Pei, J., Xiong, L., Guo, S., Cao, M., Kang, Y., Ding, Z., La, Y., Liang, C., Yan, P., and Guo, X. (2023). Single-Cell RNA Sequencing Reveals Atlas of Yak Testis Cells. Int. J. Mol. Sci. *24*, 7982.

Whelan, E.C., Yang, F., Avarbock, M.R., Sullivan, M.C., Beiting, D.P., and Brinster, R.L. (2022). Reestablishment of spermatogenesis after more than 20 years of cryopreservation of rat spermatogonial stem cells reveals an important impact in differentiation capacity. PLoS Biol. *20*, e3001618.

Wu, X., Lu, M., Yun, D., Gao, S., Chen, S., Hu, L., Wu, Y., Wang, X., Duan, E., Cheng, C.Y., and Sun, F. (2022a). Single-cell ATAC-Seq reveals cell type-specific transcriptional regulation and unique

chromatin accessibility in human spermatogenesis. Hum. Mol. Genet. *31*, 321–333.

Wu, X., Lu, M., Yun, D., Gao, S., and Sun, F. (2024). Long-read single-cell sequencing reveals the transcriptional landscape of spermatogenesis in obstructive azoospermia and sertoli cell-only patients. QJM *117*, 422–435.

Wu, Y., Guo, T., Li, J., Niu, C., Sun, W., Zhu, S., Zhao, H., Qiao, G., Han, M., He, X., et al. (2022b). The Transcriptional Cell Atlas of Testis Development in Sheep at Pre-Sexual Maturity. Curr. Issues Mol. Biol. *44*, 483–497.

Yang, F., Whelan, E.C., Guan, X., Deng, B., Wang, S., Sun, J., Avarbock, M.R., Wu, X., and Brinster, R.L. (2021). FGF9 promotes mouse spermatogonial stem cell proliferation mediated by p38 MAPK signalling. Cell Prolif. *54*, e12933.

Yang, H., Ma, J., Wan, Z., Wang, Q., Wang, Z., Zhao, J., Wang, F., and Zhang, Y. (2021). Characterization of sheep spermatogenesis through single-cell RNA sequencing. FASEB J. *35*, e21187.

Yates, A.D., Achuthan, P., Akanni, W., Allen, J., Allen, J., Alvarez-Jarreta, J., Amode, M.R., Armean, I.M., Azov, A.G., Bennett, R., et al. (2020). Ensembl 2020. Nucleic Acids Res. *48*, D682–D688.

Yu, X.W., Li, T.T., Du, X.M., Shen, Q.Y., Zhang, M.F., Wei, Y.D., Yang, D.H., Xu, W.J., Chen, W.B., Bai, C.L., et al. (2021). Single-cell RNA sequencing reveals atlas of dairy goat testis cells. Zool. Res. *42*, 401–405.

Zarkower, D., and Murphy, M.W. (2022). DMRT1: An Ancient Sexual Regulator Required for Human Gonadogenesis. Sex. Dev. *16*, 112–125.

Zhang, H., Situ, C., and Guo, X. (2022a). Recent progress of proteomic analysis on spermatogenesis. Biol. Reprod. *107*, 109–117.

Zhang, L., Guo, M., Liu, Z., Liu, R., Zheng, Y., Yu, T., Lv, Y., Lu, H., Zeng, W., Zhang, T., and Pan, C. (2022b). Single-cell RNA-seq analysis of testicular somatic cell development in pigs. J. Genet. Genomics *49*, 1016–1028.

Zhang, L., Li, F., Lei, P., Guo, M., Liu, R., Wang, L., Yu, T., Lv, Y., Zhang, T., Zeng, W., et al. (2021). Single-cell RNA-sequencing reveals the dynamic process and novel markers in porcine spermatogenesis. J. Anim. Sci. Biotechnol. *12*, 122.

Zheng, Y., Liu, Y., Hou, R., Shi, K., Chen, Y., Feng, T., and An, J. (2022). Single-cell RNA-sequencing analysis and characterisation of testicular cells in giant panda (Ailuropoda melanoleuca). Reprod. Fertil. Dev. *34*, 933–943.

Zhou, D., Fan, J., Liu, Z., Tang, R., Wang, X., Bo, H., Zhu, F., Zhao, X., Huang, Z., Xing, L., et al. (2021). TCF3 Regulates the Proliferation and Apoptosis of Human Spermatogonial Stem Cells by Targeting PODXL. Front. Cell Dev. Biol. *9*, 695545.

Zohni, K., Zhang, X., Tan, S.L., Chan, P., and Nagano, M. (2012). CD9 is expressed on human male germ cells that have a long-term repopulation potential after transplantation into mouse testes. Biol. Reprod. *87*, 27.

Stem Cell Reports, Volume *20*

Supplemental Information

Single-cell multiomic comparison of mouse and rat spermatogenesis reveals gene regulatory networks conserved for over 20 million years

Eoin C. Whelan, John J. Swain, Jonathan H. Sussman, David Smith, Fan Yang, Antonia Rotolo, Mary R. Avarbock, Clara Malekshahi, Enrico Radaelli, Daniel P. Beiting, and Ralph L. Brinster

SUPPLEMENTARY FIGURES

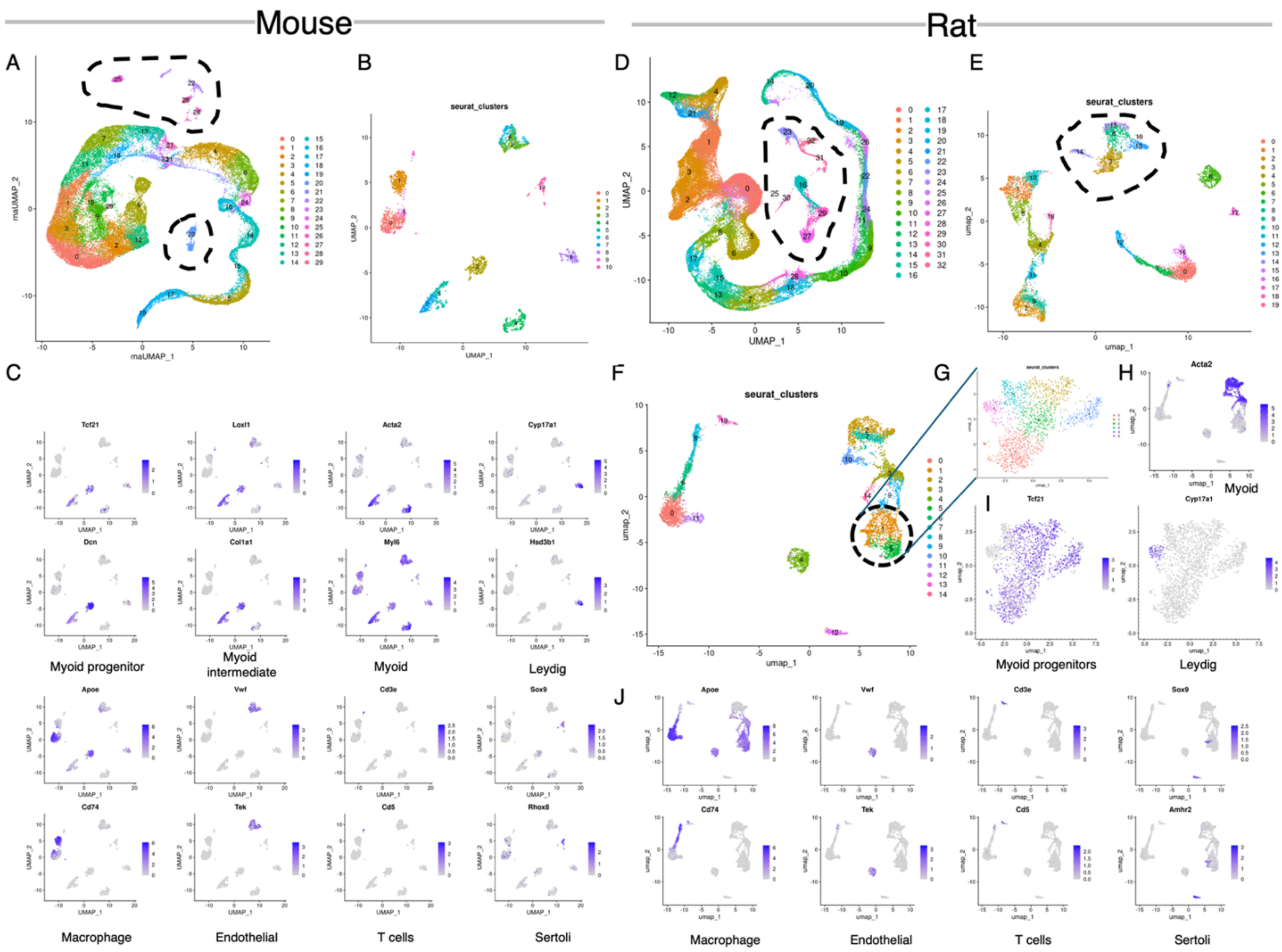
Mouse
Rat
A
B
seurat_clusters
C
D
E
seurat_clusters
F
seurat_clusters
G
H
Acta2
Myoid
I
Tcf21
Cyp17a1
Tcf21
Loxl1
Acta2
Cyp17a1
Dcn
Col1a1
Myl6
Hsd3b1
Myoid progenitor
Myoid intermediate
Myoid
Leydig
Myoid progenitors
Leydig
J
Apoe
Vwf
Cd3e
Sox9
Cd74
Tek
Cd5
Rhox8
Amhr2
Macrophage
Endothelial
T cells
Sertoli

Figure S1. Somatic cells in the mouse and rat. (A) Clustering of all mouse testicular cells. Clusters within dotted lines display somatic markers and were subclustered for somatic analysis. All other cells were used in the germ cell analysis. (B) Resulting somatic clusters after subclustering. (C) *Tcf21+* Myoid progenitors, intermediate cells and their progeny, myoid and Leydig cells were discrete clusters in mouse. Macrophages, endothelial cells, T cells and Sertoli cells were also apparent and representative gene expression is shown for each of these clusters. (D) Clustering of all rat testicular cells. Clusters within dotted lines display somatic markers and were subclustered for somatic analysis. All other cells were used in the germ cell analysis. (E) Subclustered cells retained some germ cell clusters shown in the dotted lines, which were removed and reclustered again. (F) Resulting somatic clusters after the second subclustering. Myoid progenitor cells were difficult to resolve in rat (unlike mouse) from Leydig cells. (G) After another round of subclustering of the myoid progenitors/Leydig cluster, (H) both cell types were distinct as shown by *Tcf21* (progenitors) and *Cyp17a1* (Leydig cells). (I) Myoid cells show progression from progenitors in rat. (J) Markers of immune, endothelial and Sertoli cells. All data in this figure were generated from sn/scRNA-seq (n = 8 mice and 13 rats).

4

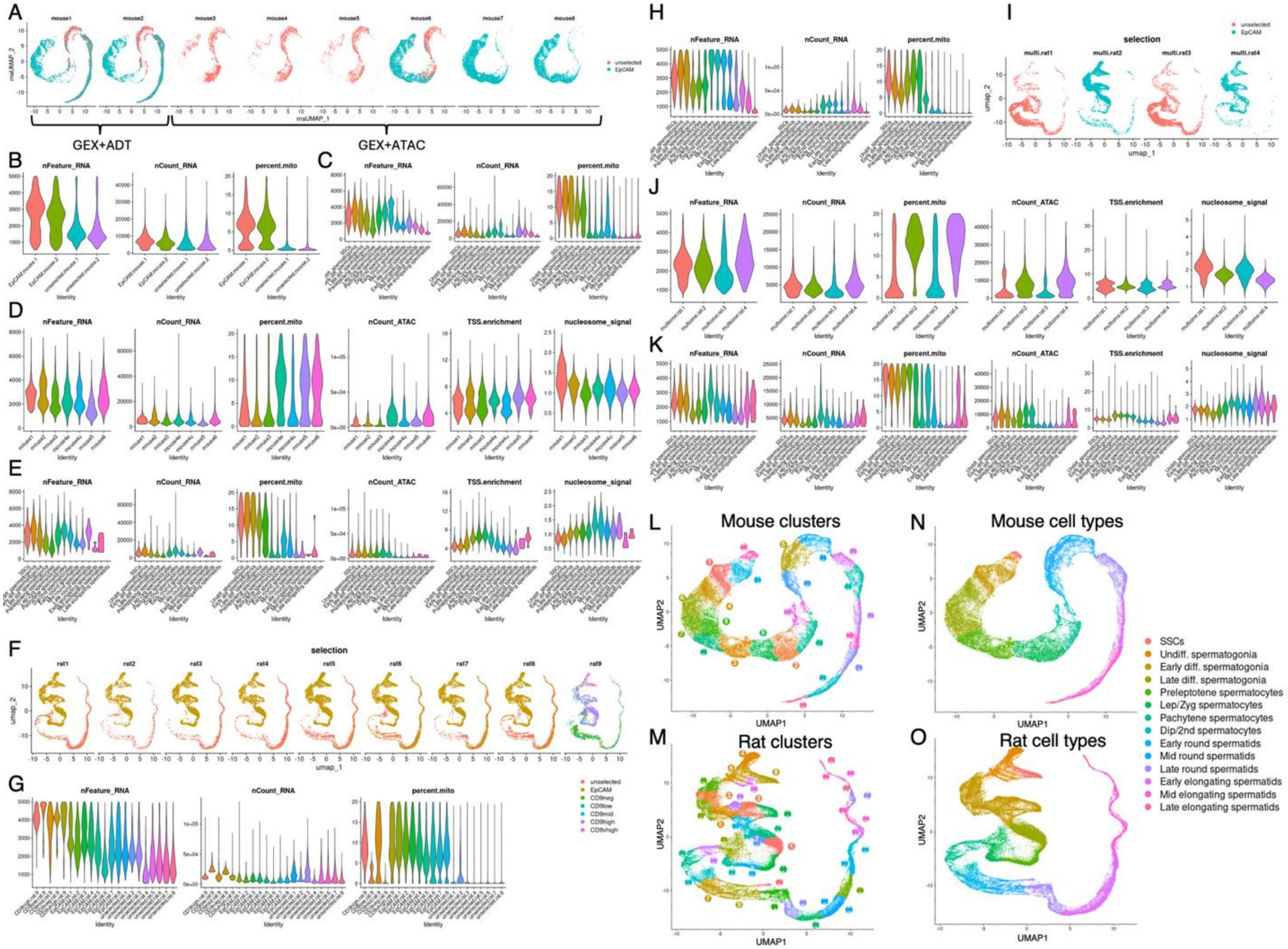
A
mouse1
mouse2
mouse3
mouse4
mouse5
mouse6
mouse7
mouse8
unselected
EpCAM
rnaUMAP_2
rnaUMAP_1
GEX+ADT
GEX+ATAC
B
C
D
E
nFeature_RNA
nCount_RNA
percent.mito
nCount_ATAC
TSS.enrichment
nucleosome_signal
Identity
F
selection
rat1
rat2
rat3
rat4
rat5
rat6
rat7
rat8
rat9
umap_2
umap_1
G
unselected
EpCAM
CD9neg
CD9low
CD9mid
CD9high
CD9vhigh
H
I
multi.rat1
multi.rat2
multi.rat3
multi.rat4
J
K
L
Mouse clusters
M
Rat clusters
N
Mouse cell types
O
Rat cell types
UMAP1
UMAP2
SSCs
Undiff. spermatogonia
Early diff. spermatogonia
Late diff. spermatogonia
Preleptotene spermatocytes
Lep/Zyg spermatocytes
Pachytene spermatocytes
Dip/2nd spermatocytes
Early round spermatids
Mid round spermatids
Late round spermatids
Early elongating spermatids
Mid elongating spermatids
Late elongating spermatids

Figure S2. Quality metrics in mouse and rat. (A) UMAP presentation of mouse biological replicates used in this study (n = 8 mice, 2 GEX+CITE and 6 GEX+ATAC). (B) Features of GEX+CITE samples. nFeature_RNA = number of genes per cell, nCount_RNA = number of unique molecular identifiers (UMIs) per cell, percent.mito = percentage of mitochondrial genes detected. (C) Features organized by cell type. (D) Features of GEX+ATAC samples by biological replicate: nCount_ATAC = number of fragments per cell, TSS.enrichment = enrichment around transcriptional start sites, nucleosome_signal = nucleosome signal. (E) Features organized by cell type. (F) UMAP presentation of rat GEX+CITE biological replicates used in this study (n = 12 rats). (G) Features of GEX+CITE samples. nFeature_RNA = number of genes per cell, nCount_RNA = number of unique molecular identifiers (UMIs) per cell, percent.mito = percentage of mitochondrial genes detected. (H) Features organized by cell type. (I) UMAP presentation of rat GEX+ATAC biological replicates (n = 4 rats). (J) Features of GEX+ATAC samples by biological replicate: nCount_ATAC = number of fragments per cell, TSS.enrichment = enrichment around transcriptional start sites, (K) Features organized by cell type. Unbiased clustering performed on mouse (L) and rat (M) datasets, combining both GEX + ATAC and GEX + ADT datasets for each species. Cell type assignments (N–O) for each species independently were based on marker gene expression.

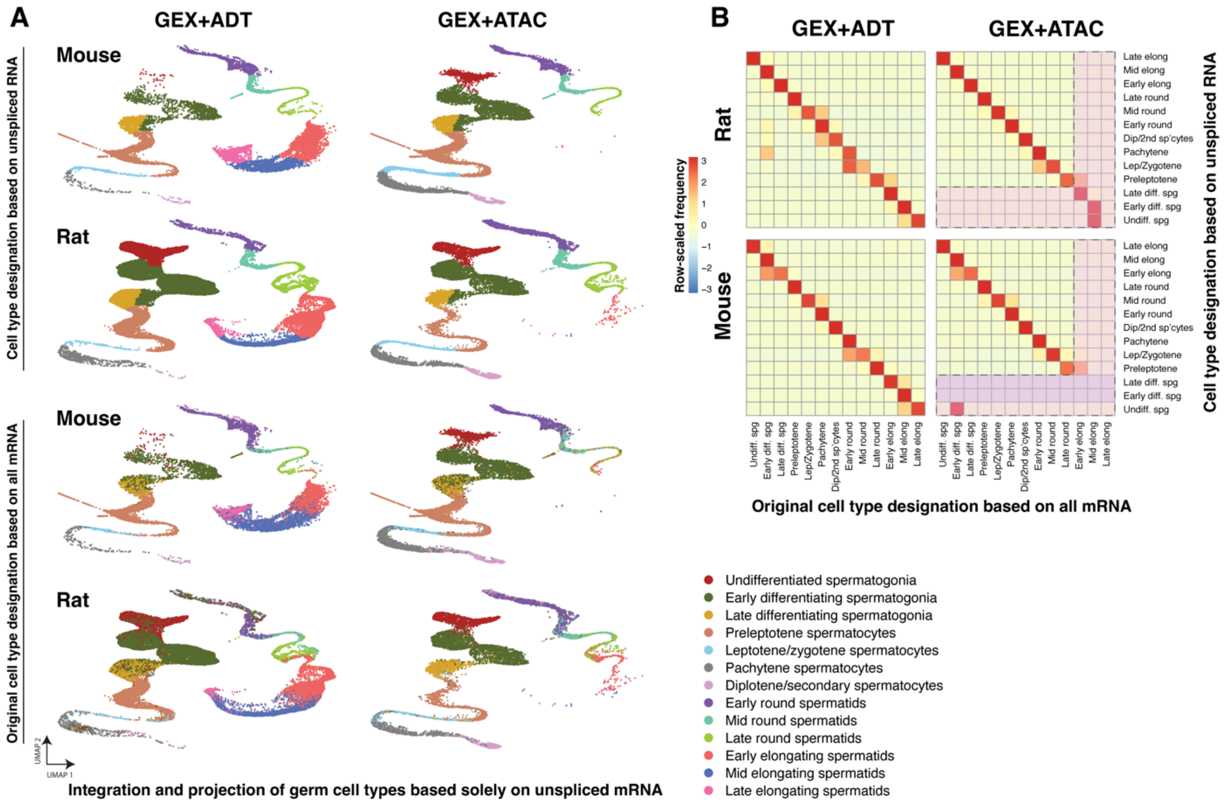

Figure S3: clustering of germ cells based on nuclear and whole-cell nascent unspliced mRNA. (A) UMAP showing distribution of germ cells based entirely on unspliced mRNA (n = 8 mice and 13 rats). Cell type designations are shown (top) assigned based only on nascent mRNA and (bottom) the same as Figure 1, based on whole cell/nuclear mRNA. (B) Comparison of cell designations based off whole cell/nuclear mRNA and based upon unspliced mRNA only. Shading of elongating spermatids in nuclear assay indicates few cells captured.

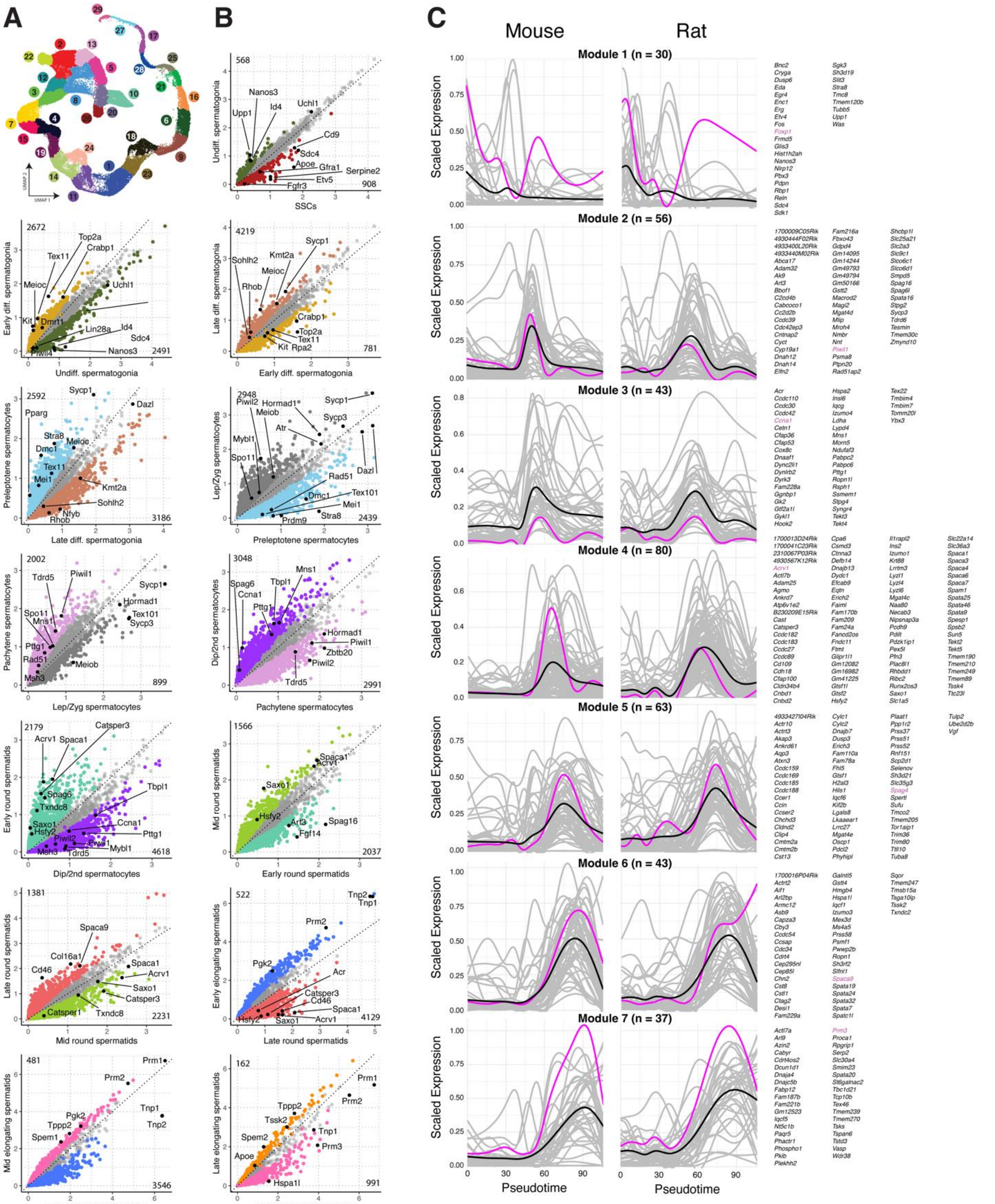
A
B
C
Mouse
Rat
Undiff. spermatogonia
SSCs
Early diff. spermatogonia
Late diff. spermatogonia
Preleptotene spermatocytes
Lep/Zyg spermatocytes
Pachytene spermatocytes
Dip/2nd spermatocytes
Early round spermatids
Mid round spermatids
Late round spermatids
Early elongating spermatids
Mid elongating spermatids
Late elongating spermatids
Module 1 (n = 30)
Module 2 (n = 56)
Module 3 (n = 43)
Module 4 (n = 80)
Module 5 (n = 63)
Module 6 (n = 43)
Module 7 (n = 37)
Scaled Expression
Pseudotime

Figure S4. Differential gene expression between cell types and modules of correlated gene expression between species. (A) Unbiased clustering of integrated mouse and rat germ cells. (B) Normalized gene expression shown for each pairwise comparison of sequential cell types. Significant (adjusted p value < 0.05) differentially expressed genes displaying >1.5-fold difference are colored by cell type. (C) Genes that correlate in expression between mouse and rat ($r > 0.9$, Pearson's correlation) were selected and modules of genes that behave similarly across pseudotime were calculated. For each module, expression (scaled to a percentage of maximum ex-pression) of all genes in the module by species is shown and one representative gene has been highlighted in magenta. All data in this figure were generated from sn/scRNA-seq (n = 8 mice and 13 rats).

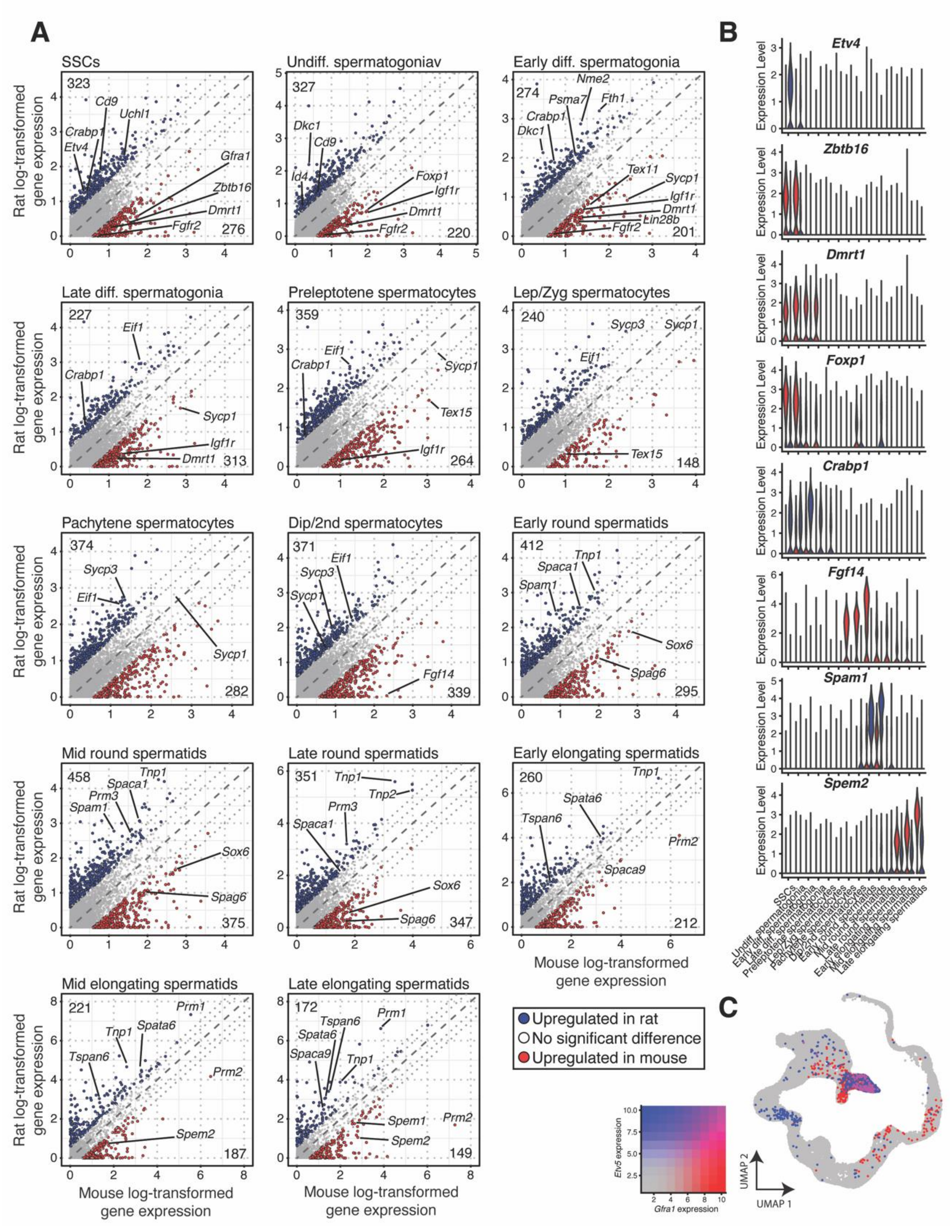

Figure S5. Differential gene expression between species. (A) Normalized gene expression shown for each cell type showing the pairwise comparison between mice and rats. Significant (adjusted p value < 0.05) differentially expressed genes showing a minimum of 10% difference in

proportion of cells expressing the gene as well as displaying >1.5-fold difference are colored. (B) Violin plots showing normalized gene expression of selected genes significantly different between species in at least one cell type. (C) Co-localization of *Etv5* and *Gfra1* in spermatogonia, colored by normalized gene expression. All data in this figure were generated from sn/scRNA-seq (n = 8 mice and 13 rats).

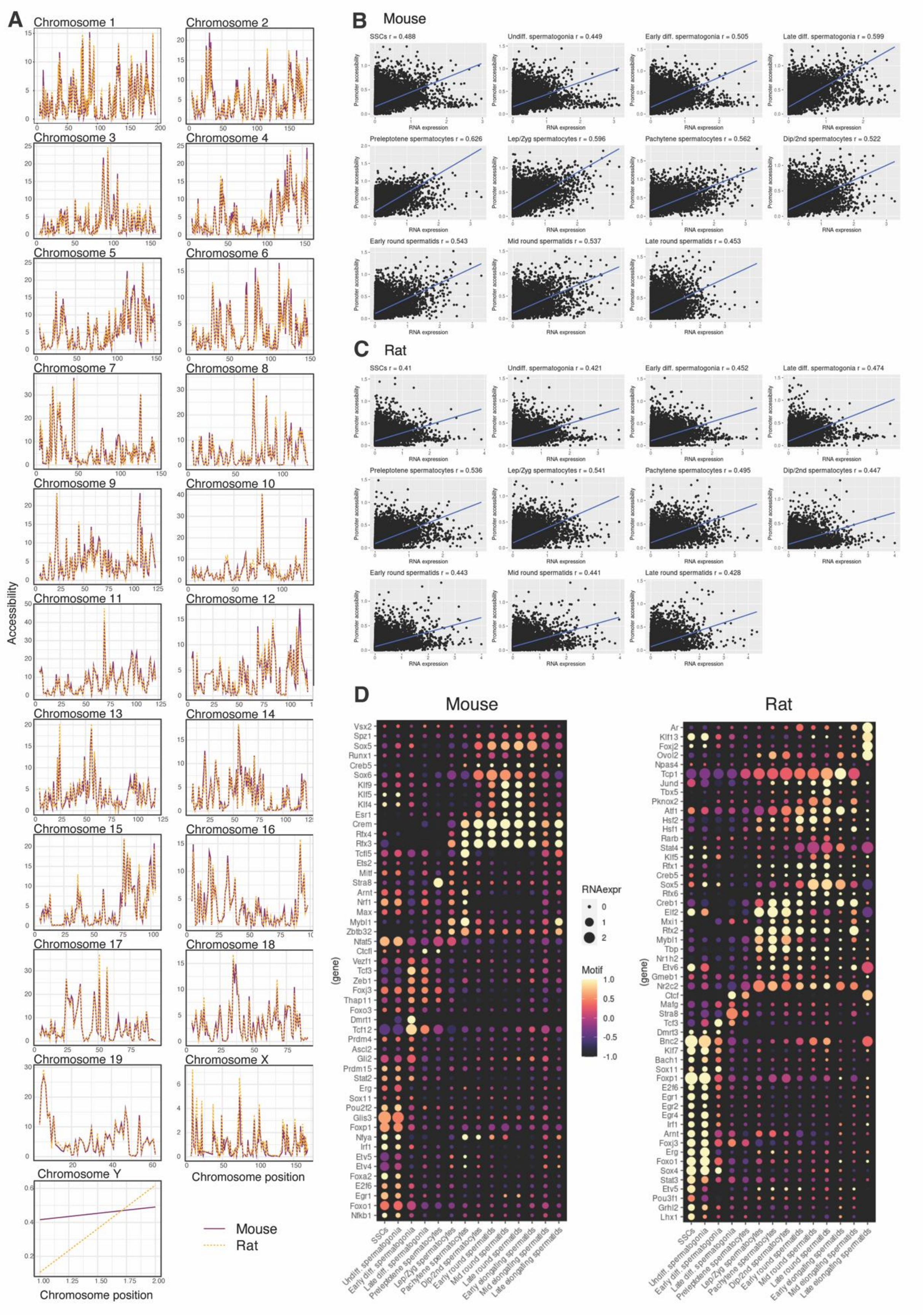
A
Chromosome 1
Chromosome 2
Chromosome 3
Chromosome 4
Chromosome 5
Chromosome 6
Chromosome 7
Chromosome 8
Chromosome 9
Chromosome 10
Chromosome 11
Chromosome 12
Chromosome 13
Chromosome 14
Chromosome 15
Chromosome 16
Chromosome 17
Chromosome 18
Chromosome 19
Chromosome X
Chromosome Y
Accessibility
Chromosome position
Mouse
Rat
B Mouse
SSCs r = 0.488
Undiff. spermatogonia r = 0.449
Early diff. spermatogonia r = 0.505
Late diff. spermatogonia r = 0.599
Preleptotene spermatocytes r = 0.626
Lep/Zyg spermatocytes r = 0.596
Pachytene spermatocytes r = 0.562
Dip/2nd spermatocytes r = 0.522
Early round spermatids r = 0.543
Mid round spermatids r = 0.537
Late round spermatids r = 0.453
Promoter accessibility
RNA expression
C Rat
SSCs r = 0.41
Undiff. spermatogonia r = 0.421
Early diff. spermatogonia r = 0.452
Late diff. spermatogonia r = 0.474
Preleptotene spermatocytes r = 0.536
Lep/Zyg spermatocytes r = 0.541
Pachytene spermatocytes r = 0.495
Dip/2nd spermatocytes r = 0.447
Early round spermatids r = 0.443
Mid round spermatids r = 0.441
Late round spermatids r = 0.428
D
Mouse
Rat
RNAexpr
Motif
(gene)
SSCs
Undiff. spermatogonia
Early diff. spermatogonia
Late diff. spermatogonia
Preleptotene spermatocytes
Lep/Zyg spermatocytes
Pachytene spermatocytes
Dip/2nd spermatocytes
Early round spermatids
Mid round spermatids
Late round spermatids
Early elongating spermatids
Mid elongating spermatids
Late elongating spermatids

73

Figure S6. Chromatin accessibility and gene expression. (A) Chromatin accessibility across all chromosomes. Each chromosome was divided into 10^6 bp bins and normalized peak counts were sampled and summed to the species minimum number of cells observed. (B) Correlation of promoter accessibility with mRNA expression in mouse and rat. Correlation coefficient (r) shown for each cell type, and blue line denotes linear model. (C) Transcription factor and motif accessibility correlation. Normalized expression of transcription factors selected with high motif correlation coeffects scaled across rows. Expression is denoted by size of dot and motif represented by chromVAR motif deviation score shown by color. All data in this figure were generated from snRNA/ATAC-seq multiomic profiling (n = 6 mice and 4 rats).

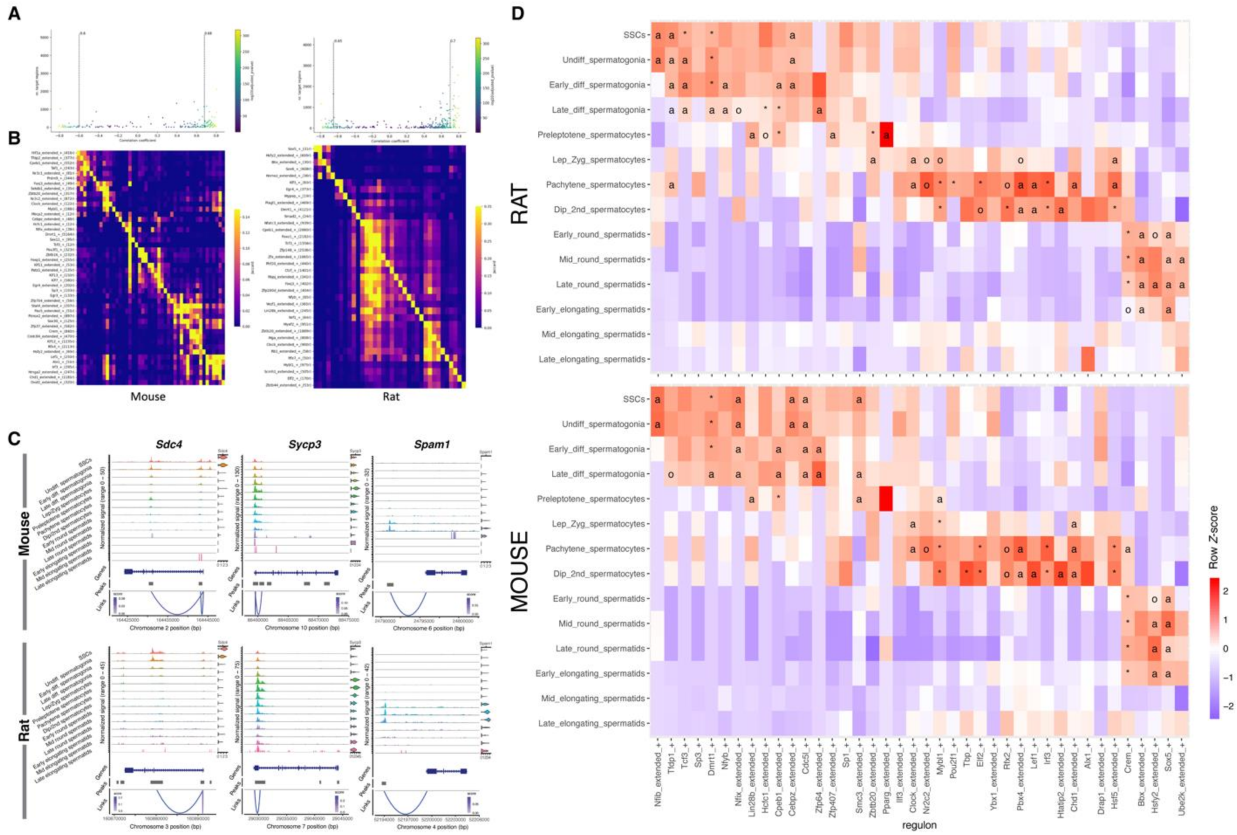

Figure S7. Cross-species conservation of gene regulatory networks in spermatogenesis. (A) Thresholds for GRN analysis. Scatter plot showing number of target regions versus TF expression-to-region AUC Pearson correlation coefficients for each regulon inferred for each species are shown. Regulons were filtered based on a threshold on the correlation coefficient, indicated by the dotted line as part of SCENIC+ pipeline. (B) Overlap of target regions of positive-regulated regulons by species. The overlap is divided by the number of target regions of the regulon in each row, yielding Jaccard similarity index. The color of the upper limit is set manually for visualization purposes. (C) Conserved putative regulatory regions in and around important spermatogenic genes. Regions matched by liftover of the rat genome onto mouse. Significant peak linkage shown. (D) Classification of chromatin effects. Heatmap of TF gene expression, overlaid with assessment of

TF activity, where “open” is a significant enrichment of TF cistromes in DARs and “active” indicates the mean AUC exceeds the regulon threshold. a = expression + active, o = expression + open, * = expression + active + open. All data in this figure were generated from snRNA/ATAC-seq multiomic profiling (n = 6 mice and 4 rats).

Target	Clone	Gene	Catalog number	Manufacturer	Type	Appl	ADT sequence
F4/80	BM8	*Adgre1*	TotalSeq™-B0114	Biolegend	Primary antibody	ADT	TTAACTTCAGCCCGT
IBA1	Polyclonal	*Aif1*	019-19741	Fujifilm Wako Chemicals	Primary antibody	ADT	-
Rat *Aif1*	-	*Aif1*	427068	ACD	RNA Probe	ISH	-
CD13	WM15	*Anpep*	TotalSeq™-B0364	Biolegend	Primary antibody	ADT	TTTCAACGCCCTTTC
CD317	927	*Bst2*	TotalSeq™-B0811	Biolegend	Primary antibody	ADT	TGTGGTAGCCCTTGT
CD274	MIH6	*Cd274*	TotalSeq™-B0190	Biolegend	Primary antibody	ADT	TCGATTCCACCAACT
CD52	HI186	*Cd52*	TotalSeq™-B0033	Biolegend	Primary antibody	ADT	CTTTGTACGAGCAAA
CD55	RIKO-3	*Cd55*	TotalSeq™-B0558	Biolegend	Primary antibody	ADT	ATTGTTGTCAGACCA
CD59	p282 (H19)	*Cd59*	TotalSeq™-B0361	Biolegend	Primary antibody	ADT	AATTAGCCGTCGAGA
CD9	MZ3	*Cd9*	TotalSeq™-B0813	Biolegend	Primary antibody	ADT	TAGCAGTCACTCCTA
CD9	2A1/CD9	*Cd9*	206506	Biolegend	Primary antibody	ADT/ FACS	-
Rat *Etv5*	-	*Etv5*	427068	ACD	RNA Probe	ISH	-
CD142	NY2	*F3*	TotalSeq™-B0822	Biolegend	Primary antibody	ADT	CACTGCCGTCGATTA
CD32	FUN-2	*Fcgr2a*	TotalSeq™-B0142	Biolegend	Primary antibody	ADT	GCTTCCGAATTACCG
Rat *Gfra1*	-	*Gfra1*	463031-C2	ACD	RNA Probe	ISH	-
GFRA1/CD326 (rat)	Polyclonal	*Gfra1*	BAF560	R&D Systems	Primary antibody	ADT/IHC/ MACS	-
GFRA1/CD326 (mouse)	98.8	*Gfra1*	118203	Biolegend	Primary antibody	ADT/ MACS	-
CD278	C398.4A	*Icos*	TotalSeq™-B0171	Biolegend	Primary antibody	ADT	CGCGCACCCATTAAA
Rat *Id4* (C2)	-	*Id4*	873098-C2	ACD	RNA Probe	ISH	-
KIT	2B8	*Kit*	TotalSeq™-B0012	Biolegend	Primary antibody	ADT	TGCATGTCATCGGTG
KIT	Polyclonal	*Kit*	ITA1763	G Biosciences	Primary antibody	ADT	-
CD205	HD30	*Ly75*	TotalSeq™-B0814	Biolegend	Primary antibody	ADT	CTATCGTTTGATGCA
CD56	5.1H11	*Ncam1*	TotalSeq™-B0047	Biolegend	Primary antibody	ADT	TCCTTTCCTGATAGG
CD273	24F.10C12	*Pdcd1lg2*	TotalSeq™-B0008	Biolegend	Primary antibody	ADT	TCAACGCTTGGCTAG
SPAM1	Polyclonal	*Spam1*	A2120	ABClonal Science Inc	Primary antibody	ADT	-
CD302	Polyclonal		LS-C119435	Lifespan Biosciences	Primary antibody	ADT	-
Biotin	-	-	TotalSeq™-B0952	Biolegend	Secondary antibody	ADT	CAGGTTGTTGTCATT
Biotin	-	-	TotalSeq™-B0953	Biolegend	Secondary antibody	ADT	GTCCGACTAATAGCT
Anti-APC	APC003	-	TotalSeq™-B0987	Biolegend	Secondary antibody	ADT	TTAACCGTCTCCCTT
Anti-PE	PE001	-	TotalSeq™-B0911	Biolegend	Secondary antibody	ADT	TGACCAGTTCCGCAT
Anti-FITC	FIT-22	-	TotalSeq™-B0988	Biolegend	Secondary antibody	ADT	-

Table S5. Antibodies and probes used in this study. "Target" denotes the reactive protein or mRNA. Appl = Application. ADT = antibody derived tags used for feature barcoding. IHC = immunohistochemistry. ISH = in situ hybridization. MACS = magnetically activated cell sorting. FACS = fluorescently activated cell sorting.

SUPPLEMENTARY METHODS

Selection of cell fractions

For rat samples selected for EpCAM, cells were incubated with mouse anti-rat EpCAM antibody (clone GZ1 produced by Dr Gottfried Dohr in the Medical University of Graz, Austria[1]) for 20 minutes at 4°C. Mouse samples instead were selected with anti-mouse EpCAM clone 98.8 (see Table S5). Samples were washed twice in PBS-S, resuspended and incubated with anti-mouse secondary antibody conjugated to magnetic microbeads (Miltenyi) for 20 minutes at 4°C. Cells were washed twice and selected via MACS MS columns (Miltenyi). Sample viability was typically 90-95% and no samples had viability below 80%. Flow cytometry was performed on a FACSCanto II Flow Cytometry System (BD Bioscience) and sorting on FACSAria II Cell Sorter (BD Bioscience) using the same antibodies as for ADT conjugated with APC/PE/FITC as appropriate (see Table S5) and for CD9 sorting the gating system is shown in **Figure 6M**.

Antibody labeling.

Cells were incubated with primary and secondary antibodies as described in Table A. 1-2 million cells were suspended per sample in 50 µl Cell Staining Buffer (Biolegend) with 0.5ul of TruStain FcX PLUS (anti-mouse CD16/32) Blocking Reagent. After 10 minutes of incubation at 4°C, an antibody cocktail containing all of the TotalSeq antibodies for that experiment. In the cases where an ADT secondary was used, the antibodies were stained in series with three washes in between to limit cross-reactivity. For each incubation, cells were incubated for 30 minutes at 4°C and then spun down at 400 × *rcf* followed by three washes. Washes were performed by adding 1ml for each wash and spinning down at 400 × *rcf.*

Nuclei preparation

Nuclei were prepared according to 10X's protocol CG000366 Rev B with changes as noted. Briefly, 10^5 cells were centrifuged at 500 × *rcf* for 5 minutes at 4C (all subsequent spins used the same conditions). 100ul of chilled 0.1X Lysis buffer was added, consisting of nuclease-free water with final concentrations after dilution: 10mM Tris-HCL (pH7.4, Sigma-Aldrich), 10mM NaCl (Sigma-Aldrich), 3mM $MgCl_2$ (Sigma-Aldrich), 0.01% Tween-20 (Thermo Fisher Scientific), 0.01% non-idet P40 substitute (Sigma-Aldrich), 0.001% digitonin (Thermo Fisher Scientific), 1% BSA (Miltenyi Biotech), 1mM DL-Dithiothreitol (DTT, Sigma-Aldrich), 1 U/μl sigma protector RNase inhibitor (Sigma-Aldrich). Cells were digested for 2 minutes on ice before resulting nuclei were pelleted. Nuclei were washed three times in a wash buffer identical to the lysis buffer lacking the P40 substitute and digitonin. Finally, nuclei were suspended in nuclei buffer (10X) with 1mM DTT and 1 U/ μl RNAse inhibitor.

Differential accessibility and pathway analysis

Differentially-accessible peaks were identified via Seurat's FindMarkers function with default options (i.e., the Wilcoxon rank-sum test). *TxDb.Mmusculus.UCSC.mm10.knownGene* and *TxDb.Rnorvegicus.UCSC.rn6.refGene* were used with ChIPseeker [2] to annotate peaks by relative locations to genes (promoter, intergenic, etc). All peak visualization was performed using Signac's CoveragePlot. Motif accessibility was calculated via ChromVAR [3] using the JASPAR2020 [4] set using Core collection and tax_group = vertebrates, and then using Signac's AddMotifs, FindMotifs and RunChromVAR with the appropriate genomes. Promoter activity was calculated with Signac's GeneActivity function using 2000bp upstream of the TSS and 0bp downstream and Pearson's correlations were performed against gene expression on a per-cell basis within each cell type.

Ingenuity Pathway Analysis (QIAGEN Inc, https://www.qiagenbioinformatics.com/products/ingenuity-pathway-analysis) was used for all pathway analyses. In all cases gene lists were used with a minimum fold change cutoff of ± 1.5 and *p*-adjusted value of ≤ 0.05.

Rat genome annotations

Rat genome annotations and peak calls were lifted to mouse coordinates using UCSC's LiftOver tool[5]. For both mouse and rat, many of the lncRNA names were arbitrary identifiers assigned after genome assembly. To coordinate orthologous lncRNAs, mouse lncRNA positions were queried against the lifted rat lncRNA coordinates with PyRanges[6]. If multiple mouse lncRNAs overlapped a single, lifted rat lncRNA, then the gene with largest %overlap was selected as the orthologous annotation. To assess chromosome-scale accessibility, the positions of lifted rat peaks and overlapping mouse peaks were organized into 10^6 bp bins. For each cell type, normalized peak counts were sampled to the specie minimum number of cells observed and summed. To analyze expression of individual *Gfra1* exons, a second rat reference was created where each exon of *Gfra1* was annotated in the GTF as a separate gene and counts calculated for all cells and processed in the same manner as the main analysis, but only used for the purpose of *Gfra1* visualization.

Transcriptional regulatory analysis with SCENIC+

The TRN analysis was conducted using SCENIC+ v1.01.dev3+g3741a4b[7]. First, custom cisTarget databases were created by extracting the genomic sequences for each peak feature in the snATAC-seq component of the multiome data, using the mm10 or rn6 genomes for mouse and rat

respectively, and then compiling the databases using the create_cistarget_motif_databases.py tool (https://github.com/aertslab/create_cisTarget_databases). The SCENIC+ pipeline was run largely in accordance with the suggested protocol for 10x multiome data. Briefly, 20 topics were selected for the snATAC-seq data following topic modeling for both mouse and rat and were binarized with both the otsu method and with ntop=3000. The motifs-v10-nr.mgi-m0.00001-o0.0.tbl database was used for the mouse motif annotation. The rat motif annotation was constructed by converting each gene to the closest matching rat ortholog. The run_pycistarget function was implemented with run_without_promoters=True, followed by the run_scenicplus function with upstream=downstream=[1000,150000]. The rat transcription factor collection was obtained through converting mouse transcription factors to the closest matching ortholog. Regulons were filtered qualitatively to retain approximately 80 eRegulons for mouse and rat each. Pseudotime plots were constructed using the plot_potential function with the joint Monocle pseudotime values.

Transcription factor (TF) activity was calculated using methods described by Garcia-Alonso *et al.* [8] adapted for SCENIC+ methods. The criterion for TF expression was as described by Garcia-Alonso *et al.* [8] "Open" TF is defined by significant enrichment of TF cistromes in cell type-specific differentially accessible regions (DARs) as scored by pycistarget[7]. For RNA-based activity, we consider the empirically determined gene targets of each TF regulon. If the mean AUC score for a given cell type is greater than the regulon threshold, then the TF is "active."

Transplantation

Rat samples were enriched for CD9+ cells as described above. Transplants were performed at 3×10^6 cells per ml into NU/J nude mice (Jackson Laboratories 002019). Transplantation procedure was performed as described previously[9]. Host animals were sacrificed after 4 months

according to institutional guidelines. Testes were extracted, weighed and the tunica removed. For encapsulation, cells were prepared as described above. For colony counting, transplanted testes were stained with X-gal as described previously[10].

Immunohistochemistry and *in situ* RNA hybridization

One 4-month-old male HomoMTLacZ rat was sacrificed according to university animal use protocols. Both right and left testes were removed and placed individually into 2 mL of Hank's balanced salt solution (Gibco #14175079) in well of a 6 well culture plate. The tunica albuginea was removed, and the tissue was transferred into a 50 mL tube filled to 50 mL with 10% formalin (Fisher #23245684), then shaken slowly for 24 hours at room temperature. Tissue was dehydrated by a standard ethanol series followed by xylene, then finally embedded in paraffin using standard procedures. Paraffin blocks were sectioned at 5 um using a microtome. Staging of the seminiferous tubules was performed according to Russel *et al.* 1993[11] using FFPE sections stained with hematoxylin-eosin (HE) and Periodic acid-Schiff (PAS). HE and PAS histochemistry was performed following the protocols of the Armed Forces Institute of Pathology[12].

For multiplex *in situ* hybridization (ISH), the RNAscope® technology was employed. RNAscope® (ISH) was carried out on a Leica BOND RXm platform according to manufacturer's protocols. Probes used are detailed in Table S5. Multiplex RNAscope® probes and antibody codetection was also carried out on a Leica BOND RXm platform according to manufacturer's protocols. Immunohistochemistry/immunofluorescence was performed as described elsewhere[13] using the primary antibodies listed in Table S5.

1. Schiechl, H. & Dohr, G. Immunohistochemical studies of the distribution of a basolateral-membrane protein in intestinal epithelial cells (GZ1-Ag) in rats using monoclonal antibodies. *Histochemistry* **87**, 491-498 (1987).
2. Wang, Q. *et al.* Exploring Epigenomic Datasets by ChIPseeker. *Curr Protoc* **2**, e585 (2022).
3. Schep, A.N., Wu, B., Buenrostro, J.D. & Greenleaf, W.J. chromVAR: inferring transcription-factor-associated accessibility from single-cell epigenomic data. *Nature methods* **14**, 975-978 (2017).
4. Rauluseviciute, I. *et al.* JASPAR 2024: 20th anniversary of the open-access database of transcription factor binding profiles. *Nucleic Acids Res* **52**, D174-D182 (2024).
5. Perez, G. *et al.* The UCSC Genome Browser database: 2025 update. *Nucleic Acids Res* (2024).
6. Stovner, E.B. & Saetrom, P. PyRanges: efficient comparison of genomic intervals in Python. *Bioinformatics* **36**, 918-919 (2020).
7. Bravo Gonzalez-Blas, C. *et al.* SCENIC+: single-cell multiomic inference of enhancers and gene regulatory networks. *Nature methods* **20**, 1355-1367 (2023).
8. Garcia-Alonso, L. *et al.* Single-cell roadmap of human gonadal development. *Nature* **607**, 540-547 (2022).
9. Sinha, N., Whelan, E.C. & Brinster, R.L. Isolation, Cryopreservation, and Transplantation of Spermatogonial Stem Cells. *Methods Mol Biol* **2005**, 205-220 (2019).
10. Nagano, M., Avarbock, M.R. & Brinster, R.L. Pattern and kinetics of mouse donor spermatogonial stem cell colonization in recipient testes. *Biology of reproduction* **60**, 1429-1436 (1999).
11. Russell, L.D., Ettlin, R.A., Hikim, A.P.S. & Clegg, E.D. Histological and Histopathological Evaluation of the Testis. *International Journal of Andrology* **16**, 83-83 (1993).
12. LH, P.E.M.B.S. *Laboratory Methods in Histotechnology*. (American Registry of Pathology, Washington DC, USA; 1992).
13. Tarrant, J.C. *et al.* Pathology of macrophage activation syndrome in humanized NSGS mice. *Res Vet Sci* **134**, 137-146 (2021).
